# Important cardiac transcription factor genes are accompanied by bidirectional long non-coding RNAs

**DOI:** 10.1186/s12864-018-5233-5

**Published:** 2018-12-27

**Authors:** Yutaro Hori, Yoko Tanimoto, Satoru Takahashi, Tetsushi Furukawa, Kazuko Koshiba-Takeuchi, Jun K. Takeuchi

**Affiliations:** 1 Laboratory of Cell Growth and Differentiation, Institute of Molecular and Cellular Biosciences, the University of Tokyo, Hongo, Bunkyo, Tokyo, Japan; 2 Division of Cardiovascular Regeneration, Institute of Molecular and Cellular Biosciences, the University of Tokyo, Hongo, Bunkyo, Tokyo, Japan; 3 Department of Biological Sciences, Graduate School of Science, the University of Tokyo, Hongo, Bunkyo, Tokyo, Japan; 4 Laboratory Animal Resource Center, University of Tsukuba, Tsukuba, Ibaraki, Japan; 5 Department of Anatomy and Embryology, Faculty of Medicine, University of Tsukuba, Tsukuba, Ibaraki Japan; 6 Department of Applied Biosciences, Faculty of Life Sciences, Toyo University, Itakura, Gunma Japan; 7 Division of Bio-informational Pharmacology, Medical Research Institute, Tokyo Medical and Dental University, Tokyo, Japan

**Keywords:** Heart development, Long non-coding RNA, Haploinsufficiency, Bidirectional promoter

## Abstract

**Background:**

Heart development is a relatively fragile process in which many transcription factor genes show dose-sensitive characteristics such as haploinsufficiency and lower penetrance. Despite efforts to unravel the genetic mechanism for overcoming the fragility under normal conditions, our understanding still remains in its infancy. Recent studies on the regulatory mechanisms governing gene expression in mammals have revealed that long non-coding RNAs (lncRNAs) are important modulators at the transcriptional and translational levels. Based on the hypothesis that lncRNAs also play important roles in mouse heart development, we attempted to comprehensively identify lncRNAs by comparing the embryonic and adult mouse heart and brain.

**Results:**

We have identified spliced lncRNAs that are expressed during development and found that lncRNAs that are expressed in the heart but not in the brain are located close to genes that are important for heart development. Furthermore, we found that many important cardiac transcription factor genes are located in close proximity to lncRNAs. Importantly, many of the lncRNAs are divergently transcribed from the promoter of these genes. Since the lncRNA divergently transcribed from *Tbx5* is highly evolutionarily conserved, we focused on and analyzed the transcript. We found that this lncRNA exhibits a different expression pattern than that of *Tbx5*, and knockdown of this lncRNA leads to embryonic lethality.

**Conclusion:**

These results suggest that spliced lncRNAs, particularly bidirectional lncRNAs, are essential regulators of mouse heart development, potentially through the regulation of neighboring transcription factor genes.

**Electronic supplementary material:**

The online version of this article (10.1186/s12864-018-5233-5) contains supplementary material, which is available to authorized users.

## Background

Morphogenesis is a complex process in which appropriate cell types are differentiated and positioned at the right place and at the proper timing. The surprising reproducibility of developmental processes is underpinned by the robustness of the genetic program [1]. However, in spite of the high robustness under normal genetic conditions, the program can be easily collapsed by genetic abnormalities; for example, some genes require both alleles for proper function (i.e., haploinsufficiency) [2]. This type of fragility is frequently observed in mammalian heart development. In the heart, even a slight alteration of the program leads to congenital heart diseases (CHDs) and this fact is associated with the high frequency of CHDs, which is around one in one hundred births [3]. Genetic studies have shown that many of the transcription factor genes involved in the heart development are regulated in a highly spatiotemporal manner [4]. However, how such an intricate control of gene expression is achieved has not been well understood.

Comparative genomics have shown that the complexity of the body plan and the proportion of non-coding regions in the genome are positively correlated [5]. While most of the non-coding regions have previously been considered as “junk”, it is now accepted that some of them are necessary for the regulation of genes in a fine and complicated way [6]. Many evo-devo studies support this view, suggesting that the evolution of multicellular organisms was largely driven by the adjustments in transcriptional regulators, such as enhancer elements, rather than by functional evolution of protein-coding genes [7]. Recent advancements in genomics and transcriptomics have demonstrated that nearly half of the mammalian genome is actually transcribed into RNAs [8]. Long non-coding RNA (lncRNA) is an emerging class of RNA that is generally defined as RNAs longer than 200 nucleotides that lack the ability to produce functional proteins. Many of these molecules have been demonstrated to work as transcriptional or translational regulators [9]. Some lncRNAs are known to recruit epigenetic regulators to specific loci in the genome to modulate transcription. For example, a classical lncRNA, *Xist,* recruits Polycomb repressive complex 2 (PRC2) to the X chromosome in *cis* to inactivate one of the two X chromosomes to achieve dosage compensation [10]. Many lncRNAs studied thus far have been found to bind epigenetic factors and recruit them to defined genomic loci; however, not a small proportion of proposed lncRNA-PRC interactions have been suggested to be non-specific [11, 12]. Other lncRNAs function as post-transcriptional modulators of gene expression through the formation of duplexes with mRNA to inhibit translation by RNAi (i.e., antisense transcripts) [13], through the inhibition of miRNAs by working as so-called sponges [14] or by controlling splicing [15]. Although much attention has been paid to lncRNAs recently, the low conservation of sequences across species and the difficulty of determining their three-dimensional structures make it difficult to functionally and evolutionarily classify these molecules. Their biochemical characteristics (e.g., strong nonspecific binding to proteins) also make it difficult to dissect their precise molecular functions [12, 16]. Many lncRNAs show stage- and tissue-specific expression patterns, suggesting their roles in development [17].

Although several lncRNAs that function in mammalian heart development have been reported, the identification and characterization of lncRNAs in the mammalian heart are still insufficient [18–23]. Considering the regulatory nature of lncRNAs, they are thought to be key components in solving the aforementioned problems regarding the developmental fragility in mammalian hearts.

Here, we report that key cardiac transcription factors genes are located in close proximity to genes encoding lncRNAs. Interestingly, there are transcription factor and lncRNA pairs that are bidirectionally transcribed from the same promoter. We have focused on one lncRNA near *Tbx5*, which we call *Tbx5ua,* and showed that it is required for heart development. *Tbx5ua*-knockdown mice showed abnormally thin ventricular walls and were embryonic lethal.

## Results

### Identification of lncRNAs that are expressed during mouse heart development

To identify novel lncRNAs that are specifically expressed during heart development in mice, we extracted total RNA from the ventricles of embryonic day (E) 10.5 and E13.5 and 8 weeks-old mice and prepared cDNA libraries, that were subjected to paired-end 2 * 100 bp RNA-seq. The resulting read count was approximately 40 M reads for each sample. The obtained reads were mapped to the mouse genome (mm10) with Tophat2 [24], and the mapped reads were assembled using Cufflinks [25] with and without UCSC transcript annotations. Because many of the currently known functional lncRNAs are spliced and because it is difficult to confirm the existence of non-spliced transcripts unless they are expressed at very high levels, we focused on spliced lncRNA candidates in our analysis. We set the lower limit of expression at a fragments per kilobase of exon per million mapped fragments (fpkm) of 1, because above that level, the accuracy of the reconstruction of known transcripts without the transcript reference was sufficiently high (Additional file 1). We also checked if exons of known genes are mistaken as lncRNAs. We found that the direction of a majority of the lncRNAs that are located within 10,000 bp from known genes are in the opposite direction from them (225 vs 86), suggesting that such mis-annotations are rare.

**Fig. 1:**
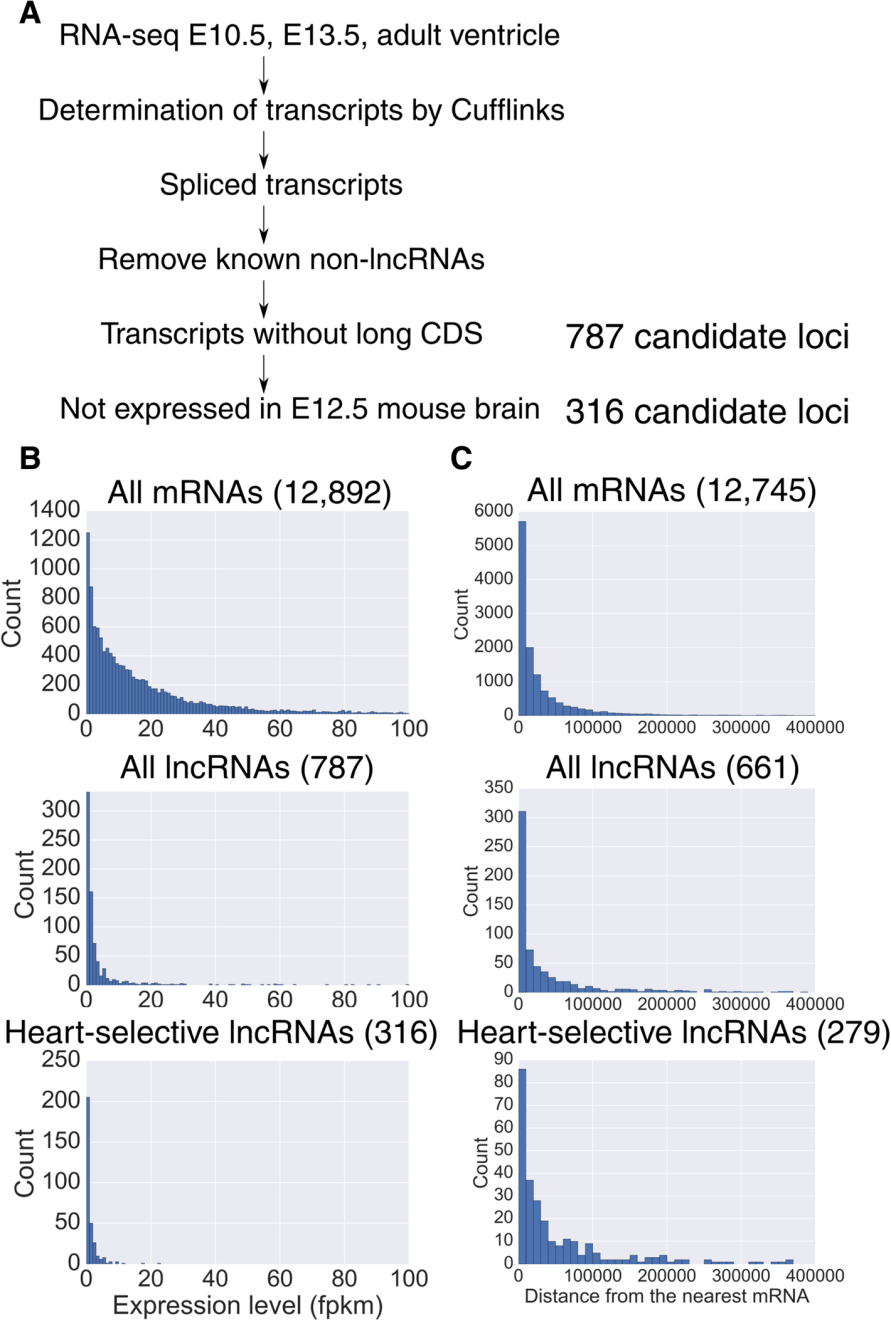
The screening procedure of lncRNAs. **a** The flowchart for the identification of lncRNAs expressed in the mouse heart. **b** The histograms of expression levels (fpkm) at E10.5. The number of genes expressed at E10.5 is shown in each parenthesis. The expression levels of lncRNAs are generally lower and lncRNAs with tissue-specificity rarely exceed 10 fpkm (red circle). **c** The distances from the nearest protein-coding genes were calculated and the distributions were plotted based on RNA-type. We require genes to be expressed in the ventricle at a minimum of one stage. The number of genes in each category is shown in the parenthesis. Heart-selective genes were generally located at greater distances

From the assembled transcripts, already known mRNAs or functional RNAs that are not generally classified as lncRNAs (e.g., snoRNA and tRNA) were removed, and we also omitted RNAs that have CDS longer than 1/3 of their total length according to the standard of Ensembl, since they are potentially protein-coding transcripts. As a result, we were able to identify 787 candidates of spliced lncRNAs. To omit lncRNAs that are ubiquitously expressed without tissue specificity, we examined the expression of the obtained candidates in the mouse brain. Because the brain is an organ that diverges from the heart at a very early developmental stage and originates from the ectoderm, whereas the heart originates from the mesoderm, we used the brain as a reference organ. We here just wanted to exclude lncRNAs that are expressed with no tissue specificity and did not intended to find lncRNAs that are exclusively expressed in the heart since many genes are known to function differently according to the context of the tissues. The comparison revealed that 316 of the identified spliced lncRNA candidates were selectively expressed in the heart (Fig. 1a, Additional file 2: Table S1). We checked the expression of these genes in the kidney and the liver and found that only 34 were expressed in both of them, and 213 of them were expressed only in the heart. We found that some lncRNA candidates were expressed in a stage-specific manner, suggesting that they may have roles in heart development or maturation.

### Many of the cardiac transcription factor genes have neighboring lncRNAs

First, we plotted the distribution of the expression levels of the obtained lncRNAs at E10.5 along with that of mRNAs. Consistent with the previous reports, the expression levels of lncRNAs were much lower than those of mRNAs. Interestingly, almost no heart-selective lncRNAs had fpkm values higher than 10 (Fig. 1b). Since many lncRNAs are known to modulate the transcription of neighboring genes in *cis*, we tried to identify the neighboring genes of the identified lncRNAs. The distribution of the distances from the transcriptional start site (TSS) of lncRNAs to the nearest genes was examined (Fig. 1c). Overall, the distance distribution of all obtained lncRNAs seemed to be similar to that of mRNAs. However, heart-selective lncRNAs were unexpectedly found to be at greater distances to protein-coding genes. The median distances were 12,626, 12,024 and 22,522 for mRNAs, all lncRNAs and heart-selective lncRNAs, respectively (*p* ≈ 3.9 * 10^− 1^ for all lncRNAs vs. mRNAs; and *p* ≈ 8.4 * 10^− 8^ for heart-selective lncRNAs vs. mRNAs, Mann-Whitney U test). Next, we examined what types of genes were enriched among the genes closest to lncRNAs. To this end, we conducted a gene enrichment analysis on such protein coding genes using the DAVID bioinformatics tool (http://david.ncifcrf.gov/) [26] and found that transcription factor genes were enriched among genes near lncRNAs in the heart. We also found that the genes associated with heart development were more strongly enriched among the genes near heart-selective lncRNAs when compared to the genes near lncRNAs lacking tissue specificity (Tables 1, 2).

**Table 1:** Gene ontology analysis of the genes closest to all lncRNAs that are expressed in the heart

Term	Count	%	*p*-value
GO:0006355~regulation of transcription, DNA-templated	86	14.42	8.51E-10
GO:0006351~transcription, DNA-templated	72	12.07	2.09E-08
GO:0045944~positive regulation of transcription from RNA polymerase II promoter	44	7.38	5.85E-07
GO:0000122~negative regulation of transcription from RNA polymerase II promoter	33	5.53	1.31E-05
GO:0060348~bone development	8	1.34	9.50E-05

Transcription factor genes were enriched among the genes close to lncRNAs. GO terms with false discovery rate (FDR) < 0.05 were sorted by the enrichment *p*-value

**Table 2:** Gene ontology analysis of the genes closest to heart-selective lncRNAs

Term	Count	%	*p*-value
GO:0045944~positive regulation of transcription from RNA polymerase II promoter	40	9.70	2.26E-09
GO:0007507~heart development	19	4.61	1.97E-08
GO:0000122~negative regulation of transcription from RNA polymerase II promoter	31	7.52	6.80E-08
GO:0051891~positive regulation of cardioblast differentiation	5	6.55	1.65E-07
GO:0003151~outflow tract morphogenesis	8	1.94	8.14E-06
GO:0060413~atrial septum morphogenesis	5	1.21	3.01E-05
GO:0060347~heart trabecula formation	5	1.21	4.06E-05

Genes associated with heart development are strongly enriched among genes closest to lncRNAs that are selectively expressed in the heart. Similar terms were omitted

**Fig. 2:**
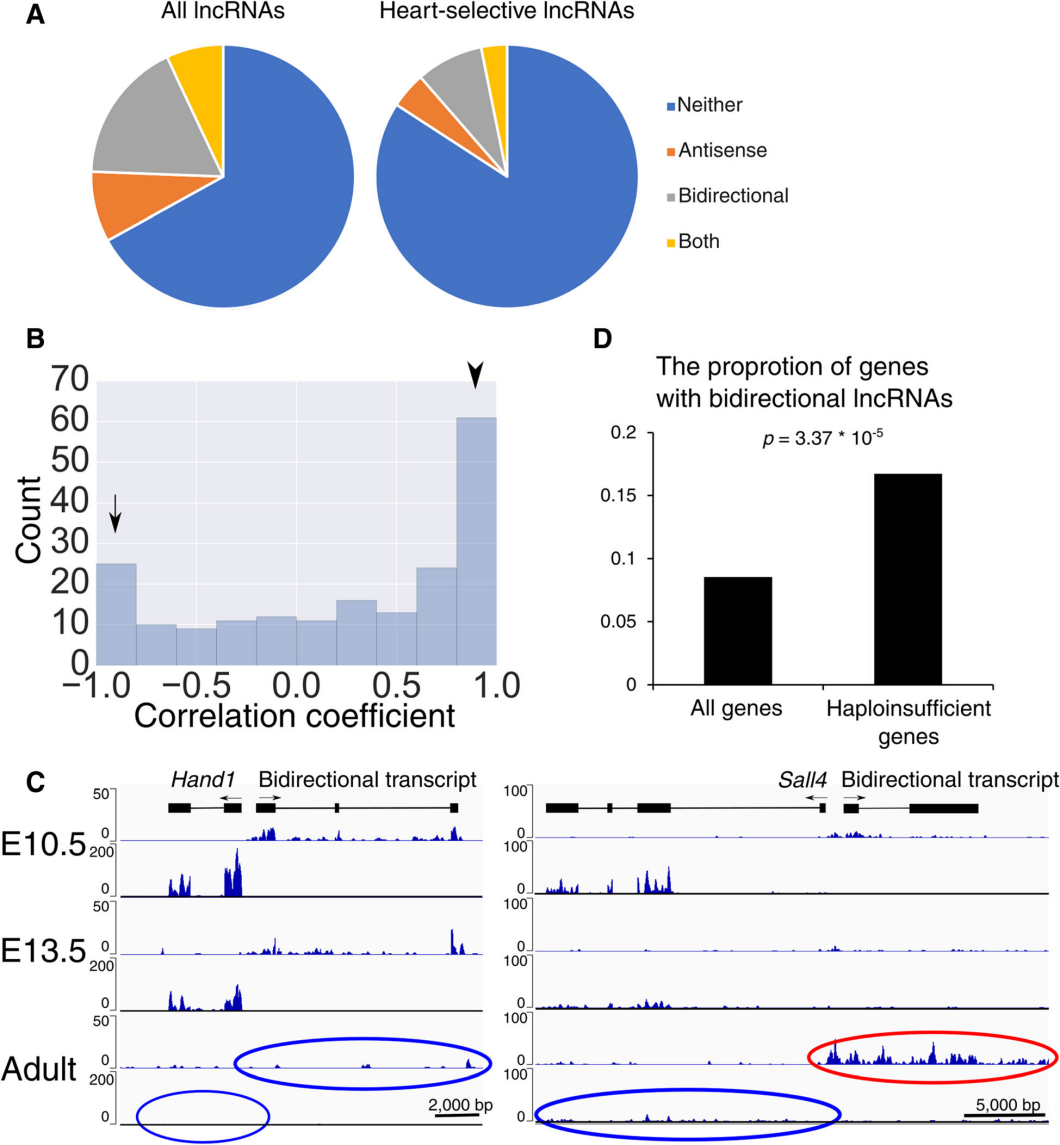
lncRNAs are enriched near genes that are important for heart development. **a** Classification of the lncRNA candidates found in the screen. Heart-selective lncRNAs are less likely to be bidirectional or antisense lncRNAs. Some lncRNAs were judged to be both antisense and bidirectional due to alternative promoter isoforms. **b** Distribution of the Pearson correlation coefficients between the bidirectional promoter pairs over the course of development. The arrow indicates a negative correlation and the arrowhead indicates a positive correlation. **c** *Hand1* shows a correlated expression pattern with its bidirectional lncRNA (left), while *Sall4* exhibits the opposite trend (right). **d** The proportion of genes that possess bidirectional lncRNAs in the mouse were examined by referring to RefSeq and a paper that identified haploinsufficient genes. Bidirectional lncRNAs were significantly enriched among haploinsufficient genes

We next tried to identify antisense lncRNAs and lncRNAs from bidirectional promoters. Bidirectional promoters produce two transcripts in a head-to-head divergent manner and attract a lot of attention as important sources of lncRNAs. Preceding studies have revealed that many of them regulate the genes with which they share promoters. We evaluated lncRNAs that had their TSS within 3000 bp from the promoter of protein coding genes as lncRNAs driven by bidirectional promoters. Consistent with the result that the distance between heart-selective lncRNAs and their neighboring genes is generally greater than the distance between all lncRNAs and their neighboring genes, both antisense lncRNA and bidirectional lncRNA were enriched among lncRNAs that are expressed both in the heart and the brain (Fig. 2a) (Additional file 3: Table S2 and Additional file 4: Table S3). Some of the lncRNAs and neighboring genes were judged to be both antisense and bidirectional because of alternative promoter isoforms.

Next, in order to clarify the relationship between mRNAs and their bidirectional lncRNAs, we calculated Pearson correlation coefficients between the log2-transformed expression levels of the bidirectional promoter pairs over the course of development. The distribution of the correlation coefficients is plotted in Fig. 2b. Many gene pairs clearly show positive or negative correlation, and the positive correlation appears to be dominant (Fig. 2c).

By searching the protein coding genes that are close to lncRNAs, we found many transcription factor genes that have critical functions for heart development (i.e., *Tbx5, Tbx20, Nkx2–5, Gata4, Gata6, Sall4, Hand1, Hand2, Wt1, Nr2f1, Irx3* and *Irx5*). Notably many of these lncRNAs were bidirectional lncRNAs (i.e., *Tbx5, Tbx20, Nkx2–5, Gata6, Sall4, Hand1, Hand2, Wt1, Nr2f1, Irx3* and *Irx5*). Some of these lncRNAs (e.g., those divergent to *Irx5, Gata6 and Wt1*) are expressed in the kidney or in the liver, and in such cases divergent genes are also expressed, suggesting that the expression of bidirectional pairs are correlated not only temporally but spatially. We examined the conservation of these lncRNAs near transcription factors by searching the RefSeq database and found that at least some lncRNAs were conserved in the human genome (*Tbx5, Nkx2–5, Hand2, Gata6, Wt1* and *Nr2f1*) (Additional file 5) and that the bidirectional lncRNA to *Tbx5* (*Lnc125*) was even conserved in chicken, which diverged from mammals 400 million years ago. Here, we judged bidirectional lncRNAs to be conserved solely based on the existence of transcripts at the corresponding loci, since the sequences of lncRNAs are known to evolve rapidly.

Because haploinsufficient transcription factor genes seem to be highly enriched among the genes that are in close proximity to divergent lncRNAs, we determined whether the enrichment was limited to the heart or whether it was more generally true [27, 28]. Using the mouse RefSeq transcript database (GRCm38.p3) and a paper that comprehensively identified haploinsufficient genes, we tried to determine the proportion of genes with bidirectional lncRNAs among all genes and among haploinsufficient genes [29] (Additional file 6: Table S4). We indeed found that haploinsufficient genes were significantly more enriched among genes with bidirectional lncRNAs (*p* = 3.4 * 10^− 5^ based on hypergeometric distribution) (Fig. 2d). To exclude the possibility that the tissue specificity of bidirectional lncRNAs and haploinsufficient genes generates pseudo-correlations, we calculated the proportion of housekeeping genes among all genes and among haploinsufficient genes and showed that the proportions were not significantly different (Additional file 7) [30].

Generally, the conservation of lncRNAs across species is very low compared to protein-coding transcripts. However, the *Tbx5*-divergent lncRNA is observed among a wide range of species. *Tbx5* is also a dosage-sensitive gene [27]. These findings prompted us to examine the function of the *Tbx5*-divergent lncRNA.

### Analysis of the *Tbx5*-divergent lncRNA

*Tbx5* is a transcription factor that is known to be essential for the development of the heart and forelimb. Holt-Oram syndrome is a dominant disorder caused by a single-allele mutation of *TBX5* and is characterized by hypoplasia of the forelimb, abnormalities in the thumb, and atrial and/or ventricular septal defects [31–33]. Importantly, the phenotypes of Holt-Oram syndrome show a high degree of variance, indicating that the dose of *TBX5* is crucial in normal heart development [34].

**Fig. 3:**
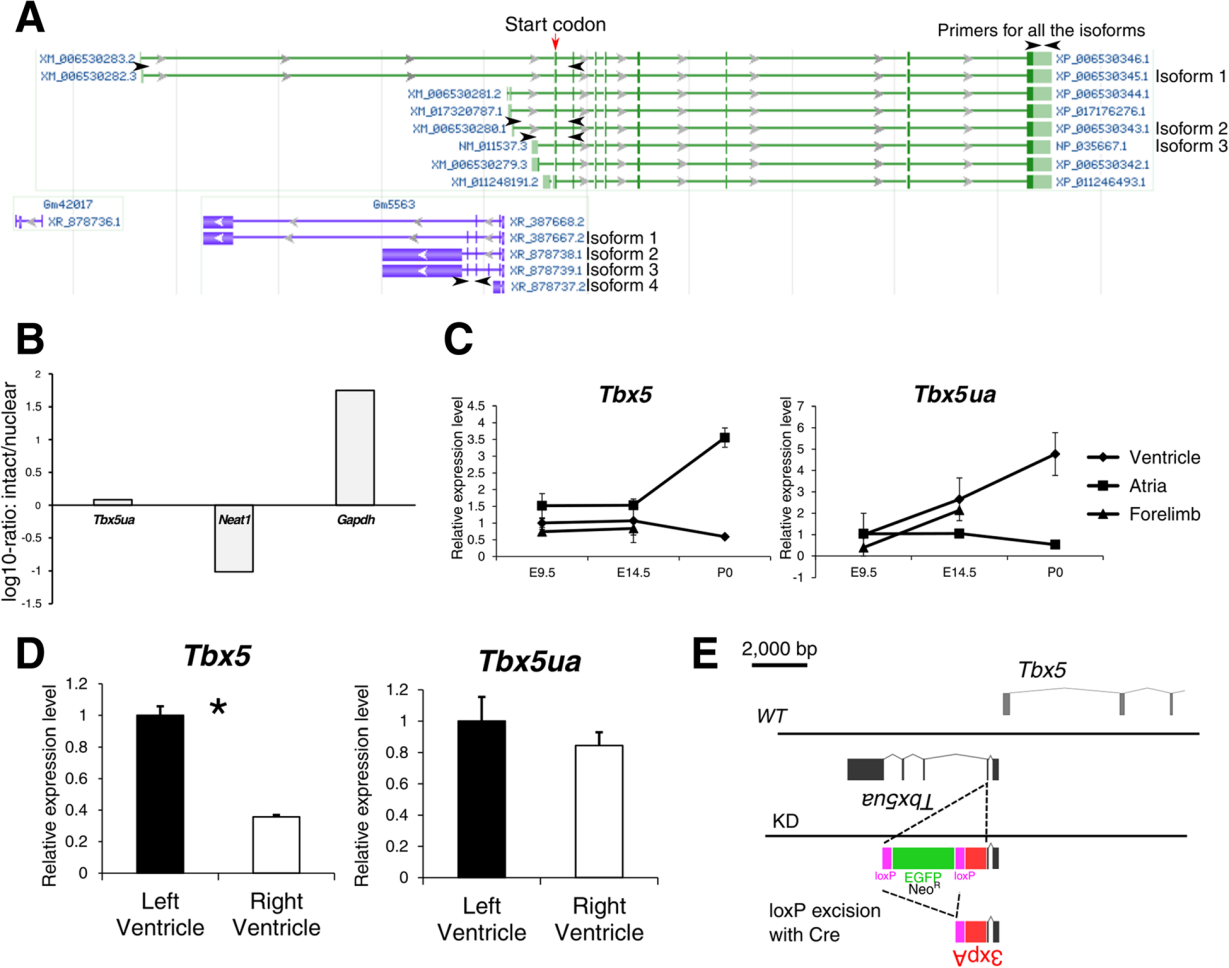
Different expression patterns of *Tbx5ua* and *Tbx5.* **a** RefSeq genome annotation of *Tbx5* locus. The isoforms of *Tbx5* and *Tbx5ua* that were identified in our RNA-seq analysis were labeled with isoform numbers. The qPCR primers used to quantify *Tbx5* and *Tbx5ua* are indicated as arrowheads. **b** The log10-ratios of intact/nuclear RNA abundances. *Gapdh* and *Neat1* serve as controls for cytoplasmic and nuclear localized RNA, respectively. **c** The expression levels of *Tbx5* and *Tbx5ua* during development as determined by qRT-PCR. In the ventricle, the expression level of *Tbx5ua* is increased with the progression of development while that of *Tbx5* is decreased (*n* = 3). **d** The expression levels of *Tbx5* and *Tbx5ua* in the left and right ventricles at E11.5 as determined by qRT-PCR. Unlike *Tbx5*, *Tbx5ua* was equally expressed in both ventricles (n = 3, *: *p* < 0.05, Welch's *t*-test). **e** Schematic diagram of the *Tbx5ua* knockdown experiment. Three tandem copies of bovine growth hormone polyadenylation signal were inserted along with the neomycin resistance gene (*Neo*^*R*^) or *EGFP*. The selection markers were subsequently removed with cell-permeable Cre recombinase

Hereafter we will call this lncRNA as *Tbx5 upstream antisense product* (*Tbx5ua*). *Tbx5ua* homolog is present in human genome annotation and it is named *TBX5-AS1* (Additional file 5). When compared with humans *TBX5-AS1* the sequence of *Tbx5ua* is relatively well conserved at the 5′ region, although it is hard to judge if this conservation is the consequence of functional demand since both the promoter and enhancer elements also exhibit a high degree of conservation (Additional file 8). *Tbx5ua* is transcribed from one of the promoters of *Tbx5* in the opposite direction and overlaps with the intron of one of the *Tbx5* isoforms (Fig. 3a, RefSeq: XM_006530282.3, isoform 1). RNA-seq data and RefSeq genomic annotation suggest that *Tbx5ua* is alternatively spliced, producing several isoforms (Fig. 3a, Additional file 9). In Fig. 3a, we labeled isoforms that were identified in our RNA-seq experiment in at least one stage. Reanalysis of previously published intact/nuclear RNA-seq of cardiomyocytes revealed that *Tbx5ua* is not clearly localized (Fig. 3b) [35]. Previous study reports that quite a few lncRNAs actually exhibit this type of non-localized expression patterns [36].

We first quantified the expression level of the transcript in the heart ventricle, atrium and forelimb during normal development by quantitative RT-PCR (Fig. 3c). We found that the expression level of *Tbx5ua* was increased in the ventricle as development progressed, which was inconsistent with the expression pattern of *Tbx5*. We also examined the expression level of the *Tbx5* isoform that is also transcribed from the bidirectional promoter (Isoform 2, RefSeq: XM_006530280.1). The expression level of that isoform was stable during the entire developmental process, which was also different from the expression pattern of *Tbx5ua* (Additional file 10). Next, we compared the expression level of the lncRNA in both of the ventricles at E11.5 because it is well-known that the expression level of *Tbx5* is higher in the left ventricle than in the right ventricle and that the steep gradient is crucial for establishing a proper ventricular septum [37, 38]. We observed that *Tbx5ua* expression was almost the same between the left and right ventricles at E11.5, while we confirmed the differential expression level of *Tbx5* (Fig. 3d). These results suggest that *Tbx5ua* is not just a byproduct of *Tbx5* and is regulated separately as a different product.

### *Tbx5ua*-knockdown (KD) mice were embryonic lethal with severe abnormalities in the heart

**Fig. 4:**
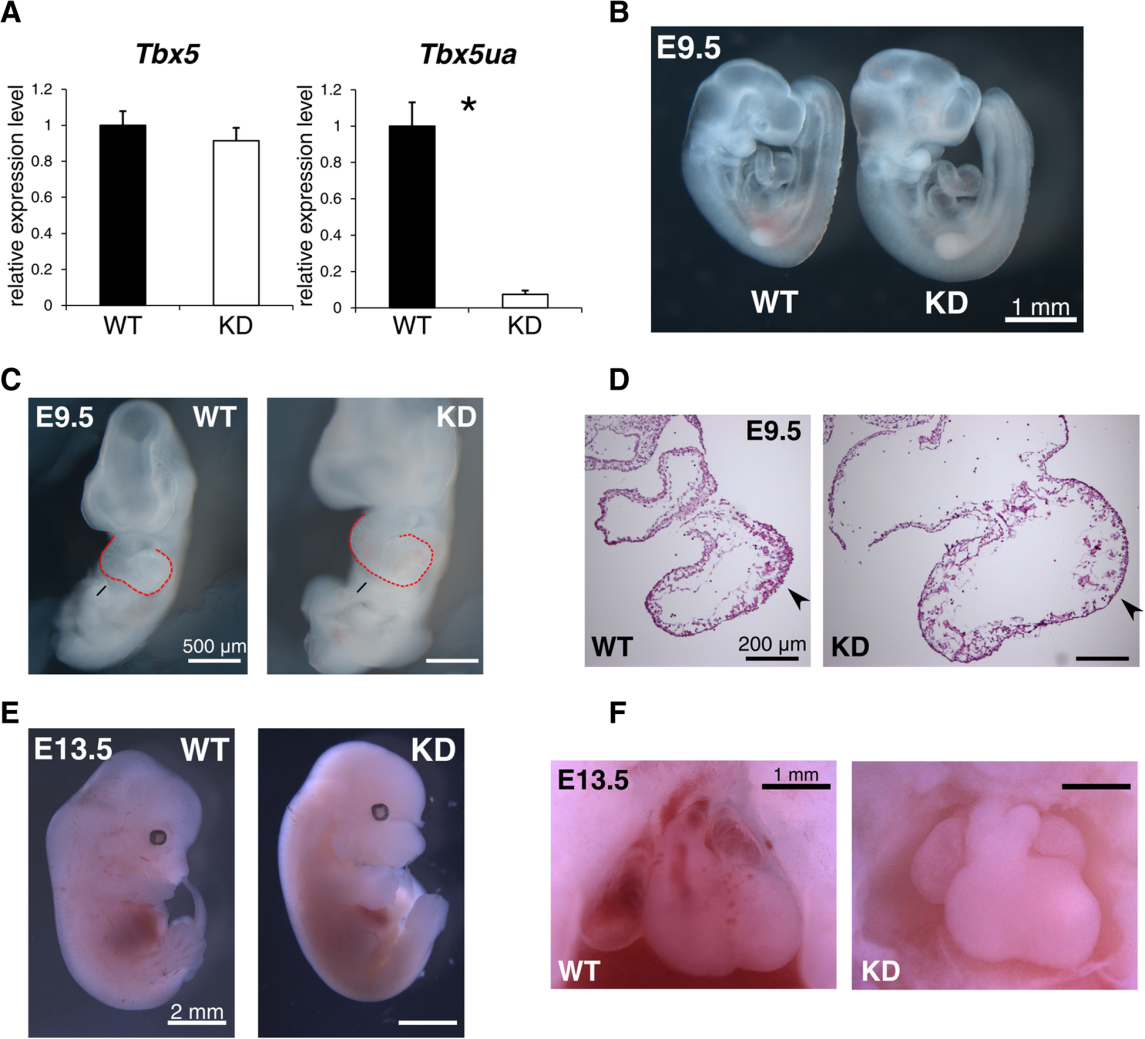
The phenotype of *Tbx5ua* KD chimeric mice. **a** qRT-PCR of *Tbx5ua* and *Tbx5* in E9.5 chimeric mice that were derived from WT and KD ES cells (*n* = 4, * *p* < 0.05, Welch's *t*-test). *Tbx5ua* was successfully knocked down. **b**, **c**, **d** The morphology of E9.5 chimeric embryos. KD embryos show right ventricular hypoplasia (**c**). The ventricular walls of KD embryos appeared abnormally thin (**d**). **e**, **f** The body size and forelimb of KD embryos appeared to be normal in E13.5 chimeric mice, whereas KD embryos showed severe heart defects, including a hypoplastic right ventricle

To determine the function of *Tbx5ua*, we knocked down both alleles of *Tbx5ua* by inserting three tandem copies of bovine growth hormone polyadenylation site (3xpA) at the second exon to prematurely stop transcription in C57BL/6 J-derived ES cells using the CRISPR/Cas9 system (Fig. 3e) [39]. By tetraploid complementation, we obtained completely ES cell-derived mouse embryos from two ES cell lines and their phenotypes were consistent between lines. The expression level of *Tbx5ua* in E9.5 KD mice was strongly repressed to approximately 1/10 of that in control embryos, showing successful knockdown (Fig. 4a). Although the expression levels of *Tbx5* and *Tbx5ua* seemed to be anticorrelated in the heart during development (Fig. 3c), KD of *Tbx5ua* did not result in the increase of *Tbx5* expression level. We also showed that the expression levels of the different *Tbx5* isoforms that are transcribed from all three promoters were not significantly changed (Additional file 11).

Chimeric KD embryos showed right ventricular hypoplasia at E9.5 (Fig. 4b, c, Additional file 12A). Hematoxylin and eosin (HE) staining of the cryosections showed that the ventricular walls of E9.5 KD mice were irregular and lacked trabeculae at some parts in the ventricle (Fig. 4d, Additional files 12B and 13). None of the embryos showed a visible abnormality in the forelimbs, which is observed in *Tbx5*-deficient embryos. By E13.5, all of the KD embryos were dead with a pale body (Fig. 4e). The hearts showed severe ventricular hypoplasia (Fig. 4f), which was probably the cause of the lethality. The forelimbs seemed completely normal even at this stage, which was a significant difference between the phenotype of the *Tbx5ua* KD mice and that of the mouse model of Holt-Oram syndrome (i.e., *Tbx5* heterozygous knockout) (Fig. 4e). The phenotypes among KD embryos were similar and heart-specific, suggesting that they are attributed to genomic modification. In situ hybridization of *Tbx5* revealed normal mRNA expression in the KD ventricle (Additional file 14A). In situ hybridization of *Nppa*, which often shows altered expression pattern in embryos with abnormal morphogenesis, showed an expanded expression around the pre-ventricular septal region of KD embryos (Additional file 14B).

To comprehensively investigate the genes affected by *Tbx5ua* knockdown, we performed RNA-seq with the RNAs extracted from the ventricles of tetraploid chimeric embryos derived from either KD or WT ES cells. We used three embryos for each group and used the Smart-Seq2 protocol to generate libraries from the small amount of RNA. By gene ontology analysis, we found that the genes involved in heart development were significantly enriched among the genes that were determined to be significantly changed (False Discovery Rate; FDR < 0.10, Additional file 15A). However, none of the structural genes that are important for cardiomyocyte contraction were changed (Additional file 15C), suggesting the possibility that *Tbx5ua* has a critical role in morphogenesis rather than in cell differentiation. Finally, we conducted principal component analysis (PCA) on the RNA-seq data (Additional file 15D). The two groups were evidently distinguished only by considering the first principal component.

## Discussion

In this study, we found that many cardiac transcription factor genes have neighboring spliced lncRNAs, especially bidirectional ones. The clear correlation of the expression levels of some bidirectional pairs suggests their regulatory roles. A typical example of such lncRNAs is *Upperhand*, which is divergent to *Hand2* [18]. The transcription of *Upperhand* but not the mature transcripts were shown to be necessary for the transcription of *Hand2* by altering the local epigenetic environment. Many lncRNAs are known to regulate local transcription through the recruitment of epigenetic-altering protein complexes. Since the expression level of transcription factors is generally low and many of them are haploinsufficient, even a relatively small fluctuation could lead to severe consequences. It is possible that divergent lncRNAs are enriched among dose-sensitive genes to stabilize the expression level of adjacent genes.

An alternative hypothesis is that these transcription factors could be setting up optimal transcriptional environments for lncRNAs to evolve. As some transcription factor genes can cause direct lineage reprogramming, they are thought to define cell types. Thus, the use of these preexisting transcriptional environments is a cost-efficient way to evolve cell type-specific lncRNAs. Some studies have demonstrated that bidirectional transcription is a general phenomenon and that a so-called transcription ripple effect exists [40, 41]. These findings also support our idea by showing that the preexisting transcriptional environment enables precursor transcripts to evolve into defined, functional ones. In summary, active transcription factor genes may have been good sources from which lncRNA genes could evolve due to the cell lineage-specific and active epigenetic environment.

We showed that *Tbx5ua* is conserved from mammals to birds. Comparison of the sequence of *Tbx5ua* between mouse and chicken showed less similarity, but it does not mean that the function is not conserved as the previous studies have shown that precise conservation at the sequence level is not necessarily required for the functional conservation of lncRNAs [42, 43]. *Tbx5ua* was not found in the NCBI genomic annotations of reptiles, amphibians or fish at the corresponding loci. In fact, by conducting the reanalysis on the publicly available RNA-seq data, including RNA-seq of the adult heart of chicken, anole and frog (GSE41338) [44], we could confirm that *Tbx5ua* is expressed only in chicken among these species at the adult stages (Additional file 16). It is interesting that *Tbx5ua* is conserved in two-ventricle animals, which possess a complete ventricular septum, but not in animals with non-septated hearts. There is a possibility that the acquisition of *Tbx5ua* might have contributed to the evolution of a complete ventricular septum.

We showed that *Tbx5ua* lncRNA is required for proper heart development. Since we knocked down *Tbx5ua* by prematurely terminating the transcription, the loading of transcription complex at the transcription start site is not inhibited. Thus, if the transcription of *Tbx5ua* itself is important for altering the local transcriptional environment, our KD scheme is not sufficient to assess the true function of *Tbx5ua*. Although preliminary, our data suggested that the expression pattern of Tbx5 protein is altered in the KD mice (Additional file 17). While we do not have any evidences supporting the direct roles of *Tbx5ua* on *Tbx5*, the function of *Tbx5ua* might be atypical for a divergent lncRNA since many of such lncRNAs like *Upperhand* are shown to alter the transcription of neighboring genes. How the left-sided expression of *Tbx5* is regulated is an unsolved important issue to understand the molecular mechanism of heart development [45].

## Conclusions

This study revealed that many genes involved in the heart development, particularly transcription factor genes, are associated with spliced lncRNAs that are derived from nearby genomic regions. Furthermore, many of these lncRNAs were divergently transcribed from the promoter of protein-coding genes. We find that bidirectional lncRNAs are enriched among haploinsufficient genes, suggesting that they have functional roles for the regulation of dose-sensitive genes.

## Methods

### RNA-seq

Total RNAs from embryonic and adult mice were extracted with Sepasol-RNA I Super G (Nacalai #09379–55). The cDNA libraries for paired-end RNA-seq for the screening of lncRNAs were prepared from 1 μg of RNAs with Truseq Stranded Total RNA Library Prep Kit (Illumina #RS-122-2201) according to Illumina’s instructions. The cDNA libraries for tetraploid chimeric mice were prepared by Smart-Seq2 protocol according to the original paper [46] with 12 cycles of preamplification and 9 cycles of enrichment PCR.

### qRT-PCR

Total RNAs were extracted with Sepasol-RNA I Super G (Nacalai #09379–55). cDNA samples were prepared using RevaTra Ace qPCR RT Master Mix with gDNA remover (Toyobo #FSQ-301). Real-time PCR was performed with SYBR Premix EX Taq II (Takara #RR820). The PCR conditions were as follows: 95 °C for 30 s followed by 50 cycles of 95 °C for 5 s and 60 °C for 30 s, and a subsequent dissociation curve measurement. We used *Gapdh* as an internal control. Gene-specific primers are listed in the Additional file 18: Materials and Methods.

### Mice

C57BL/6 J mice were purchased from CLEA Japan. For the first round of chimeric mice generation, we used mice form CLEA Japan. For the second experiment, we used tetraploid embryos from Ark Resource and recipient mice from Sankyo Labo Service. Mice are sacrificed by cervical dislocation.

### Generation of genome edited ES cells

ES cells were cultured on the MEF feeder in ES culture medium (i.e., Knockout DMEM (Gibco #10829018), 20% Knockout Serum Replacement (Gibco #10828028), 1 * GlutaMAX (Gibco #10566016), 1 * NEAA (Sigma #M7145), 1 mM sodium pyruvate (Gibco # 11360070), 10^− 4^ M 2-Mercaptoethanol, 1000 U/ml LiF (Wako #198–15,781)).

The guide RNA (gRNA) target sequence to induce double strand break is 5’-GTCACTGCCGCTCCAATCCTCGG-3′. We designed the gRNA with Cas-OFFinder (http://www.rgenome.net/cas-offinder/), so that the number of off-target sites was as few as possible. Our gRNA has no potential off-target sites with 0, 1 or 2 mismatches and just 4 potential off-targets with 3 mismatches and proper PAM sequence, of them none is exonic. Homology directed repair donors were constructed so that the *Neo*^*R*^ or *EGFP* expressing cassette was flanked by ~ 1,000 bp 5′ and 3′ homologous arms cloned from genomic DNA. ES cells were transfected with Cas9 expressing plasmid, gRNA expressing plasmid and the donor plasmid along with non-gRNA expressing negative control. After two days, the ES cells were passaged onto SNL feeder cells and cultured for 8 days with 250 μg/ml G418 and surviving EGFP-positive colonies were manually picked up. After one more cycle of single colony picking up to ensure that the ES cells are clonal, they were subjected to cell permeable Cre treatment [47] to remove the selection cassettes, and then EGFP-negative colonies were picked up to obtain cells without selection cassettes. Finally, the ES cells from each colony were genotyped and karyotyped.

### Generation of tetraploid chimeric mice

Generation of chimeric mice was performed as described previously [39].

### Histology

Immunohistochemistry for Tbx5 were performed as follows. Antigen retrieval was performed by microwaving the sections in 10 mM citrate acid pH 6.0. Then they were permeabilized for 10 min in 0.2% Triton X in PBS at RT. Blocking was performed with 10% Blocking One (Nacalai #03953–95) in PBST. Tbx5 antibody (Santa Cruz Biotechnology #sc-17,866) was diluted 1/100 in 5% Blocking One/PBT and second antibody (Invitrogen #A-11037) was diluted 1/200.

In situ hybridization was performed as follows. First, cryosections were permeabilized in 0.2 N HCl for 15 min. After washing with PBT three times, the sections were re-fixed with freshly made 4% PFA for 15 min. After washing, the sections were hybridized with DIG labeled probes at 70 °C for ON. The next day, the sections were washed with 0.2× SSC three times. After blocking the sections with 10% sheep serum for 1 h, 1/1000 diluted anti-DIG-AP Fab fragment (Roche #11093274910) were added and incubated for an hour at RT. After washing with TBST, the sections were washed with NTMT and colored with BM purple.

## Additional files

Additional file 1: We counted the exon numbers of reconstructed transcripts and compared them with known exon numbers. The exon numbers were determined based on the maximum of alternative transcripts for each gene. We only took into account genes with their exon number 12 or less since the exon numbers of more than 98.5% of known lncRNAs expressed in the heart fall under the category. The relation between exon number differenced and fpkm was fitted with an exponential curve. This result demonstrates that 1.0 fpkm is sufficient to infer gene models in our RNA-seq experiment. (PNG 127 kb)

Additional file 2: **Table S1.** List of spliced lncRNA candidates that were identified in this study. (PDF 204 kb)

Additional file 3: **Table S2.** List of antisense lncRNA candidates and their corresponding protein-coding genes. (PDF 17 kb)

Additional file 4: **Table S3.** List of bidirectional lncRNA candidates and their corresponding protein-coding genes. (PDF 21 kb)

Additional file 5: Conserved bidirectional lncRNAs in human USCS genome annotation are shown. We found that many of the mouse lncRNAs divergent to important cardiac transcription factor genes have conserved transcripts at the corresponding loci in human genome. (PNG 733 kb)

Additional file 6: **Table S4.** List of bidirectional lncRNA candidates that were identified from the analysis of the NCBI RefSeq database (GRCm38.p3). (PDF 148 kb)

Additional file 7: The proportion of housekeeping genes among all genes and among haploinsufficient genes was calculated and it was not found to be significantly correlated. This result eliminates the possibility that the enrichment of genes with bidirectional lncRNAs among haploinsufficient genes is due to the pseudo-correlation generated through housekeeping-haploinsufficient correlation. (PNG 53 kb)

Additional file 8: The alignment of mouse *Tbx5ua* (isoform 2) and its human homolog (RefSeq: NR_038440.1) produced by EMBOSS water. The sequence shown in red is highly conserved as determined by EMBOSS Matcher. The sequences are highly conserved at the 5′ side. (PDF 158 kb)

Additional file 9: The sequence of each isoform of *Tbx5ua* as determined by cufflinks. Isoform numbers correspond to those in Fig. 3a. (PDF 46 kb)

Additional file 10: qRT-PCR analysis of a *Tbx5* isoform that is transcribed from the promoter that also produces *Tbx5ua* (isoform 2)*.* The expression pattern of this isoform over development is also inconsistent with that of *Tbx5ua*, indicating that they are post-transcriptionally modulated or the directional of transcription is somehow controlled. (PNG 50 kb)

Additional file 11: qRT-PCR analysis of KD and WT mouse ventricles for all the *Tbx5* isoforms detected in our RNA-seq analysis. The expression levels are not significantly changed for all the isoforms. The isoform numbers are indicated in Fig. 3a. (PNG 72 kb)

Additional file 12: Morphological phenotype of *Tbx5ua* KD embryos derived from another ESC line is shown and is consistent with our first ESC line. (PNG 2047 kb)

Additional file 13: The thickness of the ventricular wall around the interventricular zone was measured for WT and KD embryos and KD embryos tended to have thinner wall. (B) (PNG 44 kb)

Additional file 14: In situ hybridization of *Tbx5* and *Nppa* in WT and KD chimeric mice at E9.5. The expression pattern of *Tbx5* at the mRNA level appeared to be not changed. KD embryos showed an ectopic expression of *Nppa* around the pre-ventricular septal region, which is frequently observed among embryos with abnormal development of ventricular septum. (PNG 6143 kb)

Additional file 15: RNA-seq analysis of WT and KD chimeric embryos at E9.5 (*n* = 3). (A) Genes related to heart development were enriched among genes that were changed significantly. (B) Structural protein genes were not changed, suggesting that the KD did not affect the differentiation of cardiomyocytes in a major way. (C) The scatter plot of log2-transformed expression levels shows that the expression pattern of KD embryos did not change drastically. (D) Principal component analysis on the RNA-seq analysis. WT and KD mice are distinguishable only by the first component. (PNG 767 kb)

Additional file 16: Reanalysis of RNA-seq data from chicken, anole and zebrafish. *Tbx5ua* is conserved only in chicken, which possesses a complete ventricular septum, among these species. (PNG 107 kb)

Additional file 17: Tbx5 IHC of WT and KD embryos were quantified. The ventricle was divided into three regions and the staining intensity in each nuclear was measured using ImageJ. Nuclear binary masks were produced from DAPI staining. Note that we only quantified cells that are not in the outermost layer of the ventricle because speckle-like background was observed in the region. Mann-Whitney U test was performed for each sample with multiple testing correction with Holm method (*: *p* < 0.05). Tbx5 expression is vanished in the interventricular zone and diminished in the left ventricle in KD embryos. (PNG 1201 kb)

Additional file 18: Materials and Methods. List of qPCR primers used in this study. (XLSX 11 kb)

## Abbreviations

CHD: congenital heart disease
fpkm: fragments per kilobase of exon per million mapped fragments
gRNA: guide RNA
KD: knockdown
lncRNA: long non-coding RNA
*Tbx5ua*: *Tbx5 upstream antisense product*
TSS: transcription start site
WT: wildtype
