# Supplementary figures and images for: Important cardiac transcription factor genes are accompanied by bidirectional long non-coding RNAs
Source: BMC Genomics. 2018 Dec 27;19:967. doi: 10.1186/s12864-018-5233-5 (PMC6307297; doi:10.1186/s12864-018-5233-5)

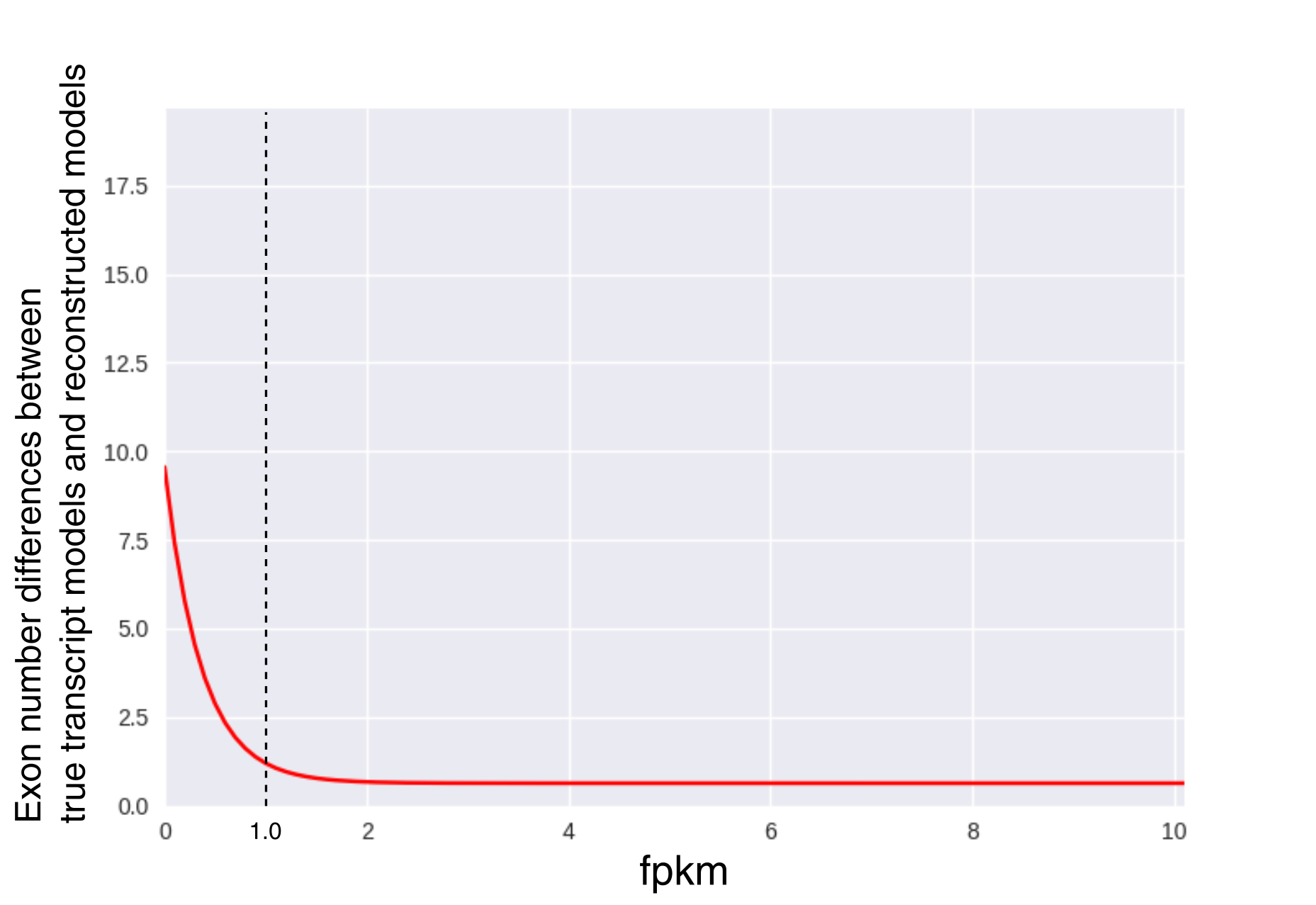

Supplement: Supplementary file 1 — We counted the exon numbers of reconstructed transcripts and compared them with known exon numbers. The exon numbers were determined based on the maximum of alternative transcripts for each gene. We only took into account genes with their exon number 12 or less since the exon numbers of more than 98.5% of known lncRNAs expressed in the heart fall under the category. The relation between exon number differenced and fpkm was fitted with an exponential curve. This result demonstrates that 1.0 fpkm is sufficient to infer gene models in our RNA-seq experiment. (PNG 127 kb) [file 12864_2018_5233_MOESM1_ESM.png]

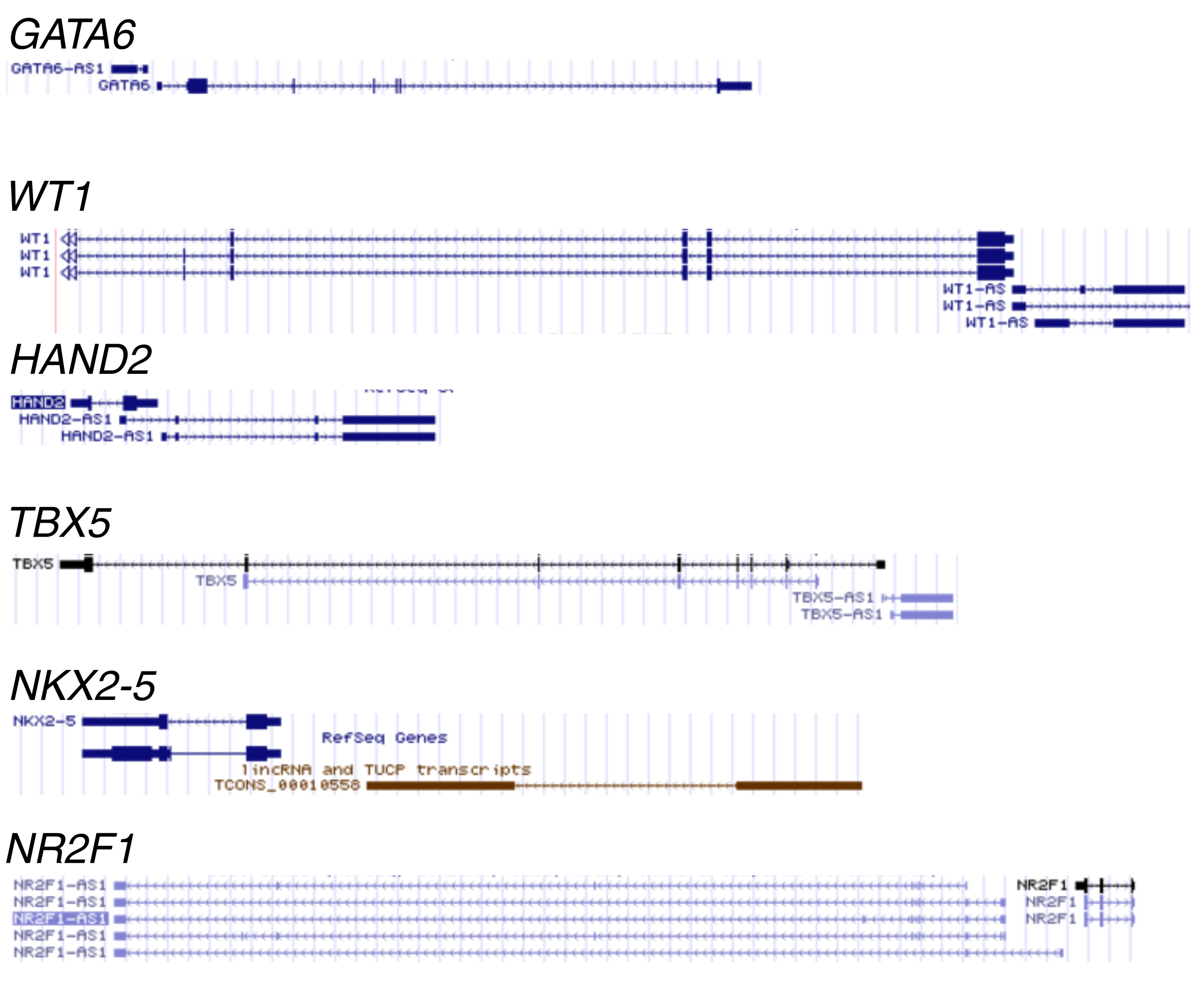

Supplement: Supplementary file 5 — Conserved bidirectional lncRNAs in human USCS genome annotation are shown. We found that many of the mouse lncRNAs divergent to important cardiac transcription factor genes have conserved transcripts at the corresponding loci in human genome. (PNG 733 kb) [file 12864_2018_5233_MOESM5_ESM.png]

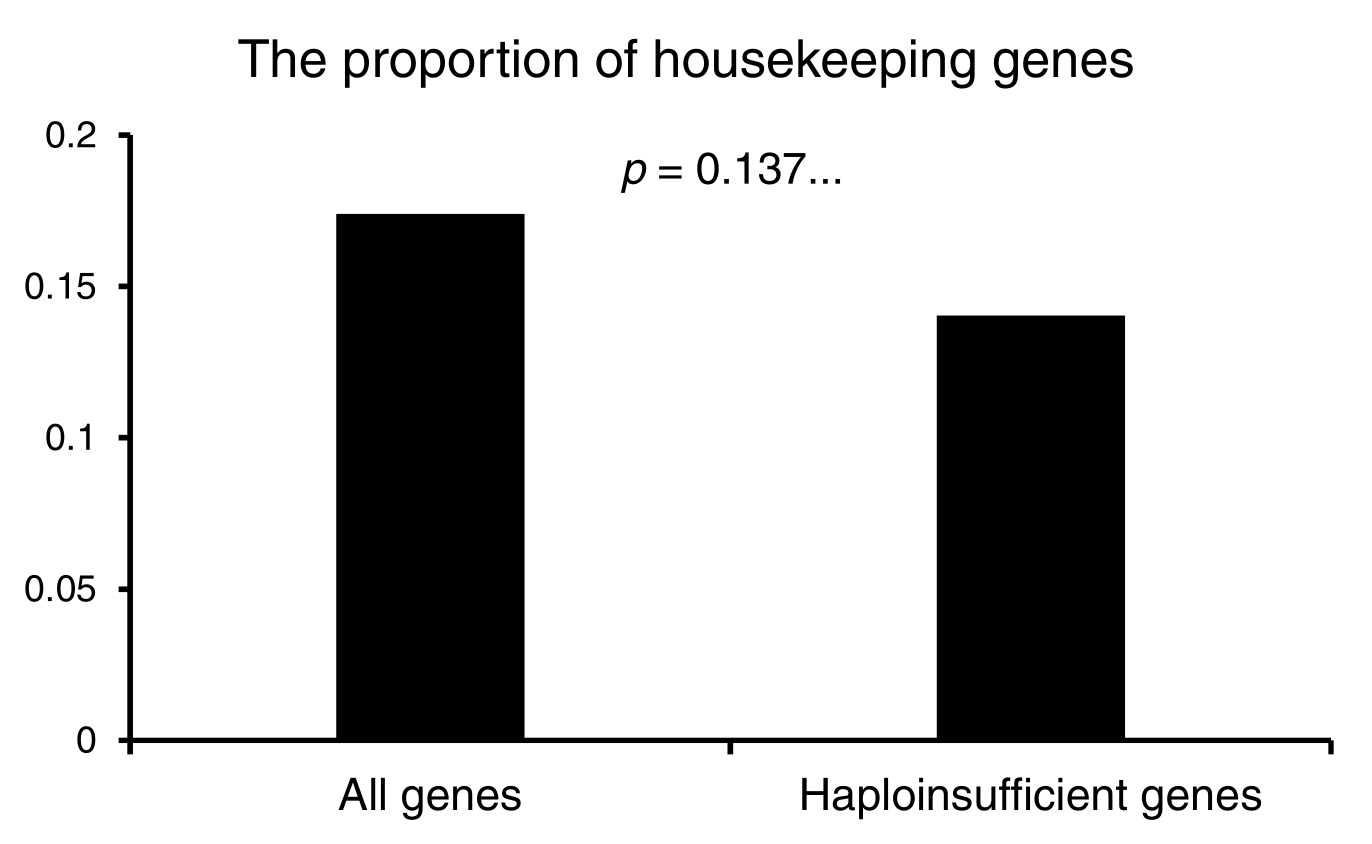

Supplement: Supplementary file 7 — The proportion of housekeeping genes among all genes and among haploinsufficient genes was calculated and it was not found to be significantly correlated. This result eliminates the possibility that the enrichment of genes with bidirectional lncRNAs among haploinsufficient genes is due to the pseudo-correlation generated through housekeeping-haploinsufficient correlation. (PNG 53 kb) [file 12864_2018_5233_MOESM7_ESM.png]

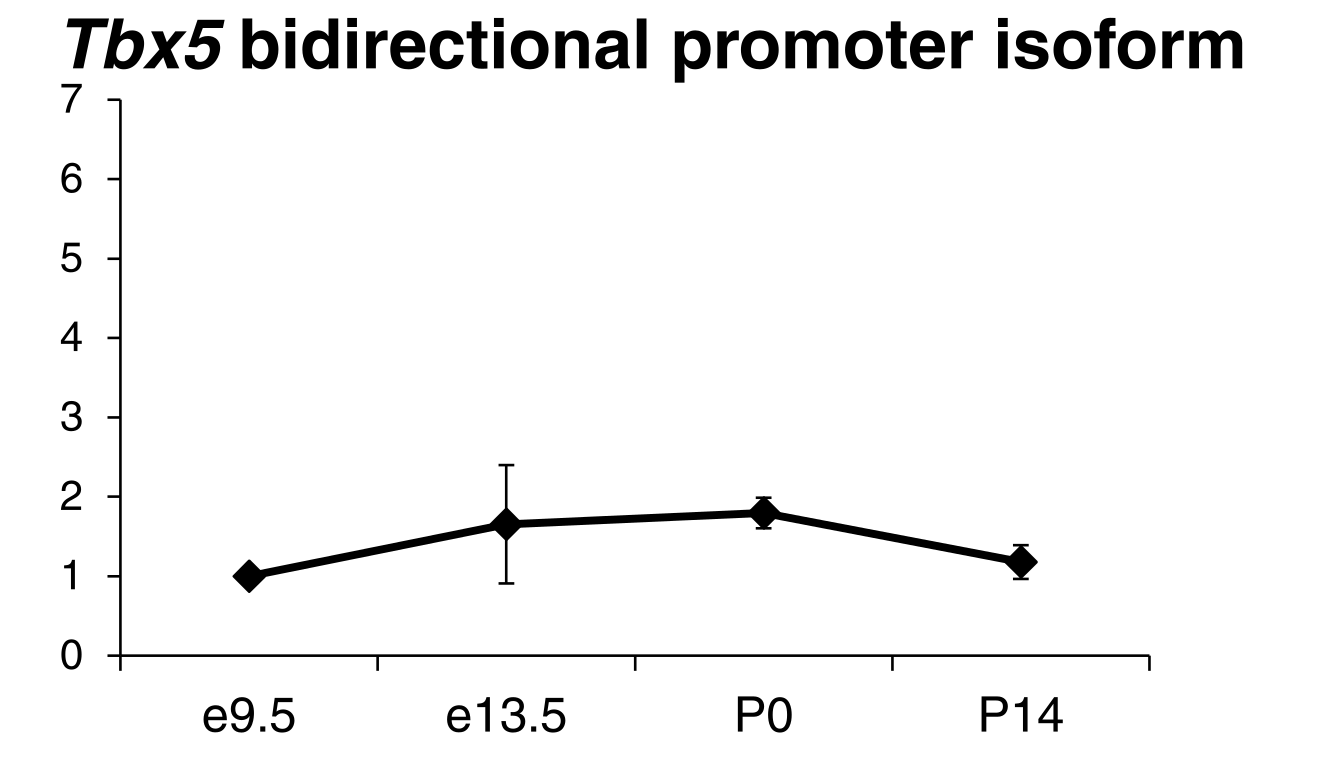

Supplement: Supplementary file 10 — qRT-PCR analysis of a Tbx5 isoform that is transcribed from the promoter that also produces Tbx5ua (isoform 2). The expression pattern of this isoform over development is also inconsistent with that of Tbx5ua, indicating that they are post-transcriptionally modulated or the directional of transcription is somehow controlled. (PNG 50 kb) [file 12864_2018_5233_MOESM10_ESM.png]

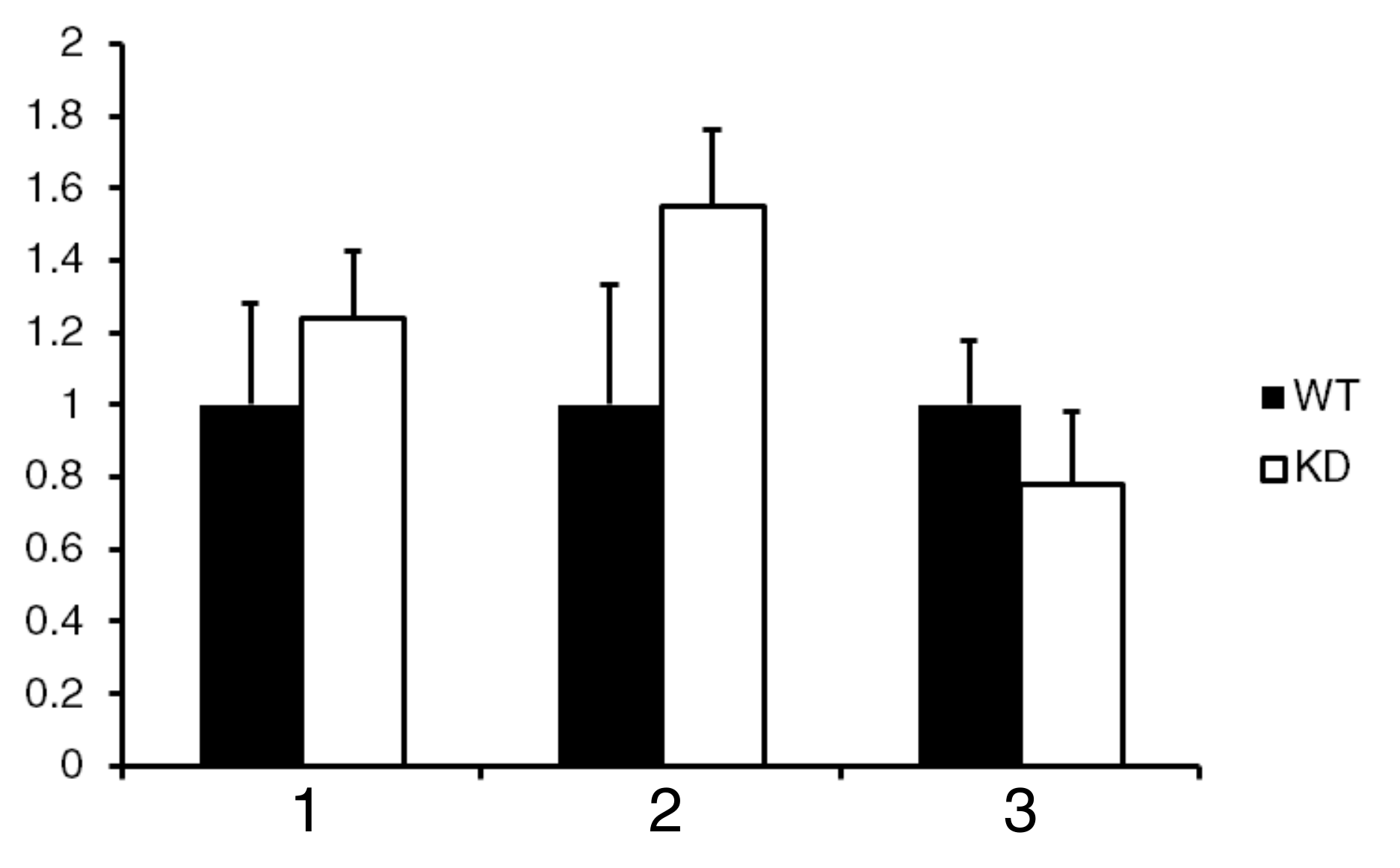

Supplement: Supplementary file 11 — qRT-PCR analysis of KD and WT mouse ventricles for all the Tbx5 isoforms detected in our RNA-seq analysis. The expression levels are not significantly changed for all the isoforms. The isoform numbers are indicated in Fig. 3a. (PNG 72 kb) [file 12864_2018_5233_MOESM11_ESM.png]

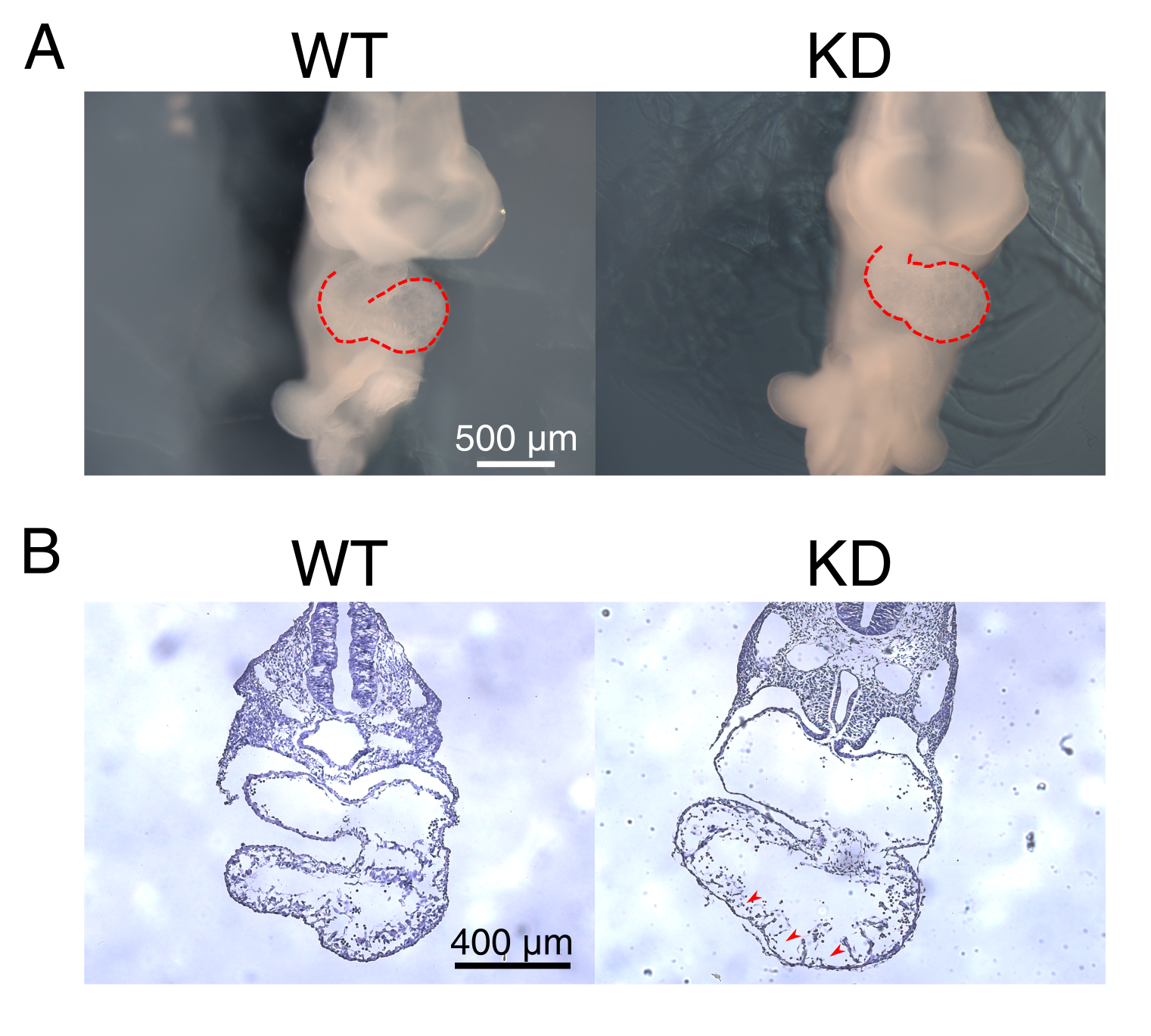

Supplement: Supplementary file 12 — Morphological phenotype of Tbx5ua KD embryos derived from another ESC line is shown and is consistent with our first ESC line. (PNG 2047 kb) [file 12864_2018_5233_MOESM12_ESM.png]

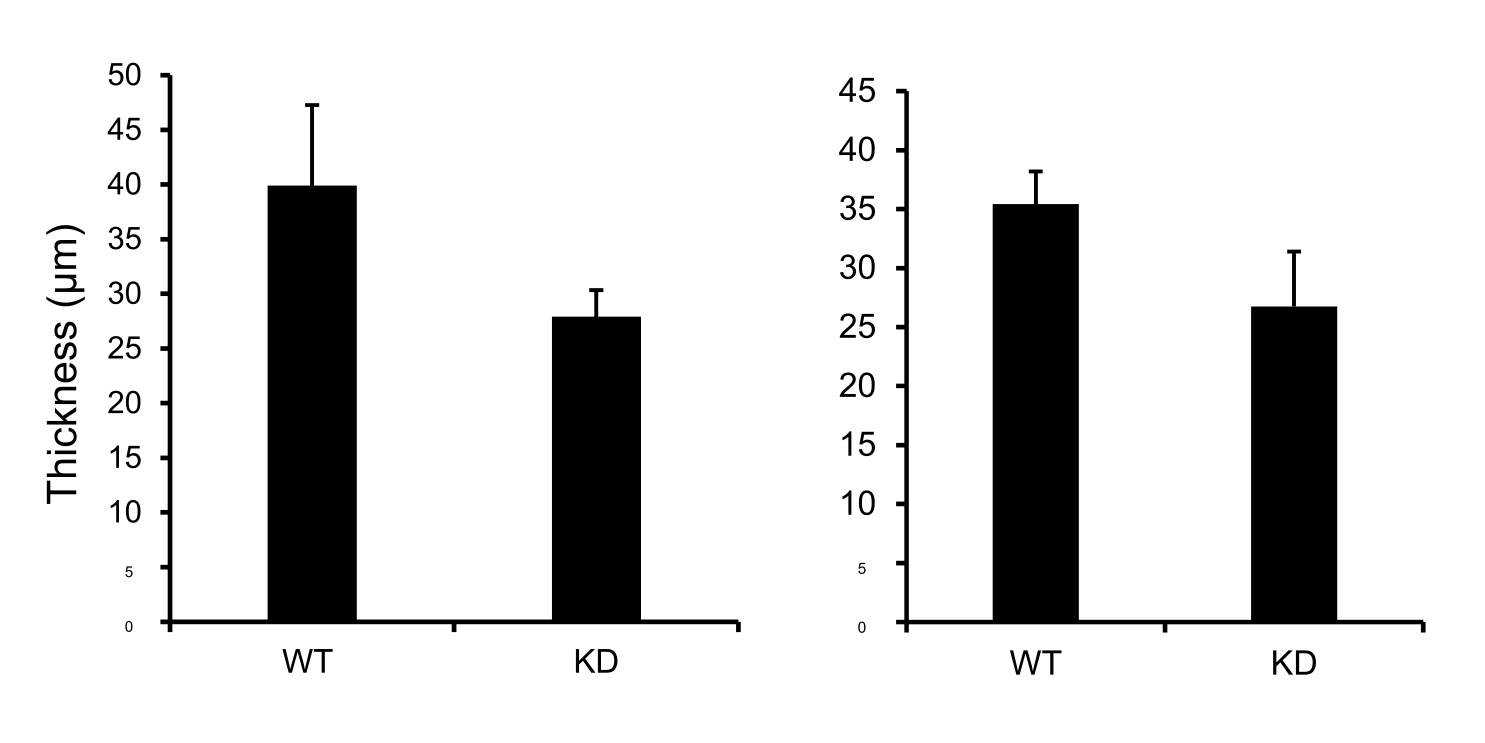

Supplement: Supplementary file 13 — The thickness of the ventricular wall around the interventricular zone was measured for WT and KD embryos and KD embryos tended to have thinner wall. (B) (PNG 44 kb) [file 12864_2018_5233_MOESM13_ESM.png]

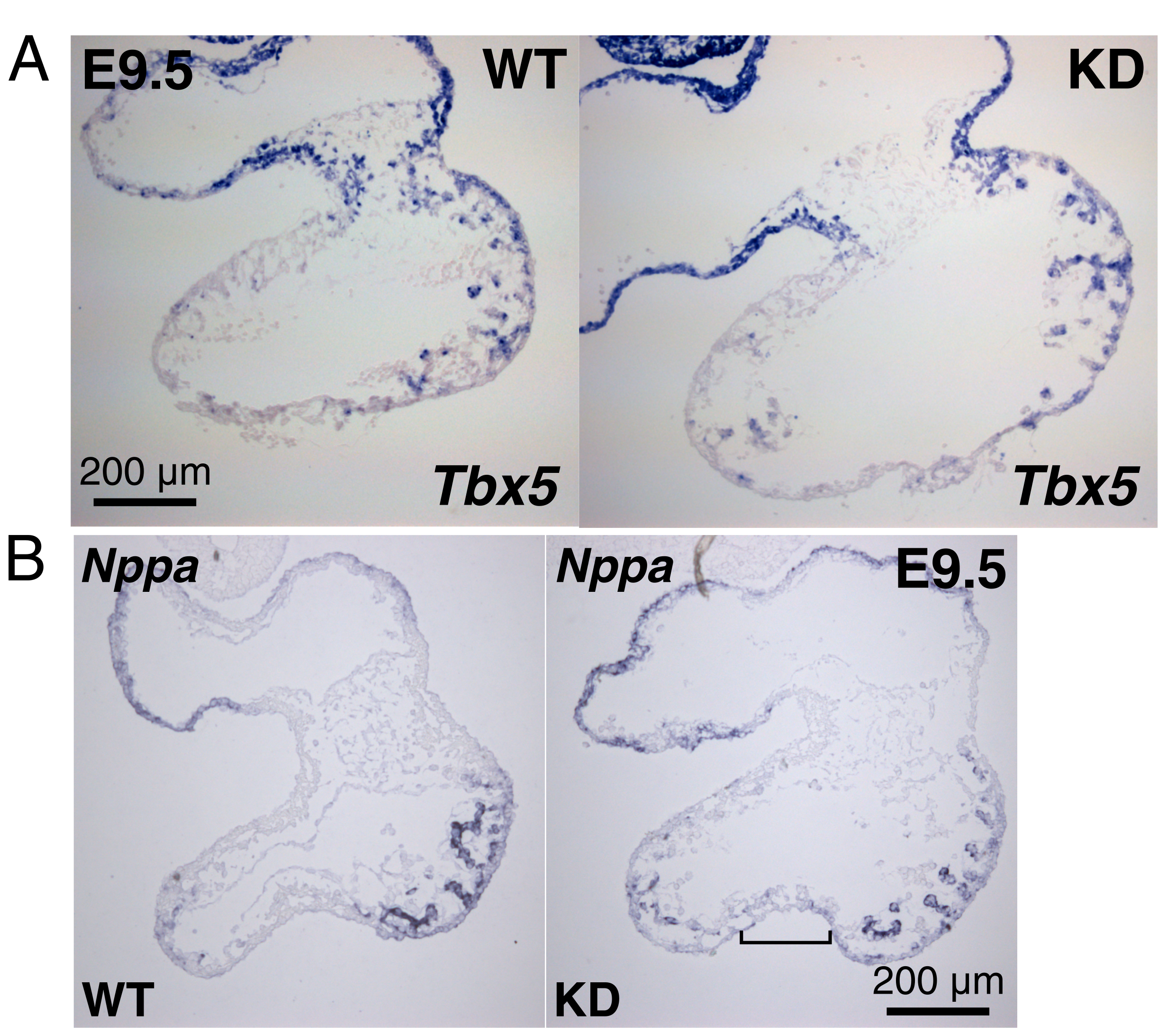

Supplement: Supplementary file 14 — In situ hybridization of Tbx5 and Nppa in WT and KD chimeric mice at E9.5. The expression pattern of Tbx5 at the mRNA level appeared to be not changed. KD embryos showed an ectopic expression of Nppa around the pre-ventricular septal region, which is frequently observed among embryos with abnormal development of ventricular septum. (PNG 6143 kb) [file 12864_2018_5233_MOESM14_ESM.png]

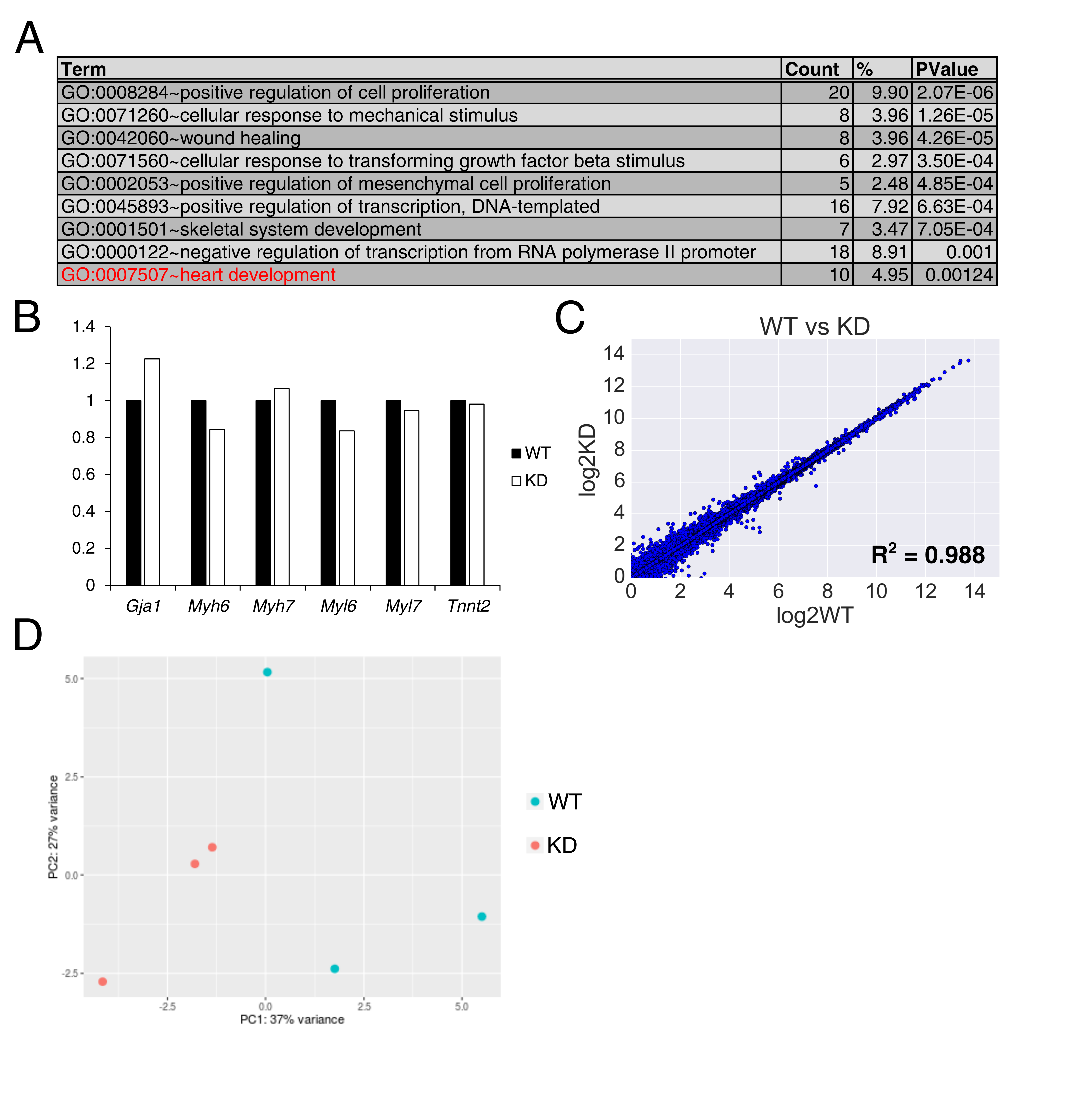

Supplement: Supplementary file 15 — RNA-seq analysis of WT and KD chimeric embryos at E9.5 (n = 3). (A) Genes related to heart development were enriched among genes that were changed significantly. (B) Structural protein genes were not changed, suggesting that the KD did not affect the differentiation of cardiomyocytes in a major way. (C) The scatter plot of log2-transformed expression levels shows that the expression pattern of KD embryos did not change drastically. (D) Principal component analysis on the RNA-seq analysis. WT and KD mice are distinguishable only by the first component. (PNG 767 kb) [file 12864_2018_5233_MOESM15_ESM.png]

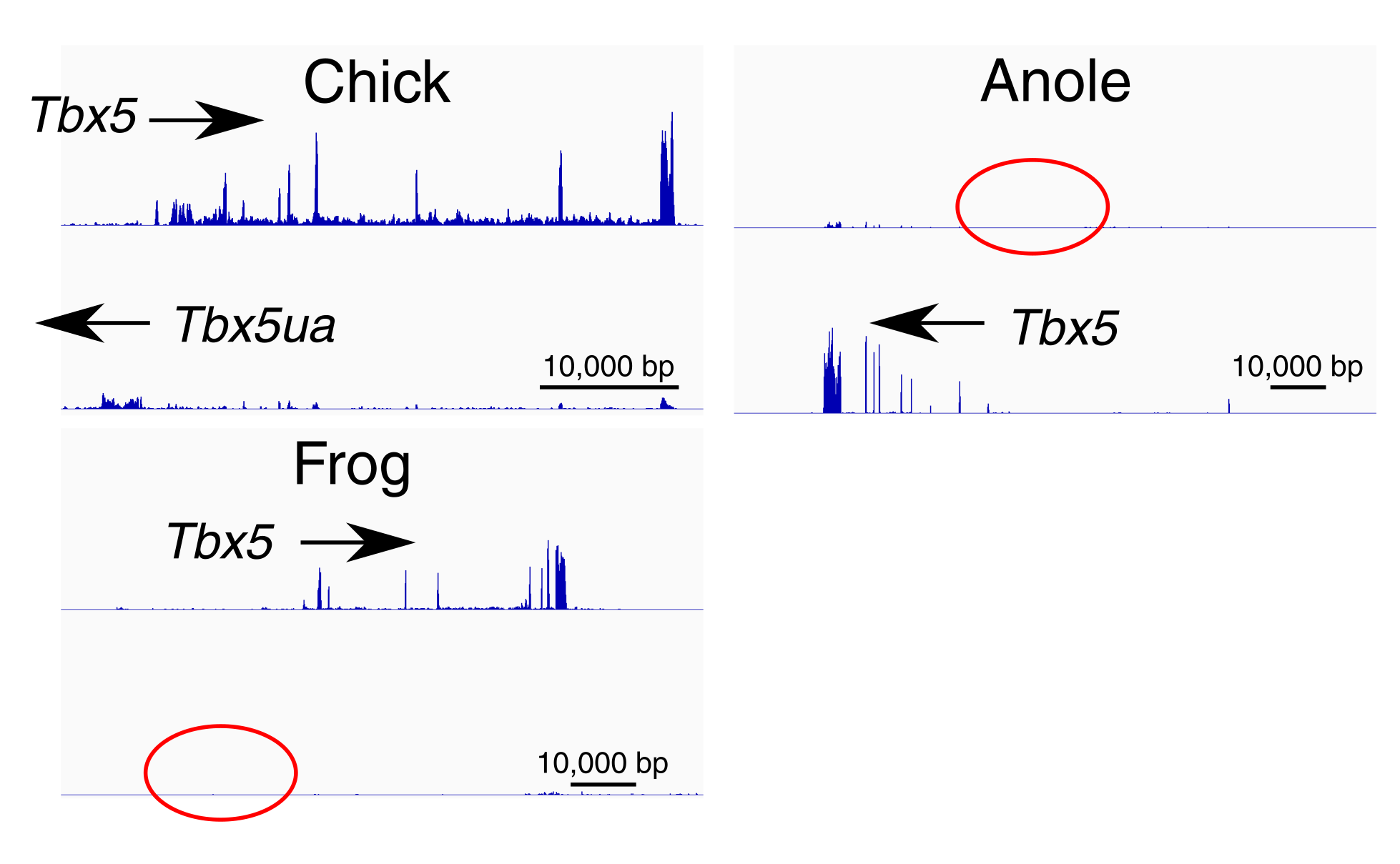

Supplement: Supplementary file 16 — Reanalysis of RNA-seq data from chicken, anole and zebrafish. Tbx5ua is conserved only in chicken, which possesses a complete ventricular septum, among these species. (PNG 107 kb) [file 12864_2018_5233_MOESM16_ESM.png]

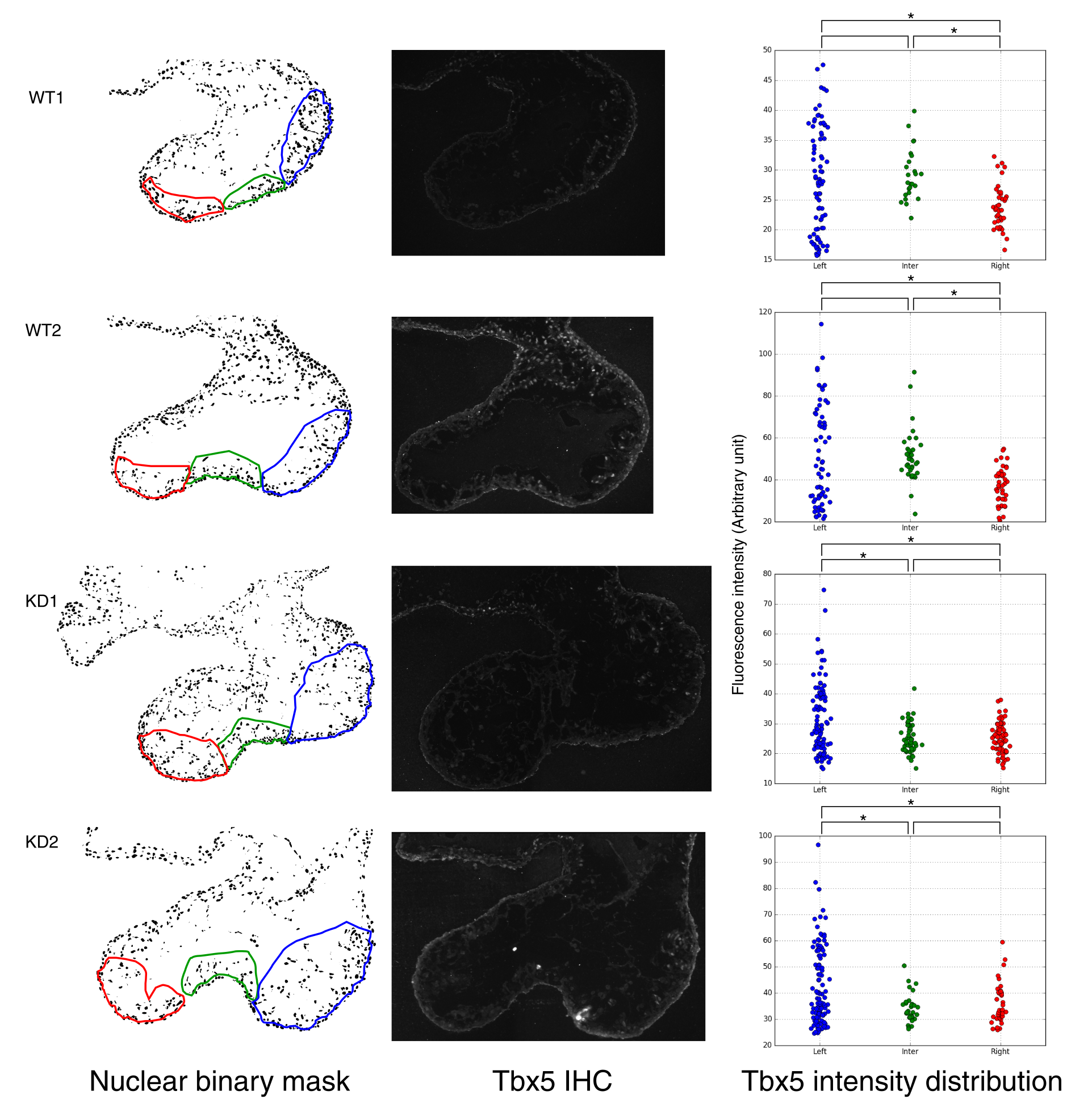

Supplement: Supplementary file 17 — Tbx5 IHC of WT and KD embryos were quantified. The ventricle was divided into three regions and the staining intensity in each nuclear was measured using ImageJ. Nuclear binary masks were produced from DAPI staining. Note that we only quantified cells that are not in the outermost layer of the ventricle because speckle-like background was observed in the region. Mann-Whitney U test was performed for each sample with multiple testing correction with Holm method (*: p < 0.05). Tbx5 expression is vanished in the interventricular zone and diminished in the left ventricle in KD embryos. (PNG 1201 kb) [file 12864_2018_5233_MOESM17_ESM.png]
