# Supplementary material for: Important cardiac transcription factor genes are accompanied by bidirectional long non-coding RNAs
Source: BMC Genomics. 2018 Dec 27;19:967. doi: 10.1186/s12864-018-5233-5 (PMC6307297; doi:10.1186/s12864-018-5233-5)
Supplement: Supplementary file 2 — Table S1. List of spliced lncRNA candidates that were identified in this study. (PDF 204 kb) [file 12864_2018_5233_MOESM2_ESM.pdf]

					Expression level (fpkm)					
Gene name	coordinate	strand	the nearest gene (distance)	Heart selective	E10.5	E13.5	Adult	Brain	Liver	Kidney
Lnc1	chr1:7177921-7178377	+	Pcmtd1 (3528)	no	9.5914	32.4348	5.2151	25.0088	0	0
Lnc2	chr1:7381223-7397914	-	Pcmtd1 (223521)	no	2.138	0.4707	0.321	0.3634	0.339547	0.0890527
Snhg6	chr1:9942024-9944118	-	6030422M02Rik (3164)	no	23.9141	62.0811	25.3066	82.0509	9.80333	128.086
Lnc3	chr1:43210976-43222788	+	Fhl2 (12966)	yes	0.0558	0.2896	2.4265	0	0	0
Lnc4	chr1:45857176-45857843	-	Wdr75 (34230)	no	116.086	144.456	14.7583	61.9457	16.8532	8.18507
1110034B05Rik	chr1:57388031-57406674	-	1110034B05Rik (0)	no	9.9091	10.4103	5.9286	5.8682	14.5733	19.3613
Lnc5	chr1:60811047-60816001	+	Cd28 (37018)	yes	0.0842	0.047	3.0397	0	0	0.0157033
2810408I11Rik	chr1:64679868-64690659	-	Ccnyl1 (685)	no	8.1168	7.4365	0.1433	1.3943	0.572877	17.549
Apol7d	chr1:71653331-71662843	+	Fn1 (81)	yes	1.0752	0.5899	0.1822	0.1801	0	0.346691
Lnc6	chr1:71692737-71711384	+	Fn1 (39487)	yes	0	1.1139	0.2714	0.1562	0	0
Lnc7	chr1:72941694-72951677	+	Igfbp5 (66719)	yes	0.1125	0.02	2.8596	0.0219	0.168397	0
Lnc8	chr1:85072755-85075657	-	C130026I21Rik (12481)	yes	0.1999	0.195	1.1593	0	0	0
Lnc9	chr1:92379661-92383296	+	Ndufa10 (60057)	yes	0.0335	0.0444	1.2032	0	0	0
Lnc10	chr1:93911243-93918184	+	D2hgdh (0)	yes	2.087	0.1199	8.2892	0.021	2.59565	0
B230216N24Rik	chr1:98029217-98047793	-	Pam (0)	no	3.3236	0.7984	1.922	9.9109	1.78005	0.204221
Lnc11	chr1:107938962-107950346	+	Serpinb8 (329979)	yes	0.1983	0.1831	5.1302	0.0215	0	0
Lnc12	chr1:107962315-107978815	+	Serpinb8 (353332)	yes	0	0	3.2849	0	0	0
Lnc13	chr1:107978895-108031102	+	Serpinb8 (369912)	yes	0.0216	0.0566	2.3398	0	0	0
Lnc14	chr1:108056243-108059839	+	Serpinb8 (447260)	yes	0	0.1779	3.5042	0	0	0
2900060B14Rik	chr1:118458668-118459265	-	Clasp1 (0)	no	21.6373	13.7146	13.8404	0.3474	0	0.964847
Lnc15	chr1:119529504-119535618	-	Tmem185b (6635)	no	0.9245	1.3	0.1349	0.2973	0.151505	0.0601027
Lnc16	chr1:120762775-120763803	+	En1 (154784)	yes	0	0.0925	1.5183	0	0	0
2900009J06Rik	chr1:127753616-127774054	-	Acmsd (0)	no	2.1272	1.459	0.5834	3.4341	8.02473	0.761099
Gm19461	chr1:133249851-133269808	-	Plekha6 (0)	yes	0.0624	0.0253	1.8609	0.1548	2.02822	0.0575045
Lnc17	chr1:137898601-137947793	+	Ptprc (164259)	no	15.3133	11.0012	3.9924	7.2381	0.159535	0.608615
Lnc18	chr1:151338660-151344481	-	Ivns1abp (16)	no	1.7407	0.9879	4.3292	0.7573	2.12715	0.421346
Lnc19	chr1:155211505-155244315	-	Xpr1 (31093)	no	4.0161	5.3014	7.9307	2.1015	23.8193	0.313084
2810025M15Rik	chr1:157412351-157420236	+	Rasal2 (0)	no	60.0114	54.6647	19.5107	26.4312	11.2963	17.0305
Gas5	chr1:161034945-161040287	+	Zbtb37 (83)	no	49.5182	38.6919	22.0894	58.0096	11.0809	301.925
Lnc20	chr1:161725745-161727680	-	Fasl (53011)	no	10.4591	13.537	5.7177	34.1489	0.0956919	0
Dnm3os	chr1:162217120-162227053	+	Dnm3 (0)	no	2.1923	12.5076	2.1597	1.0057	0.133534	1.23137
Lnc21	chr1:165108717-165115461	-	Tbx19 (22391)	yes	3.8459	0.7999	0	0	0	0
Lnc22	chr1:173795204-173796935	-	Mndal (0)	yes	0.3525	0.1159	6.3377	0	0	0
Lnc23	chr1:173797969-173802655	-	Mndal (0)	yes	0.5942	0.1145	19.6596	0	0	0
Lnc24	chr1:178309589-178318544	-	Fam36a (608)	no	2.5997	1.0546	2.667	1.3756	0.480811	0.787767
Lnc25	chr1:179705404-179709512	-	Sccpdh (22328)	yes	0.098	0.1096	1.0035	0	0	0
Lnc26	chr1:186587128-186615340	+	Tgfb2 (35687)	yes	2.4986	4.198	9.0391	0	0	0
Lnc27	chr1:186749414-186752466	+	Rrp15 (56)	no	0.1987	1.8691	0.2933	3.0186	0.467372	0.915597
Gm10516	chr1:192136897-192151336	+	Rcor3 (0)	no	0.571	1.0704	3.6078	2.1637	2.31082	1.64105
Lnc28	chr1:193273287-193281188	+	G0s2 (87)	yes	0.2647	0.6187	3.6909	0.0896	0.466348	0.757132
A330023F24Rik	chr1:195017077-195040867	+	Cd46 (20236)	no	0.4544	0.2872	7.4732	3.5524	563.283	0.622976
Lnc29	chr1:195131684-195135978	+	Cr1l (87)	yes	0.1449	0.0402	1.1854	0.2229	0	0.372145
5031426D15Rik	chr2:6922701-6928722	-	Celf2 (0)	yes	7.116	0.8695	9.5669	0.0824	0.0485089	0.012768
4921530L18Rik	chr2:14070332-14073934	-	Stam (166)	no	0.7371	1.1118	0.4926	0.7702	1.26397	2.29225
A930004D18Rik	chr2:18023513-18037741	-	2810030E01Rik (2184)	no	6.1805	6.9931	2.3126	4.4451	0.84388	0.481511
Gm13375	chr2:20968873-20970348	+	Arhgap21 (340)	no	2.7778	2.7345	7.782	10.0721	1.84442	1.54757
Lnc30	chr2:25475302-25478216	-	Ptgds (8067)	yes	0	0	1.1706	0	0	0
Lnc31	chr2:26608352-26619132	+	Agpat2 (4133)	yes	0.0889	0.0742	4.2118	0.0126	0.518638	0
Snhg7	chr2:26633197-26640282	-	Fam69b (0)	no	5.8005	8.3362	3.0398	18.1562	40.2218	20.6729
Lnc32	chr2:27442566-27444171	+	Brd3 (2105)	no	0.568	1.7683	1.1515	19.0001	8.15567	0.374161
6530402F18Rik	chr2:29245115-29252993	-	Ntng2 (0)	no	0.9542	0.1549	1.9016	4.8074	10.9022	0.561146
1700084E18Rik	chr2:30236387-30237720	-	Phyhd1 (23)	no	0	1.5382	0.6562	3.4345	3.56691	0.992054
Lnc33	chr2:30647569-30651971	-	Cstad (43026)	no	2.2272	1.4393	0.5626	4.7508	0	0
D330023K18Rik	chr2:31151048-31152291	-	Gpr107 (95)	no	1.6775	5.6338	5.0873	11.5917	10.2895	1.52629
Lnc34	chr2:31314519-31391743	+	Ncs1 (18416)	yes	0.4933	0.0767	7.4503	0.1403	0.249307	0.0275851

Lnc35	chr2:31391813-31396733	+	Ass1 (78456)	yes	0.0635	0	1.6707	0	0.111966	0.0669244
Lnc36	chr2:31397738-31424140	+	Ass1 (72531)	yes	0.193	0	1.5847	0.0929	0.140265	0
Lnc37	chr2:31435669-31459203	+	Ass1 (34600)	yes	0.0866	0.196	2.2529	0.176	10.1306	0.107991
Gm13446	chr2:35549533-35558702	-	Dab2ip (336)	yes	0.3893	1.0336	0.3504	0	0.158883	0.0410406
Lnc38	chr2:36216778-36223382	+	Ptgs1 (13647)	yes	0.6361	1.2639	0.6454	0.1629	0.862425	2.353
Lnc39	chr2:43195682-43196559	+	Kynu (359646)	no	0.9555	1.4097	0.1428	1.7661	0	0.867303
Lnc40	chr2:45238755-45399787	+	Zeb2 (120924)	yes	0.9568	0.2334	10.4645	0.2129	0	0.15347
Lnc41	chr2:45403622-45504113	+	Zeb2 (285791)	yes	0.2271	0.1087	3.3898	0.1147	0	0
Lnc42	chr2:48062153-48087221	-	Acvr2a (726887)	yes	0.4385	0.235	1.71	0	0	0
Gm13483	chr2:50296809-50433967	+	Mmadhc (31)	no	0.6064	0.9048	1.6792	0.3238	0.263609	0.145898
Lnc43	chr2:65024058-65025180	+	Grb14 (1284)	yes	0	0.2305	1.7526	0.0894	0	0
Lnc44	chr2:68475541-68477724	+	Stk39 (3560)	yes	0.0749	0.0832	1.4404	0.2215	0	0
Lnc45	chr2:72744564-72747650	-	Sp3 (182844)	yes	0.1309	0.3562	1.1132	0	0	0
1700011J10Rik	chr2:72979431-72989249	+	Sp3 (0)	no	3.5627	3.9485	1.7781	5.4765	4.00333	5.80405
Lnc46	chr2:73387548-73418148	+	Gpr155 (1073)	yes	0	0.0972	3.345	0.1104	0.272521	0
Lnc47	chr2:91070443-91071630	+	Slc39a13 (222)	no	0.6494	1.4717	0.7397	3.9244	6.09429	3.28819
Lnc48	chr2:91082390-91090932	+	Slc39a13 (12169)	yes	0.0494	0.3108	1.3677	0.0905	0.194544	0.851926
Lnc49	chr2:91250642-91255981	-	Arfgap2 (831)	yes	0.1212	0.1159	3.4346	0.0762	0.150853	0
2810002D19Rik	chr2:94406706-94411680	+	Ttc17 (17)	no	3.5957	4.4259	7.0997	6.8243	1.40289	3.69851
Lnc50	chr2:96318177-96568728	+	Lrrc4c (1149496)	no	0	1.0541	0	1.7047	0	0
Lnc51	chr2:103906396-103911218	+	Lmo2 (51598)	yes	0	0.1114	2.0787	0.1386	0	28.469
Lnc52	chr2:105017140-105017898	+	Eif3m (58)	no	6.3806	9.5496	5.8043	3.7424	0	8.79704
Lnc53	chr2:106642998-106650438	-	Mpped2 (42725)	no	5.435	3.8096	12.892	2.893	1.25362	0.979927
Lnc54	chr2:108948353-108950436	+	Mettl15 (143946)	no	128.103	145.071	893.6	19.4461	0.674584	0
C130080G10Rik	chr2:114054098-114088585	+	Actc1 (1287)	yes	22.948	13.9842	143.221	0.2178	0.273288	0.077127
5430417L22Rik	chr2:118745761-118748807	+	A430105I19Rik (8383)	no	21.3642	16.1251	14.4067	6.7286	4.23383	9.028
1700020I14Rik	chr2:119594071-119608320	+	1500003O03Rik (6736)	no	64.1408	44.5061	122.547	15.4902	25.1379	41.0041
Lnc55	chr2:120629131-120716473	+	Haus2 (7597)	no	1.3504	1.1898	4.1807	2.2918	1.83947	0.577472
1500011K16Rik	chr2:127791376-127792488	-	Nphp1 (3629)	no	14.129	21.8323	27.5774	32.4449	78.3143	42.6368
Gm14005	chr2:128238155-128429836	-	Anapc1 (179778)	no	5.6026	3.3334	3.7072	2.3652	0	0
Lnc56	chr2:129020255-129021877	+	Zc3h6 (1533)	no	0.2722	0.0778	1.8319	0.3269	0.216364	0.413778
Lnc57	chr2:144341125-144343439	+	Ovol2 (9045)	yes	0	0	1.3209	0	0	1.20439
Lnc58	chr2:146575431-146579801	+	Ralgapa2 (63427)	yes	0	0	2.0031	0	0	0
Lnc59	chr2:151540829-151542320	-	Fkbp1a (178)	yes	0.3578	3.2171	0.8612	0.1285	0.185927	1.33513
2500004C02Rik	chr2:153341156-153345947	-	Asxl1 (191)	no	5.3736	4.6903	2.8651	6.829	3.58776	7.99293
Lnc60	chr2:153776860-153779202	+	Mapre1 (3546)	yes	1.0002	0.089	0.0246	0.0531	0	0
BC029722	chr2:155817731-155819377	-	Mmp24 (0)	no	8.8667	4.3439	11.5981	14.6162	39.8974	5.39579
2900097C17Rik	chr2:156388062-156392979	-	4921517L17Rik (17341)	no	50.4107	57.3971	90.9498	34.8444	35.0615	28.6885
5430405H02Rik	chr2:156852402-156862945	-	Tgif2 (0)	no	0.4846	0.918	1.3611	6.5459	5.33565	5.75635
9430008C03Rik	chr2:158353610-158361530	-	Snhg11 (14107)	no	5.1932	4.5813	1.5352	10.5	10.4604	19.7504
Lnc61	chr2:166500117-166506197	-	Prex1 (60147)	yes	4.3705	0.8603	0.2876	0.1796	0.166691	0
A530013C23Rik	chr2:167691207-167697413	+	Cebpb (789)	yes	0.1445	0.039	1.2892	0	0.120026	0
Lnc62	chr2:168161619-168162144	+	Adnp (16732)	no	0.2285	2.0959	0.1631	0.2785	0.775075	1.25323
Lnc63	chr2:168766955-168777066	+	Sall4 (0)	yes	1.3315	0.251	3.9683	0.009	0	0.0704797
Lnc64	chr2:169520441-169524629	+	Tshz2 (112749)	yes	0.5963	1.1131	0.0694	0	0	0
Lnc65	chr2:173087671-173113969	+	Ctcfl (0)	yes	5.4681	1.533	0.7713	0	0.147316	0
Lnc66	chr2:175107735-175122336	-	Gm14393 (54573)	no	0.1914	0.1079	1.4631	0.5697	0.121896	0.0511718
Gm14403	chr2:177498225-177512621	+	Gm14420 (9699)	no	9.0394	2.1816	14.6352	0.4498	1.62014	0.878198
4921531C22Rik	chr2:179976852-179980716	+	Taf4a (206)	no	2.335	1.8954	2.5994	1.3968	1.16077	1.14709
Gm6307	chr2:180385603-180401802	+	Gata5 (50874)	yes	0.8296	1.4627	9.7031	0	0.0938411	0.102797
Lnc67	chr3:6138725-6138947	-	1700008P02Rik (476465)	no	48.4351	64.3172	91.7283	178.33	92.1358	0
Lnc68	chr3:19700031-19713323	+	Crh (4635)	yes	3.3825	0.7862	0.208	0.0059	0	0
Lnc69	chr3:20816225-20822192	-	Agtr1b (455015)	yes	0	0	2.57	0	0	0
Lnc70	chr3:22053249-22076209	-	Tbl1xr1 (442)	no	2.7469	3.4248	11.7431	3.8804	3.05493	1.66813
4930429B21Rik	chr3:32365488-32367438	+	Zmat3 (0)	no	1.2783	0.7751	0.132	1.0574	0.639099	0.070349
Lnc71	chr3:33977991-33993819	-	Fxr1 (26197)	yes	0	0.0486	2.3449	0	0	0
Lnc72	chr3:36354438-36408635	-	Anxa5 (40288)	yes	0	0.0569	8.5051	0	0	0

Lnc73	chr3:52173979-52179958	-	Maml3 (74952)	no	1.3634	3.189	0.1731	0.4929	0	0.334575
Lnc74	chr3:52820430-52822061	+	Cog6 (161692)	yes	0.0528	0	1.2957	0	0	0
Lnc75	chr3:53724352-53737613	+	Frem2 (66997)	yes	0.1519	0.2426	3.0012	0.1098	0	0.131442
Lnc76	chr3:57950629-57985512	+	Pfn2 (102872)	yes	0.1792	0.0152	2.2613	0.0223	0	0
Lnc77	chr3:63481126-63483902	-	Mme (99565)	yes	11.6567	1.3533	17.38	0	0	0
Lnc78	chr3:65393311-65395319	+	Ssr3 (603)	no	2.601	1.1438	3.4595	1.7832	0.0591138	0.0300729
1700113A16Rik	chr3:88171559-88177785	-	Mef2d (0)	no	0.8637	1.1574	0.2503	6.2958	15.1766	3.9872
Gm15417	chr3:89391863-89400881	+	Zbtb7b (0)	no	0.6044	0.9635	5.9948	0.6468	1.22882	1.51738
Lnc79	chr3:92428044-92429425	-	Sprr2k (3156)	no	21.719	30.2037	9.6618	33.7554	0	0.0968141
Gm9054	chr3:95985509-95986967	+	Plekho1 (3326)	yes	1.3576	0.3482	0.2968	0.2485	0	0
Lnc80	chr3:101500229-101507620	-	Igsf3 (44131)	yes	0.1862	0.143	1.6199	0	0	0
Lnc81	chr3:101712156-101713889	+	Mab21l3 (100919)	yes	1.6114	0.5377	0	0	0	0
Lnc82	chr3:105375580-105376519	+	Kcnd3 (76749)	yes	0	0.1488	1.689	0	0	0
Lnc83	chr3:105821658-105825810	-	Rap1a (24472)	yes	0.1319	0	4.4349	0	0	0.208542
AI504432	chr3:107039503-107054322	+	Kcna3 (1374)	no	0.9512	0.4151	1.0574	8.1822	0.0839768	1.98246
4933431E20Rik	chr3:107888849-107896213	-	Eps8l3 (0)	no	1.924	1.0825	1.0756	6.3006	4.47163	3.15666
Lnc84	chr3:109120667-109123047	-	Slc25a24 (100)	no	0.1995	2.6383	0.1312	2.39	0.0820787	1.09307
Lnc85	chr3:115638858-115641525	-	S1pr1 (61352)	yes	0.41	0.4902	6.0091	0	0	0.239936
A930005H10Rik	chr3:115880406-115888130	-	Dph5 (361)	no	21.7001	9.8531	12.3229	9.4379	11.6015	6.89811
A730020M07Rik	chr3:121634935-121646453	-	4930432M17Rik (24309)	yes	0.3331	0.0692	1.0937	0.0709	1.02764	0
Lnc86	chr3:121716556-121723417	-	F3 (119)	yes	0	0	2.5345	0	0	0
Lnc87	chr3:121880477-121891519	-	Arhgap29 (61784)	yes	1.8175	0.2085	0.0708	0	0	0
Snhg8	chr3:123503985-123508371	-	Prss12 (0)	no	17.6896	13.6796	7.1997	35.5361	31.043	145.265
Lnc88	chr3:127518190-127521392	-	Larp7 (15306)	yes	0	0	1.0126	0	0	0
Lnc89	chr3:134200064-134213323	+	Cxxc4 (36430)	yes	4.9951	2.9867	0.5451	0.1624	0	0.365158
Lnc90	chr3:138000975-138017271	+	Dapp1 (19194)	yes	0.3493	1.6916	0.5985	0.0278	0	0
Lnc91	chr3:138061824-138081498	+	Mttp (28030)	yes	9.3831	3.6224	19.0315	0.014	0.0123213	0.111333
Lnc92	chr3:138341648-138358810	+	Adh6a (10514)	yes	5.796	1.7138	0.0966	0.0848	0	0.122305
Lnc93	chr3:145651722-145678376	+	Cyr61 (1737)	no	3.6159	4.2533	0.9717	0.7743	0.797018	0.977073
Lnc94	chr3:151294235-151296780	+	Eltd1 (143499)	no	0.1911	0.1104	1.6172	0.2782	0.244362	0.494722
2510003D18Rik	chr3:153850373-153852424	-	Asb17 (0)	no	1.2791	0.2853	0	0.4274	0	47.7324
6330407A03Rik	chr4:3714963-3716806	+	Lyn (0)	yes	0.9818	0.1317	3.9629	0	0.292377	0.182684
1700123M08Rik	chr4:11966573-11994606	+	Pdp1 (123)	no	0.4163	0.4293	1.4408	0.9638	0.744098	0.832096
Lnc95	chr4:31455669-31493489	+	Map3k7 (508437)	yes	2.2451	1.7563	0.2107	0	0	0.0535834
Lnc96	chr4:31506435-31512057	-	Map3k7 (452049)	yes	0	2.8285	0	0	0	0
Lnc97	chr4:31824286-31877822	-	Map3k7 (86284)	yes	5.8095	6.9138	0	0.098	0	0
4933421O10Rik	chr4:33027138-33031323	-	Ube2j1 (101)	no	0.4647	0.543	1.7884	2.208	2.57043	0.867527
2010003O02Rik	chr4:40269578-40270221	+	Topors (0)	no	20.5973	25.7786	36.1259	31.8829	78.7023	47.8246
5430416O09Rik	chr4:43730033-43734534	+	Hrct1 (1923)	yes	0.3821	0.2325	3.3475	0.0338	1.59827	0.031769
A630077J23Rik	chr4:43751858-43759464	+	Olfr159 (18191)	yes	0	0	1.2111	0	0.339599	0.0864472
Lnc98	chr4:55968182-55974633	-	Klf4 (442158)	yes	0.0566	0	5.4902	0	0	0
Lnc99	chr4:66919613-66931957	+	Tlr4 (72136)	yes	0.1097	0.0136	1.8859	0	0	0.0240695
C630043F03Rik	chr4:72201243-72203930	+	Tle1 (324)	no	1.1183	3.4892	1.3571	11.4405	1.28915	2.41072
Gm5860	chr4:82065379-82102807	+	Nfib (223701)	yes	0.5438	2.225	3.273	0	3.78379	0.147798
Lnc100	chr4:82507740-82547983	+	Nfib (2432)	no	0.8239	2.1799	3.731	9.4505	0.227611	0.765396
Lnc101	chr4:88995781-89039223	-	Mtap (98146)	yes	0	0.046	2.1965	0	0	0
Lnc102	chr4:94460213-94477555	-	5830433M19Rik (22083)	no	2.1174	0.9719	5.3604	2.3349	0.716339	1.49911
E130102H24Rik	chr4:101346523-101356248	-	Ak4 (63040)	no	1.4422	0.7846	2.2512	9.0345	10.3903	0.886953
Lnc103	chr4:102188700-102195627	+	Pde4b (66041)	no	1.709	0.7228	0.0728	0.3019	0	0
Lnc104	chr4:109402053-109404970	-	Ttc39a (1790)	no	1.0522	2.3044	0.5722	1.007	1.33108	69.0592
Lnc105	chr4:109651765-109656560	-	Cdkn2c (4204)	yes	0.3046	0.0308	3.6335	0.0436	0	0.150751
C530005A16Rik	chr4:116589732-116597630	-	Gpbp1l1 (0)	no	1.2565	0.6035	2.6027	2.2406	1.1842	0.571485
Btbd19	chr4:117118482-117125725	-	Tctex1d4 (1087)	no	1.0877	1.492	1.9547	1.4889	1.11354	2.98847
Lnc106	chr4:118942349-118943446	-	Olfr1328 (8606)	no	17.3504	17.2819	0.1067	13.0659	0	0
Lnc107	chr4:120291137-120303734	+	Foxo6 (3876)	yes	0.0499	0	1.1329	0.0184	0	0.0273992
Lnc108	chr4:127280084-127308285	+	Gja4 (31336)	yes	0	0.1785	2.3941	0	0	0
Lnc109	chr4:129811180-129812302	+	Ptp4a2 (9298)	yes	7.0173	10.0884	0.6402	0	0	0.371292

E330017L17Rik	chr4:129906216-129909579	+	Ptp4a2 (56213)	yes	0	0.7575	4.0226	0.0724	0	0
Snhg12	chr4:132308663-132311024	+	Trnau1ap (3099)	no	18.64	16.4433	4.7237	31.8333	86.8608	75.1637
Snhg3	chr4:132349878-132353686	-	Rcc1 (0)	no	30.8782	30.7303	20.6489	52.8062	13.7687	299.682
Lnc110	chr4:134262943-134266187	-	Pdik1l (8729)	yes	0	0	1.1383	0.0847	0	0
Lnc111	chr4:141278460-141279726	+	Arhgef19 (20450)	yes	0.9995	2.4055	1.2206	0.1418	0.784322	0.22384
B330016D10Rik	chr4:141546161-141548313	+	Spen (7564)	no	1.7	1.2752	4.1988	3.7073	1.11135	1.23983
Lnc112	chr4:142018020-142029526	+	Fhad1 (6383)	yes	0.4031	2.2051	1.0919	0.0543	0.246177	3.6815
Lnc113	chr4:144930749-144931273	-	Dhrs3 (3628)	yes	5.0898	0.6469	0.2875	0	0	0
5930403L14Rik	chr4:154629900-154636367	-	Prdm16 (0)	no	3.4278	1.6442	3.0821	4.6135	9.21418	0.637883
2610204G22Rik	chr4:155761191-155763560	+	Atad3a (93)	no	0.2637	1.7489	1.3132	2.5559	1.91596	2.84315
Gm10560	chr4:156021644-156023824	-	Tnfrsf18 (2517)	no	0.7226	1.0194	2.4835	5.2457	1.82522	0.750419
AW011738	chr4:156203283-156211819	+	Isg15 (2465)	no	1.4603	1.4184	6.4046	1.6768	0.952469	1.29831
Lnc114	chr4:156341810-156357764	+	Vmn2r123 (2314)	yes	0.0669	0.6669	1.5643	0	0	0
Lnc115	chr5:19203015-19226646	-	Magi2 (328)	no	0.3874	1.0283	3.2814	0.8112	1.70401	0.047677
A630072M18Rik	chr5:20950937-20957807	+	Rsbn1l (0)	no	1.484	1.2914	3.5071	1.397	1.21788	2.42825
Lnc116	chr5:22553524-22740493	+	Orc5 (3166)	no	1.3596	0.7892	1.3588	0.7323	0.187519	0.383328
5031425E22Rik	chr5:23404476-23434353	-	Mll5 (68)	no	9.3726	7.2857	10.113	18.4729	8.4129	9.02594
AI506816	chr5:23692260-23712678	-	Pus7 (27341)	yes	17.9052	3.2742	5.2379	0.2355	1.91628	13.0167
2700038G22Rik	chr5:23850596-23855033	+	Tomm7 (6451)	no	3.8185	2.3718	1.5336	7.9048	1.32399	15.3418
2900005J15Rik	chr5:25100974-25103007	+	Prkag2 (281)	no	0.2644	0.0842	1.0116	0.282	2.84945	2.46818
4831440E17Rik	chr5:25499796-25504473	+	Mll3 (1013)	no	0.2891	0.2088	1.7636	1.3878	1.06727	0.968285
1700096K18Rik	chr5:25530017-25531466	+	Cct8l1 (11990)	no	3.0621	2.0074	2.4259	4.6283	7.00449	2.00404
Gm9899	chr5:30573986-30588619	-	Kcnk3 (450)	no	0.5707	0.2734	3.2914	0.2765	0.106308	0
Lnc117	chr5:31568541-31571646	-	Slc4a1ap (9518)	no	0.2985	0.258	2.0268	0.3566	0.176217	0.277087
Lnc118	chr5:36697367-36713810	-	D5Ertd579e (17145)	yes	3.1483	1.1456	1.8375	0.0426	0.193561	0.822545
Lnc119	chr5:45111699-45295287	-	Qdpr (138744)	yes	0	2.081	0	0	0	0
Gm3414	chr5:45719666-45727578	-	Lcorl (0)	no	4.123	1.987	2.1253	0.5991	1.69548	8.37692
9230114K14Rik	chr5:52190663-52205027	+	Dhx15 (144)	no	1.3215	0.8132	3.6271	0.2674	1.02292	2.02156
Gm3716	chr5:64593862-64610699	-	Klf3 (192823)	yes	4.2604	0.6945	0.242	0.0167	2.29279	0.221357
C330024D21Rik	chr5:67463897-67470831	+	Bend4 (36098)	yes	1.6674	0	0.1612	0.1072	1.00277	0.0292727
2700023E23Rik	chr5:74093082-74096002	+	Usp46 (24669)	no	2.6368	6.115	2.2764	23.5353	4.31233	24.7414
Lnc120	chr5:75734781-75740079	+	Kit (78060)	yes	0.2078	1.1471	0.2719	0	0	0.120737
Lnc121	chr5:77136059-77140275	-	Hopx (25152)	yes	0.1224	0.1752	4.719	0.0178	0.0666088	0.13012
Gm9958	chr5:90366996-90368488	+	Ankrd17 (811)	no	1.6761	1.5741	1.9765	2.01	1.28628	2.54894
Lnc122	chr5:97140819-97147861	+	Paqr3 (29223)	yes	0.4388	0	1.7846	0.0795	0	0
5430416N02Rik	chr5:100420142-100429535	-	Lin54 (12424)	no	12.4778	20.5004	3.1316	52.7356	4.07302	31.4214
D930016D06Rik	chr5:104508351-104554211	+	BC005561 (0)	no	7.1838	3.2862	5.8662	16.3598	8.02916	8.57191
Lnc123	chr5:104571627-104584889	+	BC005561 (47336)	no	3.0967	7.4855	7.1918	9.2285	3.58177	4.95508
Lnc124	chr5:107053428-107077965	+	Tgfbr3 (53141)	yes	0.8768	2.1105	0.0329	0.1332	0	0.171928
LOC100642166	chr5:109923299-109945583	+	Zfp932 (0)	yes	1.2296	0.9808	1.2356	0	6.21433	9.14794
E130006D01Rik	chr5:111734279-111761728	-	Mn1 (304703)	no	2.9535	1.6582	0.0774	5.6255	0	0.204618
1500011B03Rik	chr5:114808195-114813976	-	2610524H06Rik (7960)	no	5.966	7.6814	1.7069	79.8762	15.802	11.3017
Lnc125	chr5:119805329-119832018	-	Tbx5 (0)	yes	5.0039	5.7689	2.6739	0.0785	0	0
Lnc126	chr5:119832106-119832625	+	Tbx5 (0)	yes	5.3168	1.9447	1.3395	0	0	0
Lnc127	chr5:122093011-122099099	+	Myl2 (6623)	yes	4.7823	1.3242	12.0704	0.0132	0	0
A930024E05Rik	chr5:122989136-122998341	+	Kdm2b (0)	no	2.0375	1.8822	0.3604	5.7183	0.345353	1.09225
Lnc128	chr5:123133625-123142013	-	Rhof (9384)	no	2.0938	2.8957	6.9225	58.6775	18.0948	14.3216
Lnc129	chr5:123140856-123151992	+	Rhof (8227)	no	2.2464	1.5139	2.5496	3.0194	2.84319	2.54086
Lnc130	chr5:123152221-123160651	+	Hpd (19585)	no	2.0076	0.2276	1.5582	1.8587	1.45751	1.49142
Lnc131	chr5:123160792-123166211	+	Hpd (11014)	no	2.6023	0.3532	2.1484	4.2154	4.11452	3.2026
Lnc132	chr5:125238343-125239894	-	Scarb1 (37192)	no	1.1072	1.3984	0	1.0117	0	0
Lnc133	chr5:129844823-129846261	+	Sumf2 (1795)	no	244.924	320.288	79.3818	123.609	67.0576	164.165
4933404O12Rik	chr5:136919145-136937109	+	Rabl5 (3771)	no	1.7807	0.6488	0.2362	2.765	1.04397	2.24059
Gm20605	chr5:137629122-137643118	+	Lrch4 (0)	no	2.8633	0.8391	2.0562	9.8217	33.5121	17.4059
Lnc134	chr5:137685294-137690814	-	Agfg2 (6030)	yes	1.0357	1.7629	0	0	0	0.265934
6330418K02Rik	chr5:138264044-138267507	+	BC037034 (0)	no	2.6186	2.2525	0.857	2.0685	0.533834	1.67038
Lnc135	chr5:138996276-138998143	-	Pdgfa (3190)	no	1.3916	2.7279	1.0719	1.4162	0.413046	0

0610040B10Rik	chr5:143329286-143333320	+	Zdhhc4 (48)	no	9.5908	8.8976	11.289	3.5484	0.749966	1.99735
D130017N08Rik	chr5:143758240-143767670	+	Usp42 (21759)	no	1.3424	0.6453	2.4648	7.788	2.51634	0.887411
D5Ertd605e	chr5:147418619-147423044	+	Pan3 (11960)	yes	1.3898	0.0706	0.298	0.1926	0.363275	1.04964
Lnc136	chr5:148957902-148959431	-	Katnal1 (30449)	no	0	0.1184	2.2154	0.291	17.2444	0.896679
Lnc137	chr5:148991339-148995220	-	Hmgb1 (52006)	no	1.9461	0.8322	2.416	0.8226	2.40423	0.52072
Lnc138	chr6:6358180-6365005	+	Slc25a13 (141007)	yes	0.1271	0.1206	4.392	0	0	0
Gm16039	chr6:8259287-8429243	+	Rpa3 (80)	no	18.6998	8.6209	7.0146	14.388	17.642	13.1808
2610001J05Rik	chr6:13867443-13871505	-	Gpr85 (31617)	no	26.8745	28.1289	24.0144	21.209	26.3919	28.5163
1110019D14Rik	chr6:13871532-13898640	+	Gpr85 (31644)	no	5.1587	4.989	8.5411	0.661	3.21459	2.00501
Lnc139	chr6:17197903-17205743	+	Cav2 (83378)	yes	0.0795	0.1709	3.8778	0	0.318623	0.0968025
Lnc140	chr6:31086710-31220300	-	Mkln1 (178524)	yes	1.6304	0.118	19.5903	0.0456	2.26367	0.0451862
2210408F21Rik	chr6:31220350-31337621	+	Mkln1 (178474)	yes	1.436	0	30.8795	0	7.56212	0
1810058I24Rik	chr6:35252698-35262059	+	Nup205 (5100)	no	59.1343	54.6474	61.4364	49.3812	113.584	139.697
9330158H04Rik	chr6:36333137-36388234	-	Chrm2 (624)	yes	0.686	0.625	10.12	0.1439	0	0
Lnc141	chr6:37442329-37447336	+	Creb3l2 (138)	no	1.8755	0.6413	1.5983	0.469	0.230287	0.906059
Zfp783	chr6:47943174-47965299	+	Zfp956 (10215)	no	0.6464	1.1361	0.3616	4.1907	5.78512	6.54178
Lnc142	chr6:52102969-52114040	+	Hoxa1 (52397)	no	0	0.1878	1.7197	0.7111	3.54421	0
9430076C15Rik	chr6:53287294-53397216	+	Creb5 (0)	yes	1.9505	0.6563	0.9293	0.1473	0.345899	0.261354
Lnc143	chr6:70722480-70726781	+	Rpia (43237)	no	0.7933	0	5.0817	0.5896	52.5874	0.929344
4930414L22Rik	chr6:72438682-72440615	+	Mat2a (0)	no	1.8419	3.4552	1.2583	18.123	9.51023	4.94081
2310040G24Rik	chr6:86483375-86488266	-	C87436 (17776)	no	2.4106	4.7908	17.9983	1.2342	1.91141	1.17487
1600020E01Rik	chr6:86526751-86564655	+	Pcbp1 (586)	no	6.9987	7.9829	3.4823	37.6231	6.8779	18.0458
2610306M01Rik	chr6:86847231-86849440	-	Aak1 (52)	no	10.7978	10.5361	6.9644	8.1824	3.09263	4.56068
Gm5577	chr6:87981682-87984180	+	H1fx (200)	no	0.4575	1.1647	0.1991	18.9431	0	0.548722
1810044D09Rik	chr6:91440986-91441755	+	Chchd4 (23289)	no	3.2197	5.1415	4.1129	7.1191	16.4527	9.68243
9530026P05Rik	chr6:92940581-93243629	+	Adamts9 (0)	yes	1.6692	1.3198	8.7078	0.0596	5.81385	0.0693721
Lnc144	chr6:93153854-93162357	+	Adamts9 (210327)	yes	0	0	4.9984	0	0	0
Lnc145	chr6:93175363-93272897	+	Adamts9 (231836)	no	1.9453	2.0758	10.4169	0.3135	5.81385	0.0693721
Lnc146	chr6:99456917-99479008	-	Foxp1 (22429)	yes	0.8603	0.1866	1.38	0.2186	0.240163	0.0816661
Lnc147	chr6:108916998-108923496	-	Edem1 (61505)	yes	0.0306	0	1.3311	0	0	0
Gt(ROSA)26Sor	chr6:113067428-113077244	-	Thumpd3 (0)	no	10.8168	14.4838	11.7384	31.359	10.3214	53.777
3110021A11Rik	chr6:119848192-119850654	+	Erc1 (42)	no	0.7373	0.2156	1.2446	1.9343	0.527292	0.94539
9330179D12Rik	chr6:127149388-127212419	+	Ccnd2 (0)	yes	0.6028	0.2721	1.0258	0.1643	0	0.159188
Lnc148	chr6:127665070-127669399	+	Efcab4b (23772)	no	0.3571	0.1895	1.0593	0.3016	0	0.961824
Lnc149	chr6:128181913-128183653	-	Tspan9 (40065)	no	0.363	0.3504	1.3003	0.7168	0.254737	0.279017
Lnc150	chr6:128187497-128191044	-	Tead4 (32882)	yes	0.8946	0.3011	1.6369	0.2053	0	0.0843943
Lnc151	chr6:128313328-128313932	+	Tulp3 (7832)	no	87.0606	109.75	19.7718	124.296	0.453382	0
5930416I19Rik	chr6:128356999-128362897	-	Foxm1 (96)	no	20.746	24.2402	3.7335	11.6695	3.24656	12.8299
Gm10069	chr6:128438756-128503281	+	Fkbp4 (125)	no	1.6693	0.3063	1.0281	1.1748	0.462923	0.366928
2310001H17Rik	chr6:129232622-129238482	-	Cd69 (28842)	yes	0	0.1097	2.9153	0	6.1862	2.4228
2810454H06Rik	chr6:134897960-134900785	+	Gpr19 (0)	no	1.0935	0.6749	1.0931	1.4252	0	1.10732
1190002F15Rik	chr6:134929091-134951718	+	Cdkn1b (3566)	no	6.8283	25.3551	0.905	27.077	0.408011	132.295
Lnc152	chr6:135349236-135350022	-	Emp1 (0)	no	254.822	301.999	40.3285	518.542	0	0
Lnc153	chr6:139621858-139629745	+	Pik3c2g (94000)	yes	1.2537	0.1112	0	0	2.77674	1.06752
Gm10400	chr6:141340552-141344387	+	Pde3a (0)	yes	2.3224	0.535	5.0999	0.0263	0.0890319	0
Gm15706	chr6:145250551-145251876	+	Kras (320)	no	1.0558	1.3442	1.5707	10.3231	2.91639	2.94143
Lnc154	chr7:3422110-3423466	-	Cacng6 (163)	no	3.1345	0.9193	1.1417	1.2862	0	0
Lnc155	chr7:6343755-6355340	+	Zfp583 (12258)	no	1.0811	1.1997	0.3675	1.5981	0.0946349	0.234854
Lnc156	chr7:16872258-16874876	-	Dact3 (440)	no	0.7215	1.3359	0.3399	6.1548	0.512092	0.245073
Lnc157	chr7:19333054-19338080	+	Ercc1 (12016)	no	0.5891	1.1817	0.7306	0.4016	0.136101	0.137736
Lnc158	chr7:19463109-19474031	+	Mark4 (4375)	yes	3.7485	1.7945	0.0962	0.0556	9.35211	1.49988
Lnc159	chr7:19474419-19489759	+	Mark4 (15685)	no	2.8378	1.4742	0.3893	0.3313	19.5986	1.86006
Lnc160	chr7:19491688-19496760	+	Bloc1s3 (14115)	no	4.3987	3.3098	0.3755	0.5091	24.9141	2.56266
Lnc161	chr7:29970124-29983605	-	Zfp568 (349)	yes	0.3953	0.7033	2.7463	0.2497	0	0
Lnc162	chr7:39588847-39591529	+	Zfp619 (48432)	no	6.4477	6.6554	3.0639	5.4817	0	0
Lnc163	chr7:42105941-42107422	-	Vmn2r60 (9048)	no	38.1257	41.3984	0.2349	24.0386	0	0
Lnc164	chr7:43468432-43469102	+	Iglon5 (4474)	no	0	0	2.3499	2.3128	5.17887	0.232714

Lnc165	chr7:44480031-44486346	+	Josd2 (8373)	no	7.8877	6.0518	3.4912	0.2741	0	1.10975
Lnc166	chr7:44677298-44678232	+	Myh14 (6477)	yes	0	0	1.0494	0	1.30573	0
Gm15545	chr7:44986899-44994601	+	Prmt1 (0)	no	2.5945	1.321	2.3361	5.6212	4.64376	4.04787
Lnc167	chr7:55389985-55405726	+	Luzp2 (121097)	yes	2.913	0.9382	0	0	0	0
A230056P14Rik	chr7:55962530-55980824	+	Nipa2 (37)	no	0	1.6751	0.839	5.2197	0.765827	0.57981
Lnc168	chr7:57878169-57881301	-	Gabrb3 (52500)	no	8.5745	7.6827	0.0373	2.3645	0	0
D7Ertd715e	chr7:59968345-59975993	-	Snurf (5596)	no	12.0945	3.4344	8.7122	26.1289	0.435091	4.20746
A330076H08Rik	chr7:61929753-62093869	-	Ndn (254407)	no	1.9399	0.3907	0.3041	3.5837	0.0686182	0.667129
Lnc169	chr7:62094050-62114823	-	Ndn (233453)	no	1.8703	0.9295	0.1652	3.7083	0	0.215455
Lnc170	chr7:62206549-62209519	-	Ndn (138757)	no	5.0617	2.954	0.2609	3.4693	0	0
4833412C05Rik	chr7:67784530-67803510	-	Synm (43768)	yes	2.273	2.127	0.0805	0.0476	0	0.0771424
B130024G19Rik	chr7:70365095-70411779	+	Nr2f2 (0)	no	0.1737	0.5502	1.925	2.6075	2.1071	1.00411
1810026B05Rik	chr7:73534762-73558395	-	Chd2 (0)	no	18.4503	9.1871	16.3562	10.7007	19.451	23.5157
Lnc171	chr7:75773587-75782106	-	Akap13 (26840)	no	14.5892	3.1167	6.8129	0.8029	3.10431	6.43553
2900076A07Rik	chr7:81523549-81531498	+	Fsd2 (2630)	no	1.1828	2.093	0.833	10.4522	6.30892	6.82318
Lnc172	chr7:83755646-83757639	+	Il16 (9517)	no	159.188	226.166	50.2756	173.845	1.10726	2.44389
E230029C05Rik	chr7:90029158-90040950	+	Eed (47968)	yes	0.038	0.0749	1.4473	0.1863	0.158005	0.359987
2310010J17Rik	chr7:90124059-90129942	-	Picalm (169)	no	0.6722	1.1172	0.8058	0.9287	5.24151	6.51822
4632427E13Rik	chr7:92728597-92741459	-	Rab30 (164)	no	11.8437	8.0903	13.672	2.3214	0.623927	0.996763
Lnc173	chr7:92981904-93045075	+	Prcp (47115)	yes	0.514	0	16.3192	0	0	0
Lnc174	chr7:93148822-93150890	+	Fam181b (42509)	yes	0.1466	0	2.0801	0	0	0
Lnc175	chr7:105652204-105657225	+	Fxc1 (7349)	no	0.4548	1.1895	0.3535	2.8899	4.28714	1.75102
Lnc176	chr7:106500877-106529192	-	Olfr693 (148341)	yes	0.7312	0.7831	9.7424	0.0453	0.886872	1.4075
Lnc177	chr7:106595961-106644832	-	Olfr693 (32701)	yes	0.053	0.1848	2.9945	0.0187	0	0.727083
1700012D14Rik	chr7:111083441-111122697	-	Eif4g2 (39663)	no	1.06	0.216	1.1093	0.3826	0.302868	1.41293
Lnc178	chr7:112600229-112645528	-	Tead1 (33791)	no	1.397	0.7603	2.1329	0.2618	0	0
Lnc179	chr7:112931345-112951665	+	Rassf10 (22616)	yes	1.4572	0	0	0	0	0
Lnc180	chr7:119739969-119742218	+	Thumpd1 (19204)	no	124.872	148.948	58.6799	96.5442	0	0
Lnc181	chr7:126808171-126809250	-	Fam57b (7634)	no	3.2774	1.9414	0.1895	3.8312	0	0.918283
1700008J07Rik	chr7:127510071-127512869	-	Fbrs (17736)	no	1.5069	1.7308	1.402	3.0834	1.68478	1.95035
Lnc182	chr7:127512035-127570520	+	Fbrs (16902)	no	27.6992	8.5053	21.1015	20.3982	21.158	13.8022
Lnc183	chr7:132940441-132942274	+	Zranb1 (0)	no	1.7351	0.1754	1.2741	0.3645	0	0.279403
Lnc184	chr7:134660366-134661890	+	Dock1 (10292)	no	3.9174	3.9449	2.1139	1.5784	0	0.052724
Gm14492	chr7:142491079-142506771	-	Lsp1 (0)	yes	0.2741	0.0674	3.606	0.1533	0.936117	0.257207
H19	chr7:142575519-142578146	-	Mrpl23 (37404)	no	3390.18	4887.76	3.9266	104.951	0.454087	4192.83
R74862	chr7:143032620-143053368	-	Cd81 (10744)	no	1.3651	1.93	1.2244	5.5642	1.83149	0.585521
Kcnq1ot1	chr7:143206987-143296565	-	Kcnq1 (0)	no	7.7071	2.1323	1.9835	0.5404	0.684587	0.54517
Lnc185	chr8:3707499-3708705	-	Clec4g (7365)	yes	1.1649	0.2227	0	0	0	0.256307
Lnc186	chr8:3994491-3994986	+	Cd209c (47628)	no	12.4769	9.3951	6.8485	6.0652	0	0
Lnc187	chr8:11187749-11189408	+	Col4a1 (10673)	no	2.2607	3.316	0	0.4514	0	0
Lnc188	chr8:19516978-19519263	+	Defb7 (19203)	yes	0	1.2437	0	0	0	0
Lnc189	chr8:19681954-19687847	+	4930467E23Rik (47621)	no	0.9658	2.2483	2.433	7.0092	3.46856	6.3533
Lnc190	chr8:19701775-19704231	+	4930467E23Rik (27800)	no	1.002	0.2922	1.3159	1.5667	0.27771	0.570609
Lnc191	chr8:20008431-20013590	+	4930467E23Rik (254829)	no	1.0236	1.1347	1.246	0.6709	0.233257	0.238341
Lnc192	chr8:23383321-23388243	-	Sfrp1 (22820)	yes	0.609	1.4818	0.106	0	0	0
Lnc193	chr8:24215719-24219252	+	Zmat4 (152614)	yes	0.1697	0.1025	1.226	0	0	0
Lnc194	chr8:28593125-28597691	+	Unc5d (53591)	yes	0.3289	1.3528	1.7795	0	0	0
Gm16793	chr8:35581655-35589020	-	Mfhas1 (1223)	yes	0.9876	2.1881	7.2481	0.0859	0.703037	0.697952
Lnc195	chr8:35814752-35816421	+	Cldn23 (9956)	yes	0.0527	0.1206	1.0829	0.0436	0	0.0569134
Gm16675	chr8:46730969-46739515	-	Irf2 (229)	yes	0.4119	0.3909	1.2175	0.2193	0.507017	0.870169
Lnc196	chr8:48864195-48869809	+	Odz3 (20242)	no	1.0257	0.9531	0	2.3843	0	0.751299
Lnc197	chr8:57290925-57293191	-	Hand2 (27791)	yes	1.4957	0.0695	0	0	0	0
Lnc198	chr8:57304293-57320869	-	Hand2 (113)	no	54.9166	69.8475	66.1222	0.5303	0	4.11786
Lnc199	chr8:57331734-57332849	-	Hand2 (8332)	yes	9.1308	6.3371	5.1201	0	0	0.771409
Lnc200	chr8:57331849-57351192	+	Hand2 (7332)	yes	9.7091	13.7586	2.0192	0.0129	0.0253182	0.589133
Lnc201	chr8:57351690-57379028	+	Hand2 (27173)	yes	3.6644	1.0057	0.3597	0.0598	0	0.123662
Lnc202	chr8:57506125-57510883	+	Hmgb2 (4924)	no	5.179	7.7541	7.4804	4.3725	1.37988	8.62178

Lnc203	chr8:61921420-61927817	-	Ddx60 (81)	yes	0.5647	0.729	1.0112	0	0.323257	0.270193
Gm10664	chr8:65027465-65037336	-	Trim60 (18648)	no	24.4703	39.2175	0	1.032	86.5607	1.06445
Lnc204	chr8:67514005-67515649	-	Nat3 (8204)	no	58.8031	64.8082	64.1867	44.3885	0.0766116	0
Gm10033	chr8:69367381-69395544	-	D130040H23Rik (91453)	no	4.2356	3.5687	8.3345	1.2841	0.728644	1.33556
Lnc205	chr8:69563941-69585049	-	Zfp868 (25604)	no	1.1456	0.9965	1.6845	2.3083	0.269297	1.00137
Lnc206	chr8:69887788-69891307	-	Ndufa13 (2874)	no	1.6198	3.538	3.0938	7.1293	2.91706	0.931529
2010320M18Rik	chr8:70776782-70778291	+	Pik3r2 (70)	no	11.0608	10.5514	5.6274	25.8994	11.0949	13.6878
Lnc207	chr8:71520157-71521943	-	Plvap (10174)	yes	1.5645	0.4971	0.062	0.0663	0.112495	0.154092
Gm4890	chr8:79295077-79308721	+	Mmaa (88)	no	0.6123	1.0321	0.3427	1.1646	0.897287	0.694567
Lnc208	chr8:82132448-82142078	+	Inpp4b (4434)	yes	0.5623	0.1324	2.308	0	0	0
Lnc209	chr8:83741359-83745645	+	Cd97 (48)	yes	0	0.295	1.4704	0	0	0.25574
Gm10638	chr8:86745698-86747166	+	Siah1a (0)	no	0.8663	1.0288	0.6483	1.3214	0.324814	0.482414
Lnc210	chr8:91987406-91991767	-	Irx3 (190113)	yes	0.7058	3.0393	1.766	0.1887	0	0
4933436C20Rik	chr8:92326032-92356120	-	Irx5 (1675)	no	3.4834	5.4168	0.8616	4.5581	0.767546	2.58302
9330175E14Rik	chr8:94433126-94435103	-	Nlrc5 (702)	yes	0.06	0	1.0586	0.0305	0.201387	0.0920612
Lnc211	chr8:95599574-95600360	-	Prss54 (25163)	yes	1.6663	1.3032	0.075	0	0	0
4930513N10Rik	chr8:95806829-95821728	+	Cnot1 (0)	no	1.3742	0.3051	0.0613	0.7096	0.36968	0.467877
Lnc212	chr8:120589553-120592742	+	Gins2 (478)	yes	1.0707	0.7958	0.0371	0.0746	0.541563	0.325914
9330133O14Rik	chr8:122443614-122446476	+	Mvd (192)	no	3.4282	4.2369	2.0277	7.1423	2.7965	3.84208
2810013P06Rik	chr8:123042574-123044602	+	Ankrd11 (290)	no	4.5299	7.1408	7.9626	15.1467	5.04052	8.1822
Lnc213	chr8:126476202-126476752	-	Gm17296 (1687)	no	30.1517	44.3781	1.1289	32.9842	0	0
Lnc214	chr8:126593922-126595978	-	Irf2bp2 (2525)	no	0.7551	1.8717	0.7145	0.9266	0.409324	0.522013
Phxr4	chr9:13423360-13436894	+	Ccdc82 (130935)	no	3.4281	0.3932	6.0182	0.5588	0.729357	0.176843
Raver1-fdx1l	chr9:21067513-21092015	-	Raver1 (27)	no	2.8149	1.1285	0	4.0445	38.2988	58.1119
Lnc215	chr9:24775836-24796483	+	Tbx20 (1347)	yes	0.6907	1.2988	0.1108	0	0	0
AI414108	chr9:27352684-27357543	+	Igsf9b (903)	no	1.2197	0.8086	1.706	8.6503	1.03338	0.0449061
Lnc216	chr9:30719043-30781714	-	Adamts15 (117440)	yes	0.0367	0.0291	4.0587	0.0115	0	0
Lnc217	chr9:32348154-32348954	+	Kcnj5 (3788)	yes	0.4151	0.0809	1.6367	0	0	0
2610203C20Rik	chr9:41581338-41592487	+	Sorl1 (383252)	no	1.9541	4.7723	4.6546	170.117	1.67092	4.9876
Lnc218	chr9:44479076-44482167	+	Bcl9l (3907)	no	0.6262	1.933	0.9914	1.9263	1.65755	0.0753699
Lnc219	chr9:48161687-48249790	+	Fam55d (152668)	no	2.4995	3.7268	1.4833	2.7629	0	0.525772
2310014F07Rik	chr9:50488623-50507863	-	1600029D21Rik (2224)	yes	0.3271	0.1362	12.6279	0.0473	0.576542	0.0664063
Lnc220	chr9:55938242-55956064	+	Scaper (127)	no	1.712	1.0208	1.0176	0.5326	0.255761	0.234869
Gm20199	chr9:59476316-59485722	-	Arih1 (0)	no	12.2729	0.8054	7.1426	0.742	0.162511	0.725469
9230112J17Rik	chr9:60523660-60545887	-	Thsd4 (0)	no	0.291	0.1073	5.4943	0.3664	3.39892	0.673772
Lnc221	chr9:62414125-62415593	-	Coro2b (0)	yes	2.3859	4.0739	2.1455	0	0.360208	0.478037
9530091C08Rik	chr9:68755886-68774301	+	Rora (0)	no	1.1732	0.0645	4.8327	0.4753	0.959862	0.204619
Lnc222	chr9:71592712-71599954	+	Myzap (352)	yes	0.1032	0.4761	4.4299	0	0	0
Lnc223	chr9:72773975-72779410	-	Prtg (27863)	yes	2.799	0.195	0.0907	0	0	0
2310009A05Rik	chr9:73039717-73042775	+	Pigb (12)	no	22.4396	37.9346	28.858	21.7169	28.0317	28.796
C920006O11Rik	chr9:78175913-78178983	+	Ick (3804)	no	0.268	1.0434	1.0897	5.2942	4.19516	2.86891
Gm10635	chr9:79436891-79519302	-	Col12a1 (79688)	yes	1.2051	0.6078	2.012	0.0276	0	0
Lnc224	chr9:79759916-79762856	-	Cox7a2 (3003)	no	1.0756	0.1357	0.8303	0.3566	0.466191	0.185414
Snhg5	chr9:88519010-88522897	-	Zfp949 (25122)	no	45.3158	40.2289	7.9588	65.0587	3.45204	116.74
9430037G07Rik	chr9:88593358-88858870	-	Trim43c (10680)	no	0.8644	0.7684	2.4439	0.6631	3.00533	1.55761
9330159M07Rik	chr9:88841797-88858870	-	Trim43c (0)	no	0.4876	0.9599	2.0843	0.6399	3.00533	1.55761
Lnc225	chr9:90827631-90829359	-	Zic1 (530985)	no	0	0	1.2961	0.2663	0	0
Lnc226	chr9:90881605-91086225	-	Zic1 (274119)	yes	0.148	0.1264	9.9246	0.1218	0	0
B430319G15Rik	chr9:92537741-92542869	-	Plod2 (661)	no	1.0934	1.1163	1.2651	0.5058	0.435433	0.0343766
E030011O05Rik	chr9:96682933-96752831	-	Zbtb38 (0)	yes	2.9598	2.7522	8.8203	0.2228	8.26544	1.27545
Lnc227	chr9:107992172-107999674	+	6230427J02Rik (6256)	no	1.7896	2.9326	0.629	2.318	0.552272	3.02539
Lnc228	chr9:108912404-108913042	+	Tmem89 (2214)	no	2.9436	4.3855	11.7031	10.2539	14.7557	6.61842
Lnc229	chr9:120870161-120871966	+	Ctnnb1 (63238)	no	0.3872	1.1632	0.1506	4.462	0	0
E530011L22Rik	chr9:121756639-121759943	-	Nktr (0)	no	0.602	1.5523	1.4185	2.0076	0.914409	0.565769
1700048O20Rik	chr9:121937274-121947016	+	Fam198a (13713)	no	2.8635	1.6867	0.4056	11.4364	0.772458	6.09675
Lnc230	chr9:122903219-122914140	+	Zfp167 (4038)	no	1.4998	2.1043	0.3386	3.7585	0.0799589	0.199285
4930526I15Rik	chr9:124423255-124424856	+	Ppp2r3d (51090)	no	2.1817	4.266	0.1366	25.046	8.4566	5.89047

Lnc231	chr9:124438992-124440896	-	Ppp2r3d (33449)	no	12.5265	16.4068	8.9107	125.539	35.1951	36.6855
Lnc232	chr9:124441766-124442894	-	Ppp2r3d (31451)	no	4.0851	5.1471	0.8162	93.4962	23.0386	28.6509
Lnc233	chr10:7212275-7215095	+	Cnksr3 (38)	yes	3.1959	4.0995	16.7012	0.2304	2.13524	0.185368
A630066F11Rik	chr10:7663370-7665644	+	Pcmt1 (0)	no	1.5999	2.6804	2.2329	1.7181	9.64487	1.73731
BC020402	chr10:7677747-7681158	-	Nup43 (0)	no	1.0447	2.0481	2.4502	4.3678	2.29509	2.53678
C920009B18Rik	chr10:22306719-22314265	+	Raet1a Raet1c Raet1b (0)	yes	0.0683	0.1334	1.5501	0.0096	0	0.295087
Lnc234	chr10:22814867-22816043	+	Tcf21 (2407)	yes	0	1.9817	0.3017	0	0	0
D830005E20Rik	chr10:33193792-33256422	-	Trdn (0)	yes	1.5064	0.1973	70.0707	0	0	0
A830082N09Rik	chr10:33987215-33995055	-	Rwdd1 (1499)	no	1.2482	0.8142	1.5428	2.4192	0.347387	0.185139
E130307A14Rik	chr10:39621410-39732007	-	Traf3ip2 (0)	no	5.6057	5.4083	2.0577	22.4739	1.42368	1.84556
Lnc235	chr10:43523779-43524746	+	1700021F05Rik (1341)	no	11.0934	17.3898	18.0209	14.9677	0.656918	0.0908999
Lnc236	chr10:53181017-53207327	+	Slc35f1 (69395)	yes	0.2095	0.191	3.2798	0.0306	0	0
Lnc237	chr10:56967026-56982229	-	Hsf2 (504155)	yes	0.713	0.1444	25.1467	0.0133	0	0
Lnc238	chr10:57116877-57158975	+	Hsf2 (369507)	yes	0.5506	0.0523	3.3656	0.0386	0	0
2310015B20Rik	chr10:70204666-70219711	+	Ccdc6 (11180)	no	0	0.2226	2.1592	0.6761	0	2.87724
Gm17769	chr10:77418375-77420939	+	Adarb1 (102)	no	1.0167	0.7706	1.1699	0.407	0.0766855	0.107763
Lnc239	chr10:78248746-78263917	-	Agpat3 (3129)	no	0.2157	0.2028	1.0464	0.9339	1.62267	0.255519
E130317F20Rik	chr10:79851376-79854971	-	Ptbp1 (367)	no	3.0563	2.4695	1.2382	1.9867	0.773341	0.74907
2310050B05Rik	chr10:81194676-81211285	+	Dapk3 (149)	no	20.4063	22.9197	9.2086	0.8108	0.0946982	0.189489
Lnc240	chr10:82354268-82404689	+	Zfp938 (112904)	no	1.5673	1.4149	0.8841	0.9976	0.418252	0.223501
Lnc241	chr10:82614175-82618300	-	1190007I07Rik (945)	no	0.3999	0.4242	1.8136	3.1178	0.39699	1.00094
Lnc242	chr10:89686406-89689256	+	Scyl2 (116)	no	1.8211	5.2207	5.5162	31.8351	10.3446	4.35737
LOC627800	chr10:92171300-92471244	-	Nedd1 (213375)	yes	0	0	1.2988	0.033	0	0
Lnc243	chr10:93317330-93322684	+	Elk3 (6171)	yes	0.2886	0.0629	2.6369	0.1557	0.091811	0.329207
4932415G12Rik	chr10:94673492-94688613	-	Ccdc41 (103)	no	3.1653	2.919	2.696	2.8124	4.33209	2.37279
2310039L15Rik	chr10:95336209-95364449	+	Cradd (12112)	yes	0.1958	0	6.8146	0	2.69371	0.296899
Lnc244	chr10:99279889-99285107	-	Dusp6 (16698)	yes	2.6477	1.5344	0.0496	0	0	0
Lnc245	chr10:108444195-108444766	-	Pawr (30375)	yes	0.7285	1.5136	0.2619	0	0	0
Lnc246	chr10:110124389-110217192	-	Nav3 (216608)	no	5.8282	3.3599	1.1307	7.4627	0	0.0657353
Lnc247	chr10:116473999-116474963	+	Kcnmb4 (476)	no	0.2806	1.1952	0	1.258	0	0
4933412E12Rik	chr10:116950561-116963279	+	Rab3ip (181)	no	0.9515	1.0299	1.4015	0.2707	2.35606	0.303417
Lnc248	chr10:120201835-120203482	+	Irak3 (298)	yes	0.0856	0.2841	1.0844	0.2161	0.806584	0
Lnc249	chr10:121510299-121523150	+	Rassf3 (34049)	yes	0.455	1.2458	0	0.0724	0	0.845077
1700012D01Rik	chr10:127667122-127669050	+	Nab2 (0)	yes	1.4206	1.0257	4.2882	0.1975	1.05235	0.535605
Gm11944	chr11:3308999-3322220	+	Patz1 (0)	no	3.0037	0.6233	0.8631	1.2376	21.2366	20.1451
8430429K09Rik	chr11:3452383-3479831	+	Rnf185 (58)	no	2.0251	2.3947	1.8491	4.3993	0	0
Tug1	chr11:3639591-3649025	-	Morc2a (218)	no	90.9288	59.0514	40.4861	26.966	22.3884	17.7451
2210015D19Rik	chr11:5761980-5788435	+	Urgcp (0)	no	5.7684	4.2198	6.8526	6.215	18.7026	5.37259
Gm11974	chr11:6525590-6528788	-	Myo1g (7830)	no	5.9197	6.2589	0.7648	7.819	5.38628	34.4418
Lnc250	chr11:12040165-12048137	+	Grb10 (2745)	no	2.6233	0.8791	0.2736	0.4295	0.349444	0.420107
Lnc251	chr11:12053921-12191495	+	Grb10 (16501)	no	2.1044	1.7492	0.3723	0.7919	0.384975	0.121069
Lnc252	chr11:15049179-15211395	+	Pom121l12 (448863)	no	99.6816	161.172	54.025	432.384	0	0
Lnc253	chr11:16514453-16516032	+	Sec61g (5969)	no	29.6306	36.2911	45.5463	26.4409	0	0
Lnc254	chr11:17954022-17955242	+	Etaa1 (147)	no	1.4768	1.766	0.4625	0.7704	6.25057	1.93857
Lnc255	chr11:29612412-29645590	-	Rtn4 (47298)	yes	0.256	0.0698	2.2609	0	0	0
Lnc256	chr11:32902811-32926147	-	1700008A04Rik (101553)	yes	0.137	1.0673	0	0.0825	0	0
Lnc257	chr11:41498546-41500770	+	Gabrg2 (411648)	no	155.877	190.157	99.3159	22.2659	0	0
Lnc258	chr11:44143393-44196289	-	Il12b (203773)	yes	0	0	1.4939	0	0	0
Lnc259	chr11:57795494-57801461	-	Sap30l (175)	no	0.9783	2.6649	1.245	2.3665	6.74918	2.45853
Lnc260	chr11:57832766-57841612	+	Hand1 (619)	yes	2.3825	0.4108	0	0	0	0.111994
2610507I01Rik	chr11:59196891-59202431	-	Mrpl55 (74)	no	1.0192	5.5419	13.5527	13.9211	6.06283	11.7096
4933439C10Rik	chr11:59505684-59511329	+	Zkscan17 (0)	no	2.4885	3.3621	1.4942	58.0634	11.4225	6.03539
1700013G23Rik	chr11:59946891-59948147	-	Med9 (66)	no	0.9551	1.4162	1.6892	1.5954	1.18975	0.502654
Gm16516	chr11:60919235-60931867	-	Map2k3 (189)	no	1.3302	1.4921	8.5807	1.5993	1.74248	3.31807
2410006H16Rik	chr11:62602876-62609908	+	BC046404 (1929)	no	74.1026	82.6231	4.8207	162.099	27.7422	236.961
2810001G20Rik	chr11:64079483-64083970	+	Cox10 (11)	no	6.2254	13.8374	11.9931	19.2733	5.31258	6.33999
Gm12295	chr11:65275121-65366053	-	Myocd (95582)	yes	0.3779	0.2946	2.1419	0.0147	0	0

Lnc261	chr11:65302759-65304043	-	Myocd (33572)	yes	0.3498	0.2214	1.8735	0	0	0
Lnc262	chr11:65418463-65422024	-	Myocd (151553)	yes	0.5101	1.0637	0.1946	0	0	0
Lnc263	chr11:65634428-65648685	-	Map2k4 (39525)	yes	1.3699	0.5741	1.9424	0.0046	0	0.225022
A030009H04Rik	chr11:69340768-69342647	+	Chd3 (2217)	no	4.9318	5.6967	1.0424	50.8143	0.988307	0.950263
2210403K04Rik	chr11:75461538-75467455	+	Tlcd2 (6372)	no	2.4435	2.2418	10.1889	2.172	213.224	17.6229
Lnc264	chr11:78293856-78295169	+	2610507B11Rik (114)	no	0.1934	0.3253	1.7972	0.2992	0.218682	0.412488
Lnc265	chr11:82779245-82781059	-	Lig3 (75)	no	1.3871	1.2978	1.5457	1.4949	1.62948	1.84857
Lnc266	chr11:82909090-82910847	-	Unc45b (405)	yes	1.5355	0.4039	1.0366	0.0311	0	0.0913835
AI662270	chr11:83223575-83226584	+	Slfn4 (0)	yes	0.2048	0.754	10.4189	0.2405	0.802208	179.716
Lnc267	chr11:83243825-83254439	+	Slfn4 (0)	yes	0	0	1.783	0	0	9.04198
AA465934	chr11:83291698-83294929	+	Pex12 (2946)	no	15.319	2.6041	0.9179	4.4218	19.8141	12.8808
Lnc268	chr11:84870891-84872956	+	Ggnbp2 (114)	no	0.8148	1.2434	1.0144	2.3432	3.14591	5.94212
Lnc269	chr11:88253401-88254299	+	1700106J16Rik (40641)	no	0.1734	0.6626	1.4989	0.45	0	0
C030037D09Rik	chr11:88718642-88732468	+	Msi2 (375)	no	2.6194	6.6981	4.2616	2.3216	3.76831	0.858841
Lnc270	chr11:89066466-89067841	+	Dgke (5718)	no	0.3237	1.4035	0.2442	0.8943	0.315801	0
Lnc271	chr11:94133462-94155748	+	Spag9 (7339)	no	0.8186	3.323	0.5863	0.557	0.391713	0
B130006D01Rik	chr11:95723585-95726773	+	Zfp652 (0)	yes	0.7926	0.311	1.6446	0.1771	0	0.167586
4833417C18Rik	chr11:95858816-95861046	+	B4galnt2 (4742)	yes	0.6694	0.3762	1.059	0.1888	0.763538	4.33111
Lnc272	chr11:96007309-96008151	+	Igf2bp1 (1218)	no	1.6339	1.1364	0	1.4205	0	0
D030028A08Rik	chr11:96944145-96967019	+	Pnpo (126)	no	0.4347	1.2009	0.6812	4.1022	1.28427	19.5796
Lnc273	chr11:97662419-97663128	+	Mllt6 (992)	yes	0.2139	0.1847	1.1273	0.0982	0	0
Gm11627	chr11:102576397-102579323	-	Gpatch8 (22927)	no	5.9864	8.9433	1.4475	1.9169	1.72457	0.174874
2810433D01Rik	chr11:102619506-102624416	-	Fzd2 (16358)	yes	0.4224	1.7586	0.2421	0.183	0.8142	0.165929
Lnc274	chr11:105291128-105292445	-	Mrc2 (200)	no	0.5774	3.1759	0.3993	0.7034	1.80977	0.0732062
Lnc275	chr11:106364990-106368232	+	2310007L24Rik (9835)	yes	0	0.0489	3.7333	0.0637	0.188013	0
Lnc276	chr11:107201914-107206530	+	Pitpnc1 (5972)	yes	1.567	1.0143	7.9548	0.155	0.155426	0
LOC100503496	chr11:109440077-109453359	+	Amz2 (1929)	yes	1.2544	0.8981	2.5483	0.1264	0.578585	0.0459783
2610035D17Rik	chr11:113043829-113201838	-	Slc39a11 (43016)	no	1.0379	4.4634	4.993	14.8592	5.80841	1.51004
1810032O08Rik	chr11:116671659-116678064	+	Gm11744 (3270)	no	8.2261	5.4987	2.346	24.9123	13.9335	34.7731
2810008D09Rik	chr11:117076782-117078955	+	Sec14l1 (38389)	no	5.8669	9.0526	4.794	110.497	26.512	38.064
Lnc277	chr11:117094528-117097151	-	Sec14l1 (18020)	yes	0.0484	0.0512	1.8664	0.0627	0.230965	0
Lnc278	chr11:117523647-117533973	+	Tnrc6c (130641)	no	0.0436	0.0712	1.5907	0.9426	0.970822	0.0534776
Lnc279	chr11:119392877-119450160	+	Slc26a11 (11801)	no	13.1706	3.6549	23.3372	0.5026	6.68454	3.89043
2810410L24Rik	chr11:120186554-120189856	-	Slc38a10 (38505)	no	2.0295	1.3458	0.5861	5.084	1.94025	1.74933
0610009L18Rik	chr11:120348677-120353464	+	Actg1 (193)	no	1.7792	3.697	5.0365	5.541	13.772	1.73018
Lnc280	chr11:120762790-120763177	+	Rfng (17954)	no	156.619	64.3277	13.4865	7.8111	0	0
1700012B15Rik	chr12:3235456-3250374	+	Rab10 (11971)	no	2.2001	1.4081	1.6085	6.824	18.8944	16.9838
1110002L01Rik	chr12:3403882-3426747	-	Kif3c (0)	no	8.3373	6.4386	3.7908	4.666	4.02729	6.07295
Lnc281	chr12:16006403-16015505	-	Trib2 (198646)	yes	0	0.1085	1.6545	0	0	0
Lnc282	chr12:16139887-16221481	-	Lpin1 (314187)	yes	0.0714	0.039	3.9828	0	0.126905	0
Lnc283	chr12:17882107-18063714	+	Hpcal1 (90050)	no	2.4091	1.8901	0.0217	5.5067	0.296021	0.22875
Lnc284	chr12:20224715-20235182	+	Asap2 (765752)	yes	2.0518	0.2369	0.1912	0.0952	0	0.0532297
1700030C10Rik	chr12:20804391-20815779	-	Asap2 (174688)	no	0	1.0178	0.1232	0.9857	3.13622	1.67703
Lnc285	chr12:20913615-20921246	-	Asap2 (69221)	no	0.081	1.4998	0.9875	3.0065	2.08344	0.326141
Lnc286	chr12:21417914-21419037	+	Ywhaq (478)	no	1.8877	0.4823	0.4684	1.7853	0.341061	0.628365
2410018L13Rik	chr12:22953970-23010243	-	9030624G23Rik (1032958)	no	0	1.1939	0	5.5961	1.58679	1.17557
Lnc287	chr12:24635244-24651023	-	Klf11 (347)	no	44.2231	9.1927	8.0514	0.9231	0.175398	0.132758
Lnc288	chr12:34051138-34107280	-	Twist1 (147449)	no	1.2823	1.0849	0.5461	0.6209	0.109393	0.278573
Lnc289	chr12:34710016-34715984	-	Hdac9 (186820)	yes	0.2697	0.1044	2.142	0.098	0.0471255	0.104202
Lnc290	chr12:34826853-34906686	-	Prps1l1 (78074)	no	0.3937	0.9714	1.748	0.466	0	0
Lnc291	chr12:37095738-37106907	-	Meox2 (1638)	yes	0.4688	0.0231	6.6036	0	0	0
Lnc292	chr12:39397570-39463979	-	Arl4a (568233)	yes	2.9818	2.8733	0	0	0	0
Lnc293	chr12:41024093-41124014	+	Dock4 (176049)	yes	1.5205	0.4467	3.0504	0.108	0.381434	0.421153
Lnc294	chr12:41191469-41204534	+	Lrrn3 (260198)	yes	1.6632	0.1458	2.7184	0.0447	0.154353	0.220269
Lnc295	chr12:48554203-48556447	-	Foxg1 (826435)	no	4.1948	3.5942	8.7292	2.8508	0	0
Lnc296	chr12:51829868-51835023	+	Hectd1 (332)	no	0.9873	0.6981	2.384	0.2532	0.593911	1.10656
Lnc297	chr12:69122873-69123808	+	Rps29 (34848)	no	16.7068	19.0947	10.6638	5.739	0	0

9330151L19Rik	chr12:69197210-69199868	+	1110034A24Rik (0)	no	1.994	1.9939	3.6264	19.7591	5.14331	9.68954
Lnc298	chr12:70746786-70816496	+	Frmd6 (78677)	yes	0.615	0.0743	6.1996	0	0	0
3110056K07Rik	chr12:70991614-71015832	-	Psma3 (0)	no	2.24	2.8262	2.9856	15.8649	3.8065	10.5879
Lnc299	chr12:71337698-71343661	+	Dact1 (17591)	yes	1.3673	0.5287	0.9801	0.1024	0	0
Lnc300	chr12:72023274-72027580	+	Daam1 (30907)	yes	1.6118	0	0.0683	0	0	0
D830013O20Rik	chr12:73364074-73409570	-	Slc38a6 (55525)	yes	0	0	2.8004	0	0	0.0317356
Lnc301	chr12:73794526-73808352	+	Prkch (16342)	yes	0	0.3437	4.6787	0	0	0
Gm10451	chr12:76443956-76450953	+	Ppp1r36 (4465)	no	1.4673	1.7784	0.8038	0.4478	9.46979	3.20408
Lnc302	chr12:80077099-80079836	-	Zfp36l1 (27923)	yes	0	0	1.3195	0	0	0.137977
Lnc303	chr12:80084195-80090683	-	Zfp36l1 (17076)	yes	0.409	0	1.1003	0	0	0
2310015A10Rik	chr12:80120473-80132844	-	Zfp36l1 (19831)	no	1.9959	2.1395	5.2584	2.6236	1.54659	1.49312
2310002D06Rik	chr12:80507205-80517954	+	Exd2 (8367)	no	0	0.0924	1.4772	0.5107	0	0
2310044G17Rik	chr12:86947042-86965486	+	2310044G17Rik (0)	no	14.8752	9.1316	21.2564	28.8017	12.3358	9.32009
Lnc304	chr12:98902494-98910357	-	Eml5 (8873)	yes	0.2929	2.5786	0.0568	0.0247	0	0
Lnc305	chr12:101968095-101976268	-	Cpsf2 (667)	no	82.938	121.568	214.15	54.5838	78.2402	193.381
Snhg10	chr12:105030616-105032279	-	Glrx5 (409)	no	2.4212	5.3765	0.7885	11.5678	0.788899	8.41703
Meg3	chr12:109540995-109572475	+	Rtl1 (49173)	no	13.7424	10.8246	2.5894	745.336	6.3717	521.5
Lnc306	chr12:109576211-109577871	+	Rtl1 (13957)	no	2.1085	0.6238	0.7035	0.7582	0	1.14685
Rian	chr12:109591536-109693405	+	Rtl1 (0)	no	28.7227	44.0557	3.4759	238.017	1.84731	451.242
Lnc307	chr12:109683309-109693405	+	Rtl1 (87906)	no	38.0905	60.7471	5.8951	260.009	1.84731	451.242
Mirg	chr12:109729663-109749457	+	Rtl1 (134260)	no	3.6121	8.4627	0.5962	36.3365	0.431049	47.2129
Lnc308	chr12:110599726-110601300	-	Dync1h1 (94)	no	0.2685	1.8205	1.0989	0.7139	0.474423	0.237549
2810029C07Rik	chr12:111572316-111574402	-	Mark3 (107)	no	0.5297	1.1007	0.5795	7.4326	2.04422	0.533537
5033406O09Rik	chr12:111941990-111944482	-	2010107E04Rik (16893)	yes	0.9105	1.3508	0.3934	0.2487	0.49231	4.29142
Lnc309	chr12:112783106-112795733	-	BC022687 (13241)	yes	0.3335	0.1437	1.0082	0.0401	0.0691796	0.0681849
Lnc310	chr12:113287305-113296062	-	Tmem121 (106540)	yes	0	0.017	1.4938	0	0.0883339	0
Lnc311	chr12:113414315-113416272	-	Adam6b (73292)	yes	0.1495	0	1.5703	0	0.34548	0.137875
Lnc312	chr12:113418620-113430697	-	Adam6b (58867)	no	0.4418	1.0404	5.5801	1.6569	8.09157	5.95749
D430020J02Rik	chr12:116398889-116405165	-	Ncapg2 (236)	no	3.7865	3.5284	0.6124	4.8183	0.202451	6.71885
2810429I04Rik	chr13:3478245-3501489	+	Gdi2 (59829)	no	1.3371	4.1423	0	1.08	0	0.297727
1700024F13Rik	chr13:3498049-3501393	+	Gdi2 (40025)	no	0.3373	3.7361	0	1.4923	0	0.297727
Lnc313	chr13:5601389-5603770	-	Klf6 (257693)	yes	0	0	2.0471	0	0	0
Lnc314	chr13:9670312-9672725	-	Dip2c (3099)	yes	1.1789	0	0.0667	0.104	0	0
Lnc315	chr13:11522657-11539110	-	Ryr2 (10714)	yes	2.2964	0.0854	1.4845	0	0	0
Lnc316	chr13:12156234-12172161	+	Mtr (26396)	yes	0	0	1.9286	0	0	0
Gm10336	chr13:12182716-12186488	-	Mtr (0)	no	7.6503	4.2855	58.3956	5.6201	8.61326	12.5796
Lnc317	chr13:17944472-17951145	+	Rala (255)	no	1.0027	1.5021	3.4753	2.4212	0.176265	0.201765
C230035I16Rik	chr13:23426608-23431281	-	Abt1 (7415)	no	0.6408	2.3132	3.0438	2.8667	0.978183	0.742515
Lnc318	chr13:28408255-28410644	-	Prl5a1 (259049)	no	0.0493	0.0268	1.4109	1.7655	0	0.141563
2610307P16Rik	chr13:28460033-28885422	-	Sox4 (62203)	no	0.978	1.6327	1.6618	5.6522	1.27367	0.666698
A330102I10Rik	chr13:29014413-29040336	+	Sox4 (60582)	no	0.4596	0.707	2.1686	9.8247	0	0
Lnc319	chr13:45965247-45966505	+	Atxn1 (242)	no	6.4707	8.5795	3.0873	3.6436	4.01774	1.6039
C030044B11Rik	chr13:48968111-48970313	+	Fam120a (283)	no	8.3211	11.5402	14.3178	18.8193	18.1711	6.76418
Lnc320	chr13:53016201-53018488	+	Nfil3 (34335)	yes	0.128	0.0754	2.0806	0	0.348099	0.171537
Lnc321	chr13:53052949-53069494	-	Ror2 (39762)	yes	0.4865	0.0282	1.7638	0.0208	0	0
Lnc322	chr13:53911065-53915760	-	Drd1a (135422)	yes	0	0.9921	4.958	0	0	0
Etohd2	chr13:59769965-59773680	+	Isca1 (160)	no	0.4336	0.7328	2.1963	2.0141	1.42646	2.47994
Lnc323	chr13:62486441-62492420	-	6720489N17Rik Zfp935 (0)	no	1.1407	1.6379	0.471	1.0802	0	0.227242
1810034E14Rik	chr13:64248699-64268703	+	Cdc14b (0)	yes	1.1518	2.0922	4.007	0.1647	5.57258	2.63474
Lnc324	chr13:69535028-69537188	+	Papd7 (1164)	no	0.4117	0.5522	1.526	0.4564	0.856234	0.597786
Lnc325	chr13:72324011-72371945	-	Irx2 (257032)	yes	2.7465	1.137	0	0	0	0
Gm20554	chr13:72620376-72628564	-	Irx2 (413)	no	1.3327	0.422	0.2116	1.9381	1.18472	0
D630045M09Rik	chr13:73344384-73347384	-	Mrpl36 (14639)	yes	5.3132	5.9312	1.0537	0	8.7892	0
Lnc326	chr13:74008151-74009265	-	Tppp (153)	no	1.9725	1.632	1.9978	1.9422	1.67153	0.43042
A830082K12Rik	chr13:78198016-78276042	+	Nr2f1 (0)	no	0.5723	1.223	1.2014	4.3262	0.225452	0.149749
Lnc327	chr13:89500409-89520012	-	Hapln1 (20623)	yes	2.0966	0.0881	0	0	0	0.0587872
4833422C13Rik	chr13:91701664-91741872	-	Ssbp2 (0)	no	0.0652	0.0453	2.5718	2.0551	2.07332	1.69566

Lnc328	chr13:92022568-92131656	-	Msh3 (78567)	no	0.545	0.183	4.2051	0.8412	0.597852	0
Gm9776	chr13:94356748-94358923	-	Ap3b1 (21)	no	1.1053	0.736	0.3521	2.113	1.12285	1.08688
2310020H05Rik	chr13:99076494-99088026	-	Zfp366 (96568)	yes	0.1343	0	1.0759	0	0.270835	0
Lnc329	chr13:99516645-99526222	+	Mtap1b (43)	no	3.1124	3.4178	0.1583	0.6456	0.194548	0
Lnc330	chr13:102349708-102359838	+	Cd180 (343849)	yes	0.1479	0.3028	15.5155	0	0	0
Lnc331	chr13:112100877-112101774	+	Ankrd55 (187573)	no	0.4453	2.9038	0.7008	0.9173	3.3027	0.21741
Lnc332	chr13:112764792-112765680	+	Slc38a9 (26040)	yes	0	0	4.9754	0	0	0
Lnc333	chr13:113293303-113370615	+	Esm1 (75199)	no	2.5142	2.3768	0.7893	0.6867	7.08301	0.576597
Gm6416	chr13:117127031-117136272	+	Emb (93541)	yes	0.1261	0.0824	6.9141	0	0	0
3110070M22Rik	chr13:119487256-119488384	-	Gm7120 (346)	no	0.9616	0.9606	1.0805	1.1316	0.640669	0.178299
Lnc334	chr13:120041361-120045896	-	LOC639910 (94350)	yes	0	0	2.33	0	0.132664	0.0842734
Lnc335	chr14:4334771-4339919	+	Gm3264 (96220)	yes	6.9068	0.1441	0.2006	0	0	0
1810062O18Rik	chr14:20546292-20570680	+	Ppp3cb (0)	no	0.4565	1.5036	0.2744	0.446	0.249828	0
6230400D17Rik	chr14:20695362-20703099	-	Fut11 (0)	no	0.554	0.4182	1.1228	4.7242	1.01695	1.74407
Lnc336	chr14:20794228-20796542	+	Camk2g (140)	yes	1.95	2.6753	0.4137	0.1581	0.425673	0.956836
4931406H21Rik	chr14:25586803-25590661	+	Zmiz1 (0)	no	1.7102	0.2823	3.3078	0.2989	0.288605	0.106336
Lnc337	chr14:25670071-25687767	-	Ppif (6401)	yes	0.0867	0	1.9617	0.0835	0	0
2010107H07Rik	chr14:31123808-31129110	-	Nt5dc2 (2023)	no	13.3099	17.1832	14.2988	31.0947	101.551	46.3946
Lnc338	chr14:32685379-32688170	+	3425401B19Rik (0)	yes	1.1777	0.9023	1.5306	0	0	0.0527965
Lnc339	chr14:34119557-34120849	+	A630023A22Rik (16803)	yes	0	0	6.5153	0	0	0
Lnc340	chr14:34661968-34662766	+	Wapal (11938)	yes	0	0.282	1.9295	0.1056	1.04452	2.23486
Lnc341	chr14:43120583-43122246	-	Gm10377 (106670)	no	29.3652	36.4194	15.0254	3.8218	0	0
Gm1821_2	chr14:46084026-46084957	+	Bmp4 (299493)	no	384.1	263.378	116.385	961.354	76.7317	21.9748
Lnc342	chr14:46645603-46681439	-	Cdkn3 (78974)	yes	0	1.0173	0	0	0	0.238412
Lnc343	chr14:49645487-49646526	-	3632451O06Rik (27865)	yes	1.2374	0	0.0813	0	0	0
Lnc344	chr14:49807216-49823228	+	3632451O06Rik (13158)	yes	132.462	0	0	0	0	0
Lnc345	chr14:54181069-54223732	+	Dad1 (54415)	no	1.0179	1.0913	1.187	0.3631	4.66194	0.555749
Lnc346	chr14:55420344-55420782	-	Dhrs4 (57922)	no	182.706	287.007	77.4491	12.1016	71.1855	0
Gm16973	chr14:56696274-56701000	-	Pspc1 Mphosph8 (0)	no	3.4053	2.1363	2.3829	10.4908	5.29925	42.6872
Lnc347	chr14:58300799-58303933	+	Fgf9 (188065)	yes	0.4952	1.0745	0.21	0.045	0	0
Dleu2	chr14:61602835-61682373	-	Trim13 (0)	no	1.0947	2.994	7.5004	1.6136	4.37286	8.02152
2700070H01Rik	chr14:63055072-63058149	+	Defb42 (6466)	yes	1.3372	2.1917	0.031	0.0789	0.0961466	0
Lnc348	chr14:63317026-63345816	+	Gata4 (45108)	yes	0.907	1.4833	3.8327	0.0103	0	0.0580916
Lnc349	chr14:63360034-63368910	-	Blk (3926)	yes	2.2506	3.1849	0.7572	0	0	0.0746412
Lnc350	chr14:69732817-69767267	+	Chmp7 (247)	no	0.7504	1.2508	2.2256	0.5906	0.289616	0.454813
Gm4285	chr14:75842851-75845089	-	Tpt1 (166)	no	0.8905	1.5818	1.0486	1.1562	1.56189	1.62325
Lnc351	chr14:100242445-100249500	-	Klf12 (0)	no	0.6713	0.2518	1.952	0.6696	0.181863	0.722349
Gm17066	chr14:105106727-105114676	-	Rbm26 (0)	no	29.3066	7.6408	10.7051	34.6464	6.5833	26.5323
Lnc352	chr14:105589349-105594788	+	Ndfip2 (279437)	no	1.5424	1.5771	1.2232	0.356	0	0
Lnc353	chr14:111588193-111676459	-	Slitrk5 (1571)	no	2.4425	1.9862	0.1162	1.2497	0	0.0127524
Mir17hg	chr14:115043755-115050860	+	Gpc5 (48459)	no	6.2907	7.7707	4.3481	16.6511	1.50152	45.867
BC037032	chr15:4020110-4027406	-	Oxct1 (1666)	no	0.2801	0.484	1.7747	0.3892	0.203846	0.0292334
Lnc354	chr15:5120586-5121282	-	Rpl37 (1055)	no	179.003	320.905	599.368	110.143	7.02822	0.439929
0610007N19Rik	chr15:32240567-32244662	-	Sema5a (150)	no	5.2717	8.1923	4.2701	6.0204	23.209	5.153
Lnc355	chr15:32778876-32795932	+	Sema5a (82379)	no	0.5502	1.3069	1.6478	1.2333	0.332865	0.219447
Lnc356	chr15:38450942-38455243	-	Azin1 (27990)	yes	1.7293	1.1874	0	0	0.324634	0
Lnc357	chr15:44643399-44644760	+	Ebag9 (2373)	yes	0.3459	1.0699	0.2734	0	0	0.114882
2310069G16Rik	chr15:44787762-44805829	+	Sybu (0)	no	0.3662	0.5996	2.7323	3.5917	0	0
9930014A18Rik	chr15:60822964-60831400	+	Fam84b (0)	no	0.8421	1.0205	0.8986	0.6685	9.7434	1.05879
Pvt1	chr15:62037980-62250975	+	Myc (47619)	no	1.6342	1.4786	1.5529	0.4829	0.860462	4.65173
Lnc358	chr15:62980637-62981835	+	Gsdmc (795333)	no	19.6701	33.5824	15.6882	34.3087	0	0
Peg13	chr15:72805599-72810324	-	Trappc9 (0)	no	4.4164	2.9118	16.6759	77.4153	20.1972	2.0297
Lnc359	chr15:76360361-76361388	+	Maf1 (5983)	no	0.3872	0.2138	1.2233	2.2653	1.20037	0.975856
Lnc360	chr15:77003708-77013686	-	Mb (1794)	yes	2.6877	0.3823	0.5459	0.0291	0	0
Gm16576	chr15:79742697-79757394	+	Sun2 (145)	no	0.3113	0.2682	1.1175	1.2093	1.03427	0.20458
D730005E14Rik	chr15:79889531-79893138	-	Apobec3 (792)	yes	0	0.09	1.3741	0.0508	0.151366	1.04446
Tbrg3	chr15:82889294-82898934	-	Tcf20 (0)	no	3.1815	0.4024	2.7493	0.5075	0.354137	0.37523

1700001L05Rik	chr15:83353607-83367297	-	Pacsin2 (3364)	yes	1.2318	0.4864	0.4058	0.1646	1.47189	2.46998
Gm19277	chr15:85149253-85156182	-	Ribc2 (11613)	yes	0.6954	0.0715	2.3915	0	0	0
Lnc361	chr15:85646108-85684635	+	Wnt7b (64287)	no	0.226	0.5941	4.1082	0.7028	1.60388	0.110729
Lnc362	chr15:85691851-85701079	+	Ppara (43712)	no	0.1128	0.3521	2.4886	0.5184	0.806844	0.0285868
1810021B22Rik	chr15:89071096-89075924	-	Trabd (139)	no	0.8445	2.4864	2.8919	0.6549	2.69948	0.260296
BC090627	chr15:89416404-89429927	-	Chkb (0)	yes	1.5962	2.4313	0.259	0	38.0263	14.3937
Lnc363	chr15:89497936-89499457	+	Shank3 (1920)	yes	3.939	1.6269	0.4731	0	7.02621	0.409846
Lnc364	chr15:91245838-91248567	+	Slc2a13 (21852)	yes	0	0	1.7801	0	0	0
Lnc365	chr15:92344294-92366805	+	Cntn1 (2327)	no	1.273	2.589	2.0968	0.4443	0.160431	0.525023
Lnc366	chr15:93001614-93009225	+	Pdzrn4 (229795)	yes	1.1197	0.5658	1.2404	0	0	0.0921344
Lnc367	chr15:95788230-95790717	-	Ano6 (125)	no	0.1698	1.0473	0.2672	0.4209	0.520189	0.324722
E330033B04Rik	chr15:96264843-96285046	-	Arid2 (2374)	no	1.4332	0.6539	3.9286	1.4772	1.40419	1.5737
1700120C14Rik	chr15:99249261-99262043	+	Mcrs1 (0)	yes	4.2299	2.3636	17.8445	0.2248	3.64882	1.35537
Lnc368	chr15:100348566-100350583	+	Mettl7a2 (4633)	yes	0	0	1.1015	0.0298	52.5377	0
Lnc369	chr15:100640980-100669535	-	Cela1 (4886)	no	1.5065	1.4455	4.2631	0.5208	0.46916	26.3687
A330009N23Rik	chr15:101195214-101225186	-	Acvr1b (0)	no	0.5526	1.1292	2.5684	1.1309	1.36769	0.209734
6030408B16Rik	chr15:101293211-101297426	+	9430023L20Rik (2277)	yes	0.3279	0.4518	2.252	0.1347	0.333084	0.14786
Lnc370	chr15:102656987-102660445	+	Atp5g2 (5880)	yes	0.509	1.1589	4.6918	0.1909	0	0
Lnc371	chr16:4966578-4978374	+	Anks3 (2341)	no	3.1776	1.4223	1.298	4.6128	1.0823	0.366178
Lnc372	chr16:5431417-5451247	+	Fam86 (175461)	no	1.8595	0.7655	0.4969	0.2788	0	0
2610020C07Rik	chr16:11203382-11225796	+	Rsl1d1 (72)	no	0.6133	1.1528	0.5538	2.437	0.232888	3.05912
Lnc373	chr16:12704416-12711518	+	Ercc4 (404951)	yes	0	1.4618	0	0	0	10.7932
2310015D24Rik	chr16:13514130-13521114	-	Parn (16849)	yes	0.0262	0	1.3232	0	0	0
1300002E11Rik	chr16:21794346-21809039	+	Map3k13 (0)	no	8.7588	10.8511	19.8086	10.3175	11.6722	35.1214
Lnc374	chr16:24096380-24099812	+	Bcl6 (107768)	yes	0.0313	0	1.4084	0	0	0
1110054M08Rik	chr16:24392555-24393655	-	Lpp (306)	no	1.4823	1.4161	3.3391	1.2859	4.12827	1.44469
4632428C04Rik	chr16:30008624-30021430	+	Hes1 (56696)	yes	0.3951	0.3718	3.0991	0.0356	1.53978	1.97375
0610012G03Rik	chr16:31944385-31948521	-	Pigz (0)	no	25.4794	37.7237	33.6568	60.501	57.3584	33.2322
1700007L15Rik	chr16:33379402-33380736	-	Zfp148 (38)	no	3.3911	3.8212	1.8789	0.6587	1.41802	2.53923
E130310I04Rik	chr16:34943174-34958994	-	Mylk (0)	yes	1.3156	0.4102	0.5776	0.0486	3.52761	0.164806
Gm19522	chr16:42884369-42912070	-	Zbtb20 (5104)	yes	0	0.0292	6.6326	0.107	0.281885	0.0902895
5330426P16Rik	chr16:50689471-50732773	-	Ccdc54 (141619)	no	6.7463	4.2374	4.5886	3.0967	2.95925	0.892439
2310061J03Rik	chr16:55972423-55974617	-	Rpl24 Zbtb11 (0)	no	1.34	1.4819	0.9027	2.2966	2.57661	1.21432
Lnc375	chr16:56914295-56928025	-	Tmem45a (41862)	yes	1.4971	0.9325	0	0	0	0
Lnc376	chr16:61377069-61379707	+	Epha6 (771538)	no	6.4613	10.2811	0.3825	5.0121	0	0
Lnc377	chr16:72027687-72308665	+	Robo1 (535920)	no	0.6512	6.1511	0.7145	5.8446	0	0.173262
2810055G20Rik	chr16:77329327-77708737	+	Usp25 (212146)	no	12.6175	4.8	9.2971	0.8222	0.997627	0.570167
Lnc378	chr16:77764853-77787017	+	Cxadr (536635)	yes	0.3087	0.0501	1.5288	0.0537	0	0
E330011O21Rik	chr16:78250752-78275266	-	Cxadr (26222)	no	0.5463	0.4864	4.963	0.5144	0.507311	0.51591
Lnc379	chr16:85550765-85554160	-	Cyyr1 (3643)	no	3.7053	7.1727	4.1566	2.4604	0	0
Lnc380	chr16:90727520-90729197	+	2610039C10Rik (149)	no	1.1732	1.0148	0.2672	0.4409	0.543096	0.869801
Lnc381	chr16:94721671-94754652	+	Kcnj6 (0)	no	14.8823	16.5256	1.457	1.2528	0	0.144125
A630089N07Rik	chr16:98059857-98082439	-	Zfp295 (119818)	no	0.8817	1.3808	1.4428	0.8949	1.43124	3.77796
Lnc382	chr17:3103847-3114890	-	Scaf8 (81)	no	8.736	9.0718	3.6005	14.3693	4.88156	18.2065
1700102H20Rik	chr17:3557545-3559923	+	Tfb1m (0)	no	0.6467	1.6013	1.6506	1.0731	1.03536	0.581353
Lnc383	chr17:4908428-4911844	+	Arid1b (85823)	no	0.3659	1.0002	0.4191	0.5878	1.41021	0.343488
Lnc384	chr17:6456292-6477104	-	Dynlt1b (40809)	no	26.7048	20.7001	11.3106	121.236	8.11773	9.72896
Lnc385	chr17:10142904-10146174	-	Qk (41207)	yes	2.6499	0.2695	0.215	0	0	0
B930003M22Rik	chr17:10319918-10322263	+	Qk (557)	no	1.7489	1.5405	2.3097	6.8501	1.54838	1.96565
4732491K20Rik	chr17:12318852-12336760	+	Map3k4 (59)	no	1.3779	1.802	6.1288	1.376	1.35008	0.410272
Airn	chr17:12741310-12881285	+	Mas1 Igf2r (0)	no	48.6422	31.0378	35.522	2.2861	7.14462	7.33501
Lnc386	chr17:13035270-13040085	+	Sod2 (6050)	no	1.7763	0.3252	0.6592	6.8459	1.14279	0.345099
Lnc387	chr17:13673216-13683508	-	Tcte2 (32927)	no	0.9959	0.6327	2.4788	0.7729	0.638036	0.460414
LOC106740	chr17:14947735-14948823	-	Phf10 (0)	no	11.3493	15.565	12.0727	25.5077	18.9161	136.022
Lnc388	chr17:21527939-21532033	+	Zfp52 (7374)	no	0.8357	0.7887	1.1494	0.6815	0.300803	0.145642
Lnc389	chr17:21679906-21687922	+	3110052M02Rik (14940)	no	1.3412	1.5733	0.776	2.1728	0.639558	1.72904
Lnc390	chr17:22285902-22291729	-	Gm4944 (0)	no	5.744	5.8195	5.9844	4.2136	0.393305	3.73971

9530082P21Rik	chr17:23749235-23754065	+	Kremen2 (3406)	no	0.2033	0.0937	2.429	21.0114	13.4129	2.0078
D330041H03Rik	chr17:24409480-24414513	-	Abca3 (0)	no	1.3492	0.3628	0.4116	1.1026	78.6657	15.1859
2610019E17Rik	chr17:24528250-24530083	+	Traf7 (312)	no	11.3939	27.3124	7.1086	67.4642	12.6256	127.316
2810468N07Rik	chr17:25570806-25575043	+	Sox8 (120)	no	5.2362	2.9726	1.0041	4.7165	0.277321	0.415789
Lnc391	chr17:26460905-26465174	-	Neurl1b (18357)	yes	0.0377	0.047	1.8231	0	0.431282	0
Lnc392	chr17:26845334-26887670	+	Nkx2-5 (300)	yes	3.2539	1.7645	1.4729	0.0494	0.153328	0.179672
Lnc393	chr17:26944678-26962241	+	Cuta (5200)	no	6.2238	3.2508	1.462	37.9657	4.12595	2.25526
Lnc394	chr17:26962537-26967062	+	Zbtb9 (10641)	no	1.5076	2.205	1.0647	26.1773	2.41823	2.61612
Lnc395	chr17:26967187-26971180	+	Zbtb9 (5991)	no	1.734	1.447	0.4654	34.5125	5.87005	1.97077
Gm16197	chr17:29055515-29078961	-	Cdkn1a (12024)	yes	1.2944	0.6359	0.1721	0.0396	0.0422974	0.0852567
Lnc396	chr17:29439304-29466071	+	Pim1 (51503)	yes	0.1274	3.648	1.1376	0	0	0
Lnc397	chr17:29745285-29746015	-	1110021J02Rik (28998)	yes	5.0106	0	0	0	0.886026	0
Lnc398	chr17:31907876-31909241	-	Hsf2bp (35527)	yes	0.0827	0.0451	3.5317	0	8.56867	0
BC051226	chr17:33908099-33909312	-	Daxx (119)	no	1.6394	2.067	1.8203	13.3802	4.71609	9.06654
AA388235	chr17:33981491-33985358	+	Vps52 (14112)	no	0.0244	0.9543	1.0784	5.7233	0.558668	0.376392
1110038B12Rik	chr17:34950235-34952645	-	Hspa1b (3783)	no	56.2353	37.2638	3.0598	41.8354	10.7246	99.2807
A930015D03Rik	chr17:35994503-36117074	+	Gnl1 (5041)	no	3.4782	0.3353	0.5157	3.7484	3.91187	2.66642
Lnc399	chr17:44042859-44055651	+	Rcan2 (3343)	yes	0.8009	0.0875	1.026	0.056	0.0357462	0
Gm11110	chr17:57091597-57105942	-	Tnfsf9 (558)	no	0.7579	1.5612	0.5306	2.0543	0	0.552675
Lnc400	chr17:65097366-65101384	-	Tmem232 (154620)	yes	0.5744	0.2674	1.1075	0	0	0
Lnc401	chr17:67615172-67626695	-	Lrrc30 (4269)	yes	0.1131	0.1301	1.3762	0	0	0
C030034I22Rik	chr17:69416446-69419192	+	A330050F15Rik (22879)	no	0.4848	0.6887	1.066	3.3451	1.01569	2.68291
Lnc402	chr17:69423808-69439311	-	A330050F15Rik (14)	yes	1.5995	0.4535	0.31	0.2068	0	0
Trmt61b	chr17:71557026-71598819	-	Spdya (0)	no	4.2346	5.195	4.3822	11.2555	3.9154	12.3172
Lnc403	chr17:73020126-73022343	-	Lbh (76173)	yes	0.3779	1.0267	0.6044	0	0	0
Lnc404	chr17:73054898-73084103	-	Lclat1 (23881)	yes	0.8177	4.4741	0.7857	0.0909	0	0
Lnc405	chr17:79438860-79451722	-	Cdc42ep3 (96631)	yes	0.5337	1.3685	2.5031	0	0	0
1110020A21Rik	chr17:84917181-84957710	-	Ppm1b (970)	no	1.852	1.0786	1.5923	1.2738	31.9625	52.5872
Lnc406	chr17:87462309-87473933	-	Calm2 (26998)	yes	1.1076	1.6647	0.0154	0.1059	0	0.337966
Gm1976	chr17:94759941-94864810	-	LOC100044193 (107)	no	0.3054	1.7235	1.3329	8.3556	2.64165	6.73706
4833419F23Rik	chr18:4352945-4368945	+	Map3k8 (0)	yes	3.4511	1.4767	3.5751	0	0.219381	0.176244
Lnc407	chr18:11043493-11053125	-	Gata6 (1278)	yes	7.5347	14.9546	8.5299	0	0.312033	1.4996
D830029L11	chr18:16816406-16822783	+	Cdh2 (7284)	no	1.8708	0.5674	2.1064	3.5444	0	0.102471
Lnc408	chr18:23750511-23752305	-	Mapre2 (27)	yes	2.4503	1.34	4.246	0.2452	1.60749	0.192613
2410004N09Rik	chr18:33794891-33799057	+	Epb4.1l4a (1435)	no	12.0486	20.8327	1.209	30.6959	13.2264	67.7791
Lnc409	chr18:34756364-34758569	+	Fam53c (2257)	no	1.2923	1.8514	0	0.283	0	11.8599
Lnc410	chr18:35037320-35058875	+	Ctnna1 (81591)	yes	0.061	0.0064	4.2196	0	0	0
Lnc411	chr18:35058186-35087606	-	Ctnna1 (31305)	yes	0.3782	0.6422	3.5706	0	1.3977	0
Snhg4	chr18:35553409-35562096	+	Matr3 (8748)	no	8.3699	7.9428	6.357	15.187	10.6597	88.0571
Vaultrc5	chr18:36801762-36802107	+	Zmat2 (2102)	no	1080.37	1073.22	1137.43	0.3267	1.18488	0
Lnc412	chr18:38119554-38121420	-	Pcdh1 (67324)	yes	0	0.0239	1.2885	0.0577	0.320528	0
1700086O06Rik	chr18:38238404-38250565	-	0610009O20Rik (323)	no	1.4085	2.4301	1.0843	6.2097	1.84143	11.9437
Lnc413	chr18:38601618-38837556	+	Spry4 (350)	no	2.1208	2.7036	0.4344	1.5243	0.401969	0.202563
Lnc414	chr18:45268870-45559579	+	Kcnn2 (291283)	no	5.9783	1.5849	4.8856	0.6172	0	0
Lnc415	chr18:46615772-46617792	+	Eif1a (2523)	yes	1.063	0.0724	0.2544	0.0861	0.132833	0.213008
Lnc416	chr18:53221447-53221872	-	Snx2 (1012)	no	80.416	107.444	1.0284	125.833	0	0
9430076G02Rik	chr18:54422294-54453294	+	Csnk1g3 (458868)	yes	2.276	0.5199	0.1105	0.0906	0	42.5321
Lnc417	chr18:56403520-56418266	-	Gramd3 (13865)	yes	4.3654	14.7013	1.9767	0	0	0
Lnc418	chr18:60439269-60444045	+	2010002N04Rik (34921)	yes	2.0927	0.2742	1.9979	0	0.219535	0.0276279
E330013P06	chr18:61644069-61666000	-	Il17b (21934)	no	1.5377	5.446	7.6306	2.3229	9.90341	1.8683
1500015A07Rik	chr18:61726389-61728253	+	Grpel2 (58)	no	1.4615	2.4554	2.109	6.1505	2.03654	4.57688
Lnc419	chr18:65552308-65580679	-	Zfp532 (450)	no	0.8674	1.6582	1.4541	1.5953	3.8224	3.01953
Lnc420	chr18:75358643-75367055	-	Smad7 (309)	no	1.9206	4.7497	3.6901	3.61	1.12042	1.24305
Lnc421	chr18:77728396-77733269	-	8030462N17Rik (18777)	yes	0.4697	1.0352	2.1598	0.1879	0.0786724	1.39152
Lnc422	chr18:83436854-83438960	+	Zfp516 (429809)	yes	1.4882	0.0878	0.0587	0.2197	0	0
4930481A15Rik	chr19:5406797-5422847	+	Drap1 (16026)	no	0.8024	2.8524	12.8995	1.912	8.76916	4.58567
Malat1	chr19:5792510-5803492	-	Scyl1 (32091)	no	2059.2	725.724	4284.75	243.949	567.692	183.715

Neat1	chr19:5823413-5845880	-	Frmd8 (5093)	no	8.2255	1.7066	328.956	3.5426	337.016	57.0525
Snhg1	chr19:8723243-8726473	+	Slc3a2 (0)	no	58.9154	65.8069	12.5652	68.8862	9.39664	415.71
5730408K05Rik	chr19:8887084-8888770	-	1810009A15Rik (83)	no	134.528	92.7639	21.6036	30.943	4.50764	73.6211
Lnc423	chr19:11375420-11393484	+	Ms4a4c (32240)	no	0.1038	0.2656	5.1362	0.7104	0.364852	2.08045
Gm5512_2	chr19:12905230-12906985	+	Olfr1447 (3452)	no	5.9137	9.6654	20.6337	8.0379	0	0.273098
E030003E18Rik	chr19:20492714-20556410	+	Aldh1a1 (0)	yes	0.0692	0.0595	2.8063	0.0485	3.3831	0.836075
Lnc424	chr19:27257481-27262361	-	Vldlr (6558)	yes	0.1848	0.0174	5.5444	0.059	0	0.0183789
Lnc425	chr19:28770782-28788201	+	Slc1a1 (64383)	yes	0	0	1.1417	0	0	0
Gm9895	chr19:29067300-29069503	+	Ak3 (19398)	no	0.9622	0.7175	1.6871	0.2791	2.72346	19.1857
8430431K14Rik	chr19:31212777-31221117	-	Prkg1 (0)	yes	2.6901	0.5272	2.514	0.1924	0.109192	0.150167
Lnc426	chr19:32104391-32105030	-	Asah2 (0)	yes	2.0865	0.1046	0.1538	0	0	0
2700046G09Rik	chr19:32389215-32392130	+	Sgms1 (0)	no	1.6767	3.6279	3.0522	1.2963	4.51127	1.25056
Lnc427	chr19:36217923-36264559	-	Pcgf5 (83962)	yes	0.4547	0.0738	4.4603	0	0	0
1500017E21Rik	chr19:36618530-36690099	-	Hectd2 (0)	no	5.4374	4.485	8.9754	0.481	0.272875	1.03466
Lnc428	chr19:40278628-40283312	+	Pdlim1 (6740)	yes	0.0594	0.1051	1.6208	0	0	0
Lnc429	chr19:41524923-41527512	+	Lcor (0)	yes	2.7605	0.1525	2.2798	0.0864	0	0.403191
Lnc430	chr19:43528437-43564592	+	Got1 (3820)	yes	0	0	1.5361	0	0	0
BC037704	chr19:43675177-43677170	+	Slc25a28 (296)	no	0.4651	0.5346	1.127	1.3769	0.638529	0.719923
Lnc431	chr19:53672999-53677099	-	Rbm20 (206)	yes	3.1945	3.9783	1.3733	0	0	0.0250434
Lnc432	chr19:56738740-56746739	-	Adrb1 (17881)	yes	0.1885	0.1705	5.9389	0.0296	0.062166	0
Lnc433	chr19:56743275-56762066	+	Adrb1 (14417)	yes	0.3127	0.0665	5.9899	0.1519	0.306587	0.00611916
2010204K13Rik	chrX:7411816-7429597	-	Gm14346 Gm10921 (0)	no	17.6885	15.1112	0.4456	10.272	9.83322	12.1307
Lnc434	chrX:11379663-11384038	-	Gm14346 Gm10921 (0)	yes	0	0	1.4576	0	0	0
Lnc435	chrX:11395887-11418362	-	Gm14346 Gm10921 (0)	yes	0.3344	0.0903	1.5052	0	0	0
2900008C10Rik	chrX:12134453-12160346	-	Gm14346 Bcor Gm10921 (0)	no	3.1108	2.6828	5.0559	2.0203	2.54501	6.98976
A230072C01Rik	chrX:20961675-20987453	+	Gm14346 Gm10921 Uxt (0)	no	1.8564	2.6563	2.7576	8.0946	4.61672	4.49765
6720401G13Rik	chrX:50555743-50635310	-	2610018G03Rik (205785)	no	2.798	4.7493	6.1962	31.0436	15.9768	10.2975
C430049B03Rik	chrX:53046194-53057196	-	Plac1 (12805)	no	40.7283	68.9126	3.191	1.6262	0.385657	1.94036
Ncrna00086	chrX:56374585-56379130	+	Zfp449 (8911)	no	1.247	1.2553	0.0651	6.3535	0.700159	0.212891
4933407K13Rik	chrX:75723976-75764699	-	Pls3 (20954)	no	1.0649	0.8294	0.8545	2.0388	0.403967	1.31659
Gm14827	chrX:94442249-94447727	+	Apoo (21149)	no	0.4048	0.2016	1.0742	3.1496	0.0723134	0.350494
F630028O10Rik	chrX:96239815-96244300	+	Vsig4 (7387)	no	0.4316	1.8215	9.6168	0.3031	0.126151	22.7252
Lnc436	chrX:101457751-101463733	+	Nono (4210)	yes	1.093	0.7888	1.2476	0.1978	0	0.29543
Lnc437	chrX:101463828-101465404	+	Nono (10287)	yes	0.6318	0.7874	1.3684	0.1241	0	0.324378
Xist	chrX:103460372-103483233	-	Tsx (58650)	no	18.6479	0.1376	0.1303	58.4113	25.36	71.9822
Enox	chrX:103493575-103530932	+	Tsx (68992)	no	0.9995	0.3998	1.164	2.4083	2.22474	4.74581
Lnc438	chrX:103524629-103530932	+	Tsx (100046)	no	3.2597	2.207	2.8578	8.0113	2.22474	4.74581
B230206F22Rik	chrX:103560909-103623754	-	Zcchc13 (6831)	no	3.4154	2.4026	2.72	8.0519	3.50585	8.05177
5530601H04Rik	chrX:105038743-105070124	-	2610029G23Rik (9608)	no	2.5174	2.0199	1.201	4.4568	3.47658	14.3836
Lnc439	chrX:105103596-105116594	+	2610029G23Rik (0)	no	1.4097	0.3601	0.3805	0.7398	0.887836	1.04532
Lnc440	chrX:105725006-105751097	-	Fgf16 (13379)	yes	0.1122	0.1964	1.2112	0	0	0.188932
2810403D21Rik	chrX:108834477-108887440	+	Brwd3 (122)	no	0.8925	1.3332	1.1397	1.282	0	2.365
Lnc441	chrX:111898562-112092531	+	Tex16 (194957)	yes	2.0074	0.5638	0	0	0	0.103528
Lnc442	chrX:135979856-135982414	+	Arxes2 (13963)	no	1.0128	0.132	0	2.0614	0.310724	0.272223
BC065397	chrX:136742258-136838180	+	Morf4l2 (0)	no	2.2042	1.6797	1.8751	3.5828	4.27261	2.36678
2900056M20Rik	chrX:152294827-152327493	-	Tspyl2 (9358)	no	3.0333	0.6319	2.8058	15.5136	2.45576	3.79609
Lnc443	chrX:169320489-169368861	+	Hccs (117)	no	1.6788	1.2102	0.8768	12.5092	0	1.33929
G530011O06Rik	chrX:169973872-169988869	-	Mid1 (0)	yes	0.1078	0.2583	4.2971	0	1.86498	0.617042
