# Supplementary material for: Important cardiac transcription factor genes are accompanied by bidirectional long non-coding RNAs
Source: BMC Genomics. 2018 Dec 27;19:967. doi: 10.1186/s12864-018-5233-5 (PMC6307297; doi:10.1186/s12864-018-5233-5)
Supplement: Supplementary file 3 — Table S2. List of antisense lncRNA candidates and their corresponding protein-coding genes. (PDF 17 kb) [file 12864_2018_5233_MOESM3_ESM.pdf]

antisense lncRNA candidate	corresponding mRNA
1110034B05Rik	9430016H08Rik
2900060B14Rik	Clasp1
2900009J06Rik	Acmsd
Gm19461	Plekha6
2810025M15Rik	Rasal2
Dnm3os	Dnm3
Gm10516	Rcor3
A330023F24Rik	Cd46
Snhg7	Fam69b
D330023K18Rik	Gpr107
Gm13446	Dab2ip
1700011J10Rik	Sp3
Lnc55	Cdan1
BC029722	Mmp24
5430405H02Rik	Tgif2
Lnc63	Sall4
Lnc65	Ctcfl
4930429B21Rik	Zmat3
1700113A16Rik	Mef2d
Gm15417	Zbtb7b
4933431E20Rik	Gstm5
A930005H10Rik	Dph5
Snhg8	Prss12
2510003D18Rik	Asb17
2010003O02Rik	Topors
C530005A16Rik	Gpbp1l1
A630072M18Rik	Rsbn1l
Gm9899	Kcnk3
Lnc125	Tbx5
A930024E05Rik	Kdm2b
Gm20605	Irs3
6330418K02Rik	BC037034
9330158H04Rik	Chrm2
4930414L22Rik	Mat2a
2610306M01Rik	Aak1
9530026P05Rik	Adamts9
Gt(ROSA)26Sor	Thumpd3
9330179D12Rik	Ccnd2
Gm10069	Pzp
2810454H06Rik	Gpr19
Lnc152	Emp1
Gm15545	Bcl2l12

A230056P14Rik	Nipa1
B130024G19Rik	Nr2f2
2900076A07Rik	Fsd2
Gm14492	Lsp1
R74862	Cd81
Kcnq1ot1	Kcnq1
Gm16793	Mfhas1
2010320M18Rik	Mast3
Gm10638	Siah1a
9330175E14Rik	Nlrc5
4930513N10Rik	Cnot1
2310014F07Rik	1600029D21Rik
9430037G07Rik	Trim43c
9330159M07Rik	Trim43c
B430319G15Rik	Plod2
E530011L22Rik	Nktr
A630066F11Rik	Pcmt1
BC020402	Nup43
D830005E20Rik	Trdn
E130307A14Rik	Traf3ip2
E130317F20Rik	Ptbp1
2310050B05Rik	Itgb1bp3
1700012D01Rik	Nab2
2210015D19Rik	Urgcp
4933439C10Rik	Zkscan17
2410006H16Rik	BC046404
AA465934	Pex12
D030028A08Rik	Sp2
LOC100503496	Slc16a6
1810032O08Rik	St6galnac2
1700012B15Rik	Rab10
1110002L01Rik	Kif3c
9330151L19Rik	1110034A24Rik
3110056K07Rik	Psma3
Lnc305	Cpsf2
Rian	Rtl1
Etohd2	Zcchc6
1810034E14Rik	Cdc14b
A830082K12Rik	Nr2f1
4833422C13Rik	Ssbp2
3110070M22Rik	Gm7120
1810062O18Rik	Ppp3cb
6230400D17Rik	Chchd1

Lnc338	3425401B19Rik
Gm16973	Mphosph8
Dleu2	Kcnrg
Lnc353	Slitrk5
BC037032	Oxct1
2310069G16Rik	Sybu
9930014A18Rik	Fam84b
D730005E14Rik	Apobec3
1700120C14Rik	Mcrs1
A330009N23Rik	Acvr1b
2610020C07Rik	Gspt1
1110054M08Rik	Lpp
0610012G03Rik	Pigz
E130310I04Rik	Mylk
Gm19522	Zbtb20
2310061J03Rik	Zbtb11
Lnc381	Kcnj6
1700102H20Rik	Tfb1m
Airn	Mas1
9530082P21Rik	Flywch1
D330041H03Rik	Abca3
2610019E17Rik	Rab26
A930015D03Rik	Gm11127
Gm11110	Tnfsf9
Trmt61b	Spdya
1110020A21Rik	Ppm1b
4833419F23Rik	Map3k8
Lnc407	Gata6
2410004N09Rik	Epb4.1l4a
1700086O06Rik	0610009O20Rik
Lnc419	Zfp532
4930481A15Rik	Drap1
Snhg1	Slc3a2
2700046G09Rik	Sgms1
1500017E21Rik	Hectd2
A230072C01Rik	Uxt
BC065397	Glra4
G530011O06Rik	Mid1
