# Supplementary material for: Important cardiac transcription factor genes are accompanied by bidirectional long non-coding RNAs
Source: BMC Genomics. 2018 Dec 27;19:967. doi: 10.1186/s12864-018-5233-5 (PMC6307297; doi:10.1186/s12864-018-5233-5)
Supplement: Supplementary file 4 — Table S3. List of bidirectional lncRNA candidates and their corresponding protein-coding genes. (PDF 21 kb) [file 12864_2018_5233_MOESM4_ESM.pdf]

bidirectional lncRNA candidate	corresponding mRNA
2810408I11Rik	Ccnyl1
Apol7d	Fn1
2900009J06Rik	Ccnt2
Lnc18	Ivns1abp
Gas5	Zbtb37
Lnc24	Fam36a
Lnc27	Rrp15
Lnc28	G0s2
Lnc29	Cr1l
4921530L18Rik	Stam
Gm13375	Arhgap21
1700084E18Rik	Lrrc8a
D330023K18Rik	Gpr107
Gm13483	Mmadhc
Lnc43	Grb14
1700011J10Rik	Sp3
Lnc46	Gpr155
Lnc47	Slc39a13
Lnc49	Pacsin3
2810002D19Rik	Ttc17
Lnc52	Eif3m
C130080G10Rik	Actc1
Lnc59	Fkbp1a
2500004C02Rik	Asxl1
5430405H02Rik	1110008F13Rik
Lnc63	Sall4
4921531C22Rik	Taf4a
Lnc70	Tbl1xr1
4930429B21Rik	Zmat3
Lnc78	Ssr3
Gm15417	Zbtb7b
Lnc84	Slc25a24
A930005H10Rik	Dph5
Lnc86	F3
Lnc93	Cyr61
1700123M08Rik	Pdp1
4933421O10Rik	Ube2j1
C630043F03Rik	Tle1
Lnc100	Nfib
Lnc104	Ttc39a
Btbd19	Tctex1d4
2610204G22Rik	Atad3a
Gm10560	Tnfrsf18
AW011738	Isg15
Lnc115	Magi2
5031425E22Rik	Mll5
2900005J15Rik	Prkag2
4831440E17Rik	Mll3
9230114K14Rik	Dhx15
Gm9958	Ankrd17
Lnc125	Tbx5
A930024E05Rik	Kdm2b

6330418K02Rik	BC037034
0610040B10Rik	Zdhhc4
Gm16039	Rpa3
9330158H04Rik	Chrm2
Lnc141	Creb3l2
1600020E01Rik	Pcbp1
2610306M01Rik	Aak1
Gm5577	H1fx
Gt(ROSA)26Sor	Setd5
3110021A11Rik	Erc1
5930416I19Rik	Foxm1
Gm10069	Fkbp4
2810454H06Rik	Gpr19
Gm15706	Kras
Lnc154	Cacng6
Lnc156	Dact3
Lnc161	Zfp568
Gm15545	Prmt1
A230056P14Rik	Nipa2
2310010J17Rik	Picalm
4632427E13Rik	Rab30
Gm16675	Irf2
Lnc198	Hand2
Lnc203	Ddx60
2010320M18Rik	Pik3r2
Gm4890	Mmaa
Lnc209	Cd97
4933436C20Rik	Irx5
Lnc212	Gins2
9330133O14Rik	Mvd
2810013P06Rik	Ankrd11
Lnc215	Tbx20
Lnc220	Scaper
Lnc222	Myzap
2310009A05Rik	Pigb
E530011L22Rik	Zfp651
Lnc233	Cnksr3
BC020402	Lats1
D830005E20Rik	Trdn
E130307A14Rik	Rev3l
Gm17769	Adarb1
E130317F20Rik	Ptbp1
Lnc242	Scyl2
4932415G12Rik	Ccdc41
Lnc247	Kcnmb4
4933412E12Rik	Rab3ip
Lnc248	Irak3
1700012D01Rik	Nab2
8430429K09Rik	Rnf185
Tug1	Morc2a
Lnc250	Grb10
Lnc254	Etaa1
Lnc259	Sap30l

Lnc260	Hand1
2610507I01Rik	Mrpl55
4933439C10Rik	Zkscan17
1700013G23Rik	Med9
Gm16516	Map2k3
2810001G20Rik	Cox10
Lnc265	Lig3
Lnc266	Unc45b
Lnc268	Ggnbp2
C030037D09Rik	Msi2
Lnc272	Igf2bp1
D030028A08Rik	Pnpo
Lnc274	Mrc2
0610009L18Rik	Actg1
1110002L01Rik	Asxl2
Lnc286	Ywhaq
Lnc287	Klf11
Lnc291	Meox2
Lnc296	Hectd1
3110056K07Rik	Arid4a
Lnc305	Cpsf2
Snhg10	Glrx5
Lnc308	Dync1h1
2810029C07Rik	Mark3
D430020J02Rik	Ncapg2
Lnc317	Rala
Lnc319	Atxn1
C030044B11Rik	Fam120a
Etohd2	Isca1
1810034E14Rik	Cdc14b
Lnc324	Papd7
Gm20554	Irx2
Lnc326	Tppp
A830082K12Rik	Nr2f1
Gm9776	Ap3b1
Lnc329	Mtap1b
3110070M22Rik	Gm7120
Lnc336	Camk2g
2010107H07Rik	Nt5dc2
Lnc338	3425401B19Rik
Lnc350	Chmp7
Gm4285	Tpt1
Lnc353	Slitrk5
0610007N19Rik	Sema5a
9930014A18Rik	Fam84b
Gm16576	Sun2
1810021B22Rik	Trabd
Lnc367	Ano6
E330033B04Rik	Arid2
A330009N23Rik	Grasp
Lnc371	Anks3
2610020C07Rik	Rsl1d1
1110054M08Rik	Lpp

0610012G03Rik	Ncbp2
1700007L15Rik	Zfp148
2310061J03Rik	Zbtb11
Lnc380	2610039C10Rik
Lnc382	Scaf8
1700102H20Rik	Tfb1m
B930003M22Rik	Qk
4732491K20Rik	Map3k4
D330041H03Rik	Rnps1
2610019E17Rik	Traf7
2810468N07Rik	Sox8
Lnc392	Nkx2-5
BC051226	Daxx
Lnc402	A330050F15Rik
1110020A21Rik	Ppm1b
Gm1976	LOC100044193
4833419F23Rik	Map3k8
Lnc407	Gata6
Lnc408	Mapre2
1700086O06Rik	0610009O20Rik
Lnc413	Spry4
1500015A07Rik	Grpel2
Lnc419	Zfp532
Lnc420	Smad7
Snhg1	Slc3a2
5730408K05Rik	1810009A15Rik
2700046G09Rik	Sgms1
BC037704	Slc25a28
Lnc431	Rbm20
A230072C01Rik	Uxt
2810403D21Rik	Brwd3
BC065397	Morf4l2
Lnc443	Hccs
G530011O06Rik	Mid1
