# Supplementary material for: Important cardiac transcription factor genes are accompanied by bidirectional long non-coding RNAs
Source: BMC Genomics. 2018 Dec 27;19:967. doi: 10.1186/s12864-018-5233-5 (PMC6307297; doi:10.1186/s12864-018-5233-5)
Supplement: Supplementary file 6 — Table S4. List of bidirectional lncRNA candidates that were identified from the analysis of the NCBI RefSeq database (GRCm38.p3). (PDF 148 kb) [file 12864_2018_5233_MOESM6_ESM.pdf]

Protein-coding gene	Corresponding bidirectional lncRNA
Rb1cc1	4732440D04Rik
Cops5	Cspp1
Msc	LOC105243866
Rpl7	Rdh10
Tceb1	D030040B21Rik
Crispld1	Gm16070
Paqr8	6720483E21Rik
Pih1d3	4931428L18Rik
Prim2	1700001G17Rik
Hs6st1	LOC105243903
Uggt1	LOC105243904
Cnnm4	Gm33454
Rev1	Gm5099
Aff3	Gm34596
Il1r2	Gm16894
Pou3f3	Pantr1
Gpr45	LOC105243914
Gulp1	LOC105243918
Dnah7a	Gm32311
Satb2	9130024F11Rik
1700066M21Rik	4930558J18Rik
Bzw1	LOC105243929
Orc2	Gm15834
Trak2	Stradb
Als2	Gm33667
Gm973	Gm28411
Wdr12	Carf
Ino80d	Ino80dos
Zdbf2	Gm11608
Klf7	Gm35880
Ccnyl1	2810408I11Rik
Fn1	Apol7d
Tnp1	1700027A15Rik
Tns1	LOC105243958
Usp37	Rqcd1
Usp37	Plcd4
Atg9a	Ankzf1
Glb1l	Stk16
Speg	Gm15179
Pax3	Gm33152
Cul3	LOC105243961
Mff	LOC105243963
Slc16a14	LOC105243965
C130026I21Rik	LOC105243966
Sp110	Sp140
Gpr55	LOC105246647
B3gnt7	Gm16341
Ncl	C130036L24Rik
Alppl2	Gm29371
Iqca	4930434B07Rik
Per2	Gm35591
Hdac4	LOC105246664
Rnpepl1	5033417F24Rik
Gpr35	9430060I03Rik
Mterf4	LOC105246665
Pask	Ppp1r7
Slco4c1	Panct2
Cntnap5b	LOC105246671
Rnf152	Gm17634
Pign	2310035C23Rik
Phlpp1	Gm20753
Cfap221	Gm33817
Sctr	Gm33764
LOC105246689	LOC105246690
Ccdc93	Gm35243

Dpp10	Gm35355
Ccnt2	LOC105246699
Ccnt2	2900009J06Rik
Daf2	Gm15674
Pigr	LOC102640771
Rassf5	Gm29953
Rbbp5	LOC102633047
Mdm4	LOC105246708
Pik3c2b	Gm28609
Lmod1	LOC105246712
Nav1	Gm32940
Nav1	Gm32835
Csrp1	Gm33139
Kif21b	Gm15850
5730559C18Rik	Gm3878
Crb1	4933436E23Rik
Trove2	Uchl5
Brinp3	Gm29514
Ptgs2	Ptgs2os
Edem3	LOC102640772
Rgs16	C230024C17Rik
Glul	LOC102631806
BC034090	Gm31102
Soat1	LOC105243983
Ralgps2	LOC105243985
Rasal2	2810025M15Rik
4930523C07Rik	LOC105243991
Zbtb37	Gas5
Dars2	Cenpl
Dnm3	Gm31985
Fmo1	Gm32197
Atp1b1	Gm32391
Dcaf6	Mpc2
Fam78b	Gm16701
Uck2	LOC102636733
Nuf2	Gm34624
Hsd17b7	3110045C21Rik
Fcgr2b	LOC102638466
Dedd	LOC105244007
F11r	Gm26641
Cd244	Gm35200
Slamf6	LOC105244010
Pea15a	Gm17224
Slamf9	Gm35473
Igsf9	Gm35522
Dusp23	LOC102641637
Spta1	Gm36196
Opn3	LOC105244015
Exo1	Gm36360
1700016C15Rik	Gm30115
Psen2	Gm31728
Parp1	Gm31986
Wdr26	LOC102643210
Srp9	Gm33725
Brox	Aida
March2	Gm34451
Mark1	LOC105244044
Rrp15	A430105J06Rik
Spata17	Gpatch2
Prox1	LOC102632463
Dtl	Ints7
Rcor3	Gm30956
G0s2	Gm31467
Cr1l	Gm32313
H60c	Gm29826
Ppp1r14c	Gm26752

Rmnd1	1700052N19Rik
Lats1	BC020402
Ppil4	LOC102632778
Grm1	4930567K20Rik
Aig1	Gm32172
Vta1	1700016L04Rik
Txlnb	Gm20655
Map7	4930405J17Rik
Aldh8a1	Gm33803
LOC102638890	Ctgf
Arg1	Gm36172
Akap7	LOC105245123
Smlr1	Gm29571
Tmem200a	Gm9767
Tpd52l1	Gm30730
Tspyl4	Gm26564
Marcks	Gm16524
Fam229b	Tube1
Fyn	Gm31616
Rev3l	E130307A14Rik
Smpd2	Ppil6
Foxo3	Gm33333
Nr2e1	LOC102636808
Sec63	Gm15199
Sobp	9030612E09Rik
Qrsl1	Rtn4ip1
Atg5	LOC105245144
Sim1	LOC105245152
Man1a	Gm16998
Hsf2	4930467K11Rik
Gcc2	Gm9919
Sar1a	1700022H01Rik
Hk1	Hk1os
Hk1	LOC105245188
Hkdc1	Gm30322
Pbld1	Gm16143
Pbld1	LOC102641989
Ctnna3	Gm30644
Egr2	Gm32255
Rhobtb1	Gm33263
Rhobtb1	A930033H14Rik
Ube2d1	Gm34376
Cisd1	Ipmk
Rsph14	Rab36
Lrrc75b	Ggt1
Mif	LOC105245213
Gm867	Vpreb3
Gm5134	LOC105245214
Dip2a	LOC105245215
Spatc1l	4930483K19Rik
Pcbp3	Gm35664
Slc19a1	Gm3137
Pofut2	Gm10941
Adarb1	Gm17769
Lrrc3	Gm29914
Trappc10	LOC105245224
Agpat3	LOC105245225
Syde1	Gm30346
R3hdm4	Kiss1r
Arid3a	LOC105245231
Dos	Atp5d
Apc2	Gm30682
Pcsk4	Reep6
Mex3d	Gm30823
Klf16	Gm26710
Csnk1g2	Gm31298

Mob3a	Izumo4
Plekhj1	Sf3a2
Lsm7	Sppl2b
Mrpl54	Apba3
Tjp3	Pip5k1c
Nfic	Gm31764
LOC102635990	Gm4924
Txnrd1	LOC102641476
Slc41a2	LOC105245243
Hsp90b1	BC030307
Pmch	Gm30233
Dram1	LOC105245256
Gnptab	LOC105245255
Scyl2	1500026H17Rik
Uhrf1bp1l	Gm30963
Cfap54	Gm32468
Tmcc3	LOC105245265
Cep83	Cep83os
Socs2	5730420D15Rik
Tmtc2	Gm15663
Mettl25	Ccdc59
Myf6	Gm36177
Ppp1r12a	LOC105245277
Pawr	LOC105245278
Cnot2	5330438D12Rik
Rab3ip	4933412E12Rik
Dyrk2	LOC105245302
Irak3	LOC102637719
Lemd3	Gm35235
Ppm1h	Gm36284
Usp15	Gm4129
Ctdsp2	LOC105245323
Tspan31	A730063M14Rik
Dtx3	F420014N23Rik
Kif5a	LOC105245325
Mbd6	Gm20492
Mbd6	Ddit3
Nab2	1700012D01Rik
Tmem194	Gm16230
Tac2	LOC105245329
Timeless	Gm26847
Nabp2	Rnf41
Myl6	A430046D13Rik
Myl6b	A430046D13Rik
Ikzf4	Gm26876
Dgka	Gm31504
Rnf185	8430429K09Rik
Smtn	Gm11946
Smtn	Gm33983
Morc2a	Tug1
Cabp7	Gm35102
Ewsr1	Rhbdd3
Emid1	LOC105243808
Ankrd36	LOC105243814
Gck	Gm11967
Ccm2	LOC105243821
Igfbp3	LOC105245341
Ikzf1	Gm11999
Pom121l12	1700046C09Rik
Fbxo48	Eldr
Etaa1	LOC102633673
LOC105245354	Gm32517
Efemp1	Gm34185
Sptbn1	Gm34635
Gabrb2	Gm34591
Pwwp2a	Gm34963

Sox30	Gm35497
Gnb2l1	Gm36109
LOC105244158	LOC105243624
Cnot6	Gm36411
Mgat4b	Gm36556
Gm12569	5133400J02Rik
N4bp3	D930048N14Rik
Ube2b	Cdkl3
Shroom1	Gm30585
Kif3a	A430108G06Rik
Slc22a21	Gm30927
Glra1	LOC105244170
Fam114a2	Mfap3
Sap30l	2010001A14Rik
Gm32125	Gm32915
Cnot8	Gm12248
Irgm2	Gm33397
Zfp39	LOC105246870
Trim11	LOC105244174
Mrpl55	2610507I01Rik
Prss38	Gm15755
Snap47	Jmjd4
Zkscan17	4933439C10Rik
Flcn	Gm16062
Med9	Med9os
Natd1	LOC105244177
Map2k3	Map2k3os
B9d1	B9d1os
Zswim7	Ttc19
Ubb	Gm12279
Cdrt4	Cdrt4os2
Cox10	2810001G20Rik
Myocd	Gm30586
Dnah9	LOC102633054
Shisa6	LOC102642924
Myh4	Gm12300
Pik3r5	Gm32046
Odf4	LOC105246876
Arhgef15	Gm32273
Chd3	Gm32483
Senp3	Tnfsf13os
Fgf11	G630025P09Rik
Tnk1	Gm32708
2810408A11Rik	Neurl4
Gabarap	Gm32862
0610010K14Rik	LOC105244601
Inca1	Kif1c
Nup88	Rpain
4933427D14Rik	Txndc17
Slc13a5	1700051A21Rik
Tekt1	Gm34317
Ube2g1	Gm34592
Ankfy1	Gm34530
P2rx1	LOC105244606
Camkk1	4732414G09Rik
P2rx5	Gm35001
LOC105244610	Mettl16
Sgsm2	Tsr1
Serpinf1	Gm35337
Nxn	LOC105244613
Pipox	Gm29979
Sez6	Gm11190
Phf12	Gm30112
Dhrs13	Dhrs13os
Spag5	Gm3948
Rab11fip4	Gm30718

Spaca3	4930507D10Rik
Tmem132e	LOC102641825
Cct6b	Zfp830
Lig3	Gm11423
Rad51d	Fndc8
Hnf1b	Gm12576
Lhx1	Lhx1os
Dhrs11	4930502E09Rik
Appbp2	Appbp2os
Tbx2	2610027K06Rik
Cltc	LOC105246880
Ppm1e	Gm33937
Rad51c	Tex14
Sept4	Gm34000
Msi2	C030037D09Rik
Akap1	4930556N13Rik
Trim25	LOC102637854
Dgke	Dgkeos
Stxbp4	Cox11
Utp18	Mbtd1
Nme1	Gm35198
Kat7	LOC102640811
Hoxb5os	Hoxb3
Hoxb2	LOC102632302
Skap1	Gm11529
Prr15l	Gm11525
Pnpo	D030028A08Rik
Sp2	Gm30809
Npepps	Gm11592
Cdk12	LOC105242875
Stard3	1700003D09Rik
Nr1d1	Gm31862
Krt25	Gm32502
Krtap2-4	LOC105246886
Krt31	Gm11571
Jup	Gm12348
Zfp385c	Dhx58os
Ptges3l	Rundc1
Brca1	Nbr1
Etv4	LOC105243483
Ccdc43	Gm34392
Gfap	Gm34560
1700023F06Rik	Gm34879
Spata32	Gm34823
Arhgap27	Arhgap27os3
Lyzl6	1700072I22Rik
Gosr2	C130046K22Rik
Tlk2	1700052K11Rik
Mrc2	LOC105243515
Pecam1	Milr1
Ddx5	Cep95
1810010H24Rik	Gm36273
Nol11	LOC105243532
Helz	LOC105243533
Rgs9	LOC105243550
Wipi1	Gm29875
Fam20a	LOC105243553
Abca5	LOC105243556
Map2k6	LOC105243557
Fam104a	D11Wsu47e
Ict1	Gm32013
Atp5h	Kctd2
Slc16a5	Gm11695
Caskin2	Tsen54
Exoc7	Gm26730
Jmjd6	Mettl23

Sept9	LOC105243612
Tmc6	LOC105243623
Tmc6	Tmc8
C1qtnf1	Gm35598
Cbx2	Gm35829
Tbc1d16	Ccdc40
Endov	LOC102639982
Nptx1	Gm11762
Chmp6	LOC105243645
Bahcc1	LOC102640779
Bahcc1	LOC105246895
Actg1	0610009L18Rik
Slc25a10	Gm11788
Alyref	Anapc11
Stra13	Lrrc45
Rfng	Gps1
Ccdc57	LOC105243655
Csnk1d	Gm30062
Ogfod3	Hexdc
Asxl2	1110002L01Rik
Itsn2	LOC105245369
1110057K04Rik	Gm33037
Sdc1	Gm4755
Vsnl1	4930511A02Rik
LOC102632102	LOC105245386
Itgb1bp1	Cpsf3
Ywhaq	Gm4419
Cys1	Gm36129
Mboat2	LOC102641621
Rnf144a	4930549C15Rik
Myt1l	LOC102634058
Pxdn	Gm31939
Acp1	Sh3yl1
Lamb1	Gm32899
Cbll1	LOC105244825
Sypl	4933406C10Rik
Atxn7l1	F730043M19Rik
Efcab10	Atxn7l1os2
Snx13	Gm33922
Meox2	LOC105244862
Arl4a	Arl4aos
Ifrd1	Gm7008
Foxg1	Gm34304
Arhgap5	Gm35188
Sptssa	LOC105245428
2700097O09Rik	Srp54a
Nfkbia	Gm36634
Nkx2-1	Gm26973
Nkx2-9	Gm26973
Lrfn5	Gm20063
Klhl28	LOC105244909
Wdr20rt	LOC105244910
Pygl	F730035M05Rik
Trim9	Gm32151
Arid4a	3110056K07Rik
Daam1	LOC105245431
Lrrc9	Gm33487
Trmt5	Slc38a6
Tmem30b	2210039B01Rik
Wdr89	Gm34717
Zbtb25	Zbtb1
Hspa2	4930426I24Rik
Zfyve26	Rad51b
Exd2	Gm30076
Galnt16	Gm26777
Srsf5	Gm20337

Numb	Gm31386
Elmsan1	Gm31513
Entpd5	Ccdc176
Aldh6a1	Lin52
Arel1	Fcf1
Mfsd7c	LOC105244960
Gpatch2l	LOC105244963
Angel1	Gm32755
Cipc	Gm32934
Gstz1	Gm6566
Tmed8	Samd15
LOC100503047	Gm5039
Nrxn3	Gm34382
Dio2	Gm26512
Foxn3	Gm26839
Ccdc88c	Gm36756
Fam181a	Gm29508
Ifi27	LOC105245052
Ppp4r4	Gm30995
Serpina3n	LOC105245053
Gsc	Gm10000
Syne3	Gm31742
Glrx5	Snhg10
Ak7	LOC105245063
Dio3	Dio3os
Ppp2r5c	LOC102638940
Dync1h1	Gm35558
Mok	4921507G05Rik
Mark3	2810029C07Rik
Inf2	Gm30048
Siva1	Gm30101
Akt1	Gm30150
Gpr132	Gm30461
Crip2	LOC102641016
Ncapg2	D430020J02Rik
Sp4	LOC105243776
Gm16505	Gm35043
Ggps1	Arid4b
Hecw1	Gm30893
Gli3	LOC105245355
Elmo1	Gm32036
Pgbd1	LOC105245359
Zkscan4	LOC105245360
Hist1h4h	Gm11335
Slc17a2	LOC105245367
Cmah	Gm11345
Dusp22	Gm35158
Serpinb6a	1110046J04Rik
Tubb2b	Gm36500
Pxdc1	Gm15908
Prpf4b	Gm36839
Eci3	Gm16984
Ppp1r3g	Gm30127
Lyrm4	Fars2
Cage1	Riok1
Txndc5	Gm31834
Slc35b3	5033403F01Rik
Gcnt2	Gm31683
Tmem170b	Gm32184
Tbc1d7	LOC105245481
Atxn1	5033430I15Rik
Ptpdc1	Gm36550
Barx1	Gm36606
Phf2	LOC102642832
Fam120a	Fam120aos
Cenpp	Nol8

Nutm2	LOC105245498
Spin1	9430083A17Rik
Ror2	Gm33574
Eif4e1b	Gm16249
Unc5a	Gm31313
Fam193b	Gm31364
Catsper3	4930451E10Rik
Hnrnpk	Rmi1
Naa35	A230056J06Rik
Spata31d1b	LOC105245526
Spata31d1d	4930528D03Rik
Isca1	Etohd2
Zcchc6	Gm34961
Fbp2	LOC105245535
Ptch1	LOC102632415
Cdc14b	1810034E14Rik
Cdk20	1700015C15Rik
Gm31714	LOC105245540
Zfp953	Gm17039
Zfp459	Gm33787
Zfp748	9430065F17Rik
Zfp738	Gm34146
Zfp65	Zfp85os
Mtrr	Fastkd3
Adcy2	Gm35161
Adamts16	Gm36529
Irx2	Gm20554
Ndufs6	Mrpl36
Nkd2	Gm30303
Trip13	Brd9
Zdhhc11	LOC105245558
Ell2	LOC105245562
Arsk	Ttc37
Nr2f1	A830082K12Rik
Mef2c	Gm33317
Tmem161b	A230107N01Rik
Hapln1	Gm34699
Ap3b1	Gm9776
Pde8b	Gm32586
Iqgap2	Gm33074
Sv2c	LOC105245595
Fcho2	Gm34995
LOC105245604	Gm35279
Map1b	Gm26559
Mast4	Gm30050
Nln	Sgtb
Trappc13	Trim23
Ppwd1	Cenpk
Cwc27	Srek1ip1
Kif2a	3830408C21Rik
Ndufaf2	Ercc8
Pde4d	Gm32703
Gapt	LOC105245629
Map3k1	Gm15327
Map3k1	LOC105245632
Ankrd55	LOC105245637
Mocs2	Gm34790
Itga1	LOC105245647
Gm7120	3110070M22Rik
Hmgcs1	LOC105245649
Nim1k	LOC105245650
Gm21818	LOC105243610
4930521O11Rik	4933406F09Rik
Oit1	LOC102632156
Oit1	LOC105245678
4930452B06Rik	4930455B14Rik

Synpr	Gm5087
Thoc7	Atxn7
Lrrc3b	B230110C06Rik
Ecd	Fam149b
Camk2g	Gm30108
Comtd1	A430057M04Rik
Zfp503	LOC102632594
Arhgef3	LOC105245708
Fam208a	LOC105245709
Il17rb	Chdh
Chdh	Gm35449
Sfmbt1	1700087M22Rik
Nek4	LOC105245713
Gnl3	Pbrm1
Nt5dc2	Smim4
Phf7	Bap1
Dph3	Oxnad1
Msmb	LOC105245719
3425401B19Rik	Gm28651
Vstm4	LOC105245723
Gdf10	Gm30031
Ccser2	Gm31131
Nrg3	LOC432842
Gm7233	Gm3141
Txndc16	Gpr137c
Gnpnat1	LOC105245744
Bmp4	LOC102638002
Samd4	Gm34934
Otx2	Otx2os1
Exoc5	Ap5m1
Tep1	Gm26782
Olfr1511	LOC105245752
LOC105245755	LOC105245757
Slc7a8	Rnf212b
Myh6	Mhrt
Psme2	Rnf31
Tgm1	Gm31979
Adcy4	Gm32092
Ripk3	Gm32092
Nfatc4	LOC105245760
Cbln3	Khnyn
Cma2	Mcpt-ps1
Cenpj	Parp4
Zmym5	2410022M11Rik
Gjb2	Gm32912
N6amt2	1700039M10Rik
Ska3	Mrpl57
Micu2	LOC105245763
Atp8a2	4930563I02Rik
Nupl1	LOC102642877
Amer2	Gm34935
C1qtnf9	Gm34997
Mipep	Mipepos
Tnfrsf19	LOC105245771
Arl11	1700109G14Rik
Rnaseh2b	LOC105245777
Gucy1b2	LOC105245776
Xkr6	LOC105245780
Pinx1	LOC105245781
Pinx1	LOC102640241
Prss52	4930471C04Rik
Stmn4	LOC105245791
Ebf2	LOC105245795
Cdca2	Kctd9
Nefl	Gm31107
Nkx2-6	1700092C10Rik

Slc25a37	Gm31748
Entpd4	Gm16677
Gm21464	Gm32162
Chmp7	4930480K23Rik
Sorbs3	Gm26908
Dmtn	LOC102642339
Gfra2	Gm34588
Htr2a	LOC105245820
Lrrc63	Lcp1
Zc3h13	Gm35679
Tpt1	Gm4285
Gtf2f2	Gm36429
Dgkh	Gm30716
Lect1	LOC105245828
Pcdh8	LOC102633266
Pcdh17	Gm32498
Dach1	Gm33583
Mzt1	Bora
Dis3	Pibf1
Klf12	LOC105245842
Prr30	Gm34361
Irg1	LOC105245846
Pou4f1	Gm35623
Uggt2	Gm16835
Tm9sf2	A330035P11Rik
Zic2	2610035F20Rik
Pcca	Gm33769
Ggact	4930594M22Rik
AW549877	A630020A06
Ttc33	LOC105245873
Osmr	LOC105245878
Slc1a3	Gm31340
Agxt2	LOC105245887
Rai14	4930556M19Rik
Amacr	Gm34315
6030458C11Rik	Drosha
Cdh6	LOC105245894
Cdh10	C030047K22Rik
Basp1	Gm5468
Ank	Gm36899
Ropn1l	Gm32271
March6	LOC105245907
Sema5a	Snhg18
Cpq	Gm32764
Gm32970	Kcns2
Fbxo43	Spag1
Ywhaz	LOC105245918
Grhl2	LOC105245924
Fzd6	LOC105245938
Emc2	Gm10373
Sybu	Gm33908
Sybu	2310069G16Rik
Csmd3	Gm34336
Enpp2	Gm26684
Mrpl13	Mtbp
Ndufb9	Gm36559
Tatdn1	Ndufb9
E430025E21Rik	Nsmce2
Gsdmc2	Gsdmcl1
Gsdmc3	Gsdmcl2
Gsdmc4	Gsdmcl-ps
Asap1	Gm30563
Efr3a	LOC105245968
Kcnq3	Gm27242
Lrrc6	Gm31006
Ndrg1	LOC105245970

Arc	Gm32994
Lypd2	Gm33124
Gml	Hemt1
Zc3h3	Gm34851
Cyhr1	Kifc2
Foxh1	Ppp1r16a
Arhgap39	LOC105245982
Apol10b	Gm36245
Cacng2	LOC102640344
Sh3bp1	LOC102632079
Lgals1	Gm30368
H1f0	Gm17753
Sox10	Gm10863
Pick1	Gm30527
Slc16a8	Gm17209
Baiap2l2	Gm30753
Sun2	Gm16576
Dnal4	Gm30977
Apobec3	D730005E14Rik
Mgat3	Gm31196
Mkl1	4930483J18Rik
Tef	Gm17597
Csdc2	Gm32167
Desi1	Xrcc6
Sept3	Gm32310
Nfam1	Gm32620
Pnpla5	Gm33432
Pnpla3	Gm33530
1810041L15Rik	Gm33830
Prr5	LOC105246006
Arhgap8	Gm20556
5031439G07Rik	LOC105243769
Smc1b	Ribc2
Wnt7b	AU022754
Celsr1	Gm34764
Gramd4	LOC105246009
Fam19a5	Gm35471
Brd1	LOC105246012
Mov10l1	Gm8702
Trabd	1810021B22Rik
Mapk11	LOC105246014
Lmf2	Ncaph2
Acr	Gm36221
Ano6	A130051J06Rik
Ano6	LOC102634389
Rapgef3	LOC102636367
Hdac7	Gm33690
Ccnt1	4930415O20Rik
Tuba1a	LOC105246034
Troap	Gm34284
Mcrs1	Gm34447
Nckap5l	Gm34594
Asic1	Gm34880
1700030F18Rik	Gm21917
Larp4	2310068J16Rik
Slc11a2	Gm5475
Dazap2	C330013E15Rik
Grasp	A330009N23Rik
Krt7	LOC102639385
Krt8	Gm36026
Aaas	Gm36246
Pcbp2	Gm36331
Hoxc12	Gm36820
Hoxc8	Gm29852
Hoxc6	Gm29905
Hoxc4	Gm30528

Cbx5	Hnrnpa1
Ppp1r1a	LOC105246048
Glis2	LOC105246062
Cdip1	LOC105246063
Anks3	4930451G09Rik
Ubn1	Gm36765
Carhsp1	Gm5767
Snn	Gm9861
Zc3h7a	LOC105246071
Rsl1d1	2610020C07Rik
Shisa9	Gm32278
Rrn3	LOC101055727
Mzt2	LOC105246077
Vpreb1	Top3b
Aifm3	LOC105246079
Lrrc74b	P2rx6
Dgcr2	Tssk1
LOC102638083	4933432I09Rik
Tango2	Arvcf
Comt	Gm15764
Tbx1	Gm35369
Cdc45	Ufd1l
Mrpl40	Hira
Clcn2	Polr2h
Thpo	Chrd
Map3k13	LOC105246086
St6gal1	BC106179
Bcl6	Gm31527
Lpp	1110054M08Rik
Fgf12	LOC105246093
Ncbp2	0610012G03Rik
Zfp148	1700007L15Rik
Ccdc14	Gm35150
Dirc2	Hspbap1
Kpna1	Gm15564
Kpna1	LOC105246107
Gtf2e1	Rabl3
Gpr156	Gm36225
Gsk3b	BC031361
Pla1a	Gm36333
4930435E12Rik	LOC102641625
Zbtb20	4932412D23Rik
BC027231	LOC105246114
Dzip3	C330027C09Rik
Cd47	Gm4633
Senp7	LOC105246121
Tmem30c	LOC105246122
Dcbld2	4930461C15Rik
St3gal6	Gm33475
Mina	1700022E09Rik
Epha6	LOC102636664
Robo1	Gm29908
Usp25	1700041M19Rik
Atp5j	Gabpa
Cct8	B130034C11Rik
Gm35427	LOC105246153
Mis18a	Gm17518
Synj1	4930404I05Rik
Gart	Son
Donson	Gm10785
Kcne1	Gm30163
Cldn14	Gm31012
Cldn14	LOC102633190
Sim2	Gm31175
Kcnj6	Gm31407
Ets2	Gm31989

Tfb1m	1700102H20Rik
Tagap1	Gm36413
Fgfr1op	Gm33748
Sft2d1	4930506C21Rik
Qk	B930003M22Rik
Map3k4	4732491K20Rik
Tcp10b	Gm36226
Unc93a	Gm36277
Tcp10c	Gm36537
Tcte3	Ermard
Riok2	LOC105246224
Riok2	Gm36768
Prss22	LOC105246235
Pdpk1	Gm33409
Atp6v0c	LOC105246236
Rnps1	D330041H03Rik
Pgp	4833447I15Rik
Traf7	Rab26os
Rab26	Gm33508
Tsc2	Nthl1
Rps2	Snhg9
Igfals	LOC105246237
Nubp2	Spsb3
Cramp1l	Ift140
Ube2i	Gm17801
Cacna1h	Gm33565
Sox8	2810468N07Rik
Capn15	1700022N22Rik
Ergic1	1700049J03Rik
Cuta	Syngap1
Bak1	Ggnbp1
Rps10	Gm15420
Anks1	Gm35290
Armc12	LOC105246172
Srsf3	LOC105246247
Cpne5	LOC105246249
Cmtr1	Gm20161
Dnah8	1700097N02Rik
Zfp871	Gm17115
Zfp472	Cyp4f41-ps
Morc2b	LOC105246265
Rps28	Ndufa7
Daxx	BC051226
Rps18	Vps52
H2-K1	LOC105246267
Brd2	H2-DMa
H2-Eb1	LOC105245179
Stk19	Dxo
D17H6S53E	LOC105245272
Pou5f1	Gm32362
Ddr1	LOC105246271
Tubb5	Gm32463
Atat1	LOC102635200
Mrps18b	Ppp1r10
Abcf1	Gm32655
Gm318	Gm33537
Trim15	Trim10
Gabbr1	Gm33802
Olfr90	LOC105246273
Rcan2	Gm34773
Enpp4	Gm34917
Hsp90ab1	Gm35399
1600014C23Rik	F630040K05Rik
Lrrc73	LOC105246282
Klhdc3	Mea1
Trerf1	LOC105246285

Foxp4	LOC102642815
Tspo2	Unc5cl
Ccdc94	Gm16712
Ptprs	Gm32822
Safb2	Safb
Acsbg2	1700061G19Rik
Clpp	Gm33509
Gpr108	Trip10
Nudt12	Nudt12os
Txndc2	Gm35477
Zbtb14	Gm36542
A330050F15Rik	A930029G22Rik
Tgif1	Gm36856
Wdr43	Gm30091
Clip4	Gm30140
Lbh	Gm30375
Birc6	Gm31645
Crim1	Gm32282
Cebpzos	LOC105246319
Cebpz	Ndufaf7
Rmdn2	LOC105246322
Srsf7	Gemin6
Dhx57	Morn2
Cdkl4	Gm33373
Pkdcc	Gm34686
Zfp36l2	Gm36279
Abcg5	Abcg8
Ppm1b	1110020A21Rik
Prepl	Camkmt
Six3	Six3os1
Six2	CJ186046Rik
Socs5	Gm30510
Ttc7	4833418N02Rik
Fbxo11	Gm4832
Ston1	Gm31704
Nrxn1	Gm32337
Mettl4	2700099C18Rik
Gm20939	LOC102635273
Crem	Gm6225
Map3k8	4833419F23Rik
Zeb1	Gm10125
Abhd3	4930563E18Rik
Gata6	1010001N08Rik
Tmem241	LOC105246369
Lama3	LOC105246370
Zfp521	Gm29911
Mapre2	Gm15972
Zscan30	Zfp35
Tpgs2	AW554918
Celf4	Gm32896
Polr2d	LOC105246388
Map3k2	Gm34806
Camk4	2310026I22Rik
Nrep	Gm10549
Cdc25c	2010110K18Rik
Nrg2	LOC105246402
Hbegf	LOC105246406
Hars	Hars2
Hdac3	Rell2
Pcdh1	2010320O07Rik
0610009O20Rik	1700086O06Rik
Rbm27	Gm4013
Kcnn2	Gm32139
Dtwd2	Gm16283
Tnfaip8	LOC102635912
Prdm6	LOC105246436

Zfp608	LOC105246443
Zfp608	Gm4221
Aldh7a1	Phax
March3	Gm34632
1700011I03Rik	1700066O22Rik
Fbn2	LOC105246454
Rbm22	Gm35403
Grpel2	1500015A07Rik
Adrb2	Gm9949
Spink13	LOC105246465
Spink13	Gm36598
Wdr7	LOC105246469
Nedd4l	A330084C13Rik
Nedd4l	LOC105246471
Zfp532	LOC105246475
Seh1l	Gm26910
Cep192	4930549G23Rik
Fam210a	Rnmt
Rab27b	LOC105246485
4930503L19Rik	Stard6
Mbd2	Gm31671
Mapk4	Gm9925
Myo5b	1700120E14Rik
Acaa2	LOC105246489
Smad7	Gm20544
Zbtb7c	Gm33703
Ier3ip1	Gm32058
Pias2	LOC102634716
St8sia5	Gm7276
8030462N17Rik	4930465K10Rik
Galr1	Gm30642
Zfp516	4930592I03Rik
Ighmbp2	Mrpl21
Lrp5	Gm36672
1810055G02Rik	Gm36608
Rad9a	Gm30082
Clcf1	LOC102631992
Rhod	A930001C03Rik
Sptbn2	LOC105246839
Rbm4	Gm30496
B4gat1	Gm30581
Klc2	Gm30840
Mus81	Cfl1
Snx32	Gm31166
Rnaseh2c	LOC105246844
Kcnk7	LOC105243849
Dpf2	LOC105246846
Snx15	Gm31978
Ehd1	LOC105246847
Cdc42bpg	Gm32042
Sf1	LOC102634533
Nrxn2	LOC105246848
Fkbp2	LOC105246528
Dnajc4	Gm35838
Nudt22	Trpt1
Mark2	LOC102639888
Chrm1	9830166K06Rik
Slc3a2	Snhg1
Nxf1	LOC102640359
Tmem179b	Gm2518
Ttc9c	Hnrnpul2
1810009A15Rik	5730408K05Rik
Rom1	Eml3
Mta2	LOC102640526
Vps37c	LOC105246535
Cd5	A430093F15Rik

Ms4a13	4930526L06Rik
Patl1	Gm31092
Olfr1447	Gm5512
Cep78	C130060C02Rik
Trpm6	LOC105246540
Tmc1	LOC105246543
Trpm3	LOC102641617
Smc5	Gm33950
Dmrt2	2610016A17Rik
Smarca2	Gm20616
D19Bwg1357e	C030016D13Rik
Glis3	D930032P07Rik
Ric1	A930007I19Rik
Sgms1	2700046G09Rik
Rnls	Gm29998
Hectd2	LOC105246565
Cyp26a1	Gm32342
Pdlim1	LOC105246573
Aldh18a1	Gm27042
Tctn3	Entpd1
Hps1	LOC102637720
Nkx2-3	Gm20467
Slc25a28	BC037704
Kazald1	Gm35782
Nfkb2	4833438C02Rik
Cuedc2	Gm36493
Arl3	Sfxn2
Usmg5	Pdcd11
Gsto1	Gm30021
Add3	Gm9618
Mxi1	Gm30541
Pdcd4	Gm30990
Gpam	Gm31356
Gpam	Gm31418
Luzp4	LOC105246600
Tcf7l2	Gm31734
Dclre1a	Nhlrc2
Fam160b1	B230217O12Rik
Gfra1	LOC105246609
1700019N19Rik	LOC105246610
Vax1	4930442E04Rik
Emx2	Emx2os
Cacul1	LOC105246616
Meig1	Dclre1c
Dclre1c	Gm35975
Hspa14	Cdnf
Sec61a2	Gm13267
Proser2	Gm10857
Gata3	4930412O13Rik
Fbxo18	Ankrd16
Rsu1	LOC105244084
Stam	Stamos
Cacnb2	Gm32741
Mllt10	LOC105244088
Commd3	Gm33355
Pip4k2a	4930426L09Rik
Otud1	Gm3230
Arhgap21	Gm13375
Pdss1	LOC105244094
Mrpl41	Pnpla7
Ndor1	Tmem203
Man1b1	AA543186
Edf1	Gm35476
Rabl6	LOC105244097
Ccdc183	LOC102639143
Fcna	Gm35630

4932418E24Rik	Gm35807
Sec16a	0610009E02Rik
Notch1	Gm35978
Stkld1	Gm36591
Dbh	Dbhos
Rxra	Gm30016
Gtf3c4	Ddx31
Trub2	Coq4
Ccbl1	Gm31201
Lrrc8a	1700084E18Rik
Ier5l	Gm31291
Usp20	LOC105244113
Gpr107	D330023K18Rik
Hmcn2	Gm32866
Fam78a	Gm33126
Pomt1	LOC105244114
Swi5	Golga2
Dnm1	LOC105244115
Pip5kl1	9430097D07Rik
Ttc16	Ptrh1
Lrsam1	Rpl12
Garnl3	Gm33536
Lmx1b	C130021I20Rik
Rabepk	Gm34604
Hc	LOC105244126
Gsn	LOC105244127
4930568D16Rik	Gm35093
Lhx2	Gm27197
Zeb2	Zeb2os
Orc4	Mbd5
Mmadhc	Gm13483
Gpd2	A930012O16Rik
Pkp4	LOC102635089
Baz2b	Gm13572
Baz2b	March7
Kcnh7	Gm34110
Scn1a	Gm13629
Stk39	LOC102641539
4933409G03Rik	Gm36002
Mettl8	Dcaf17
Sp3	Sp3os
Cir1	Scrn3
Gpr155	Gm13707
Evx2	Gm31790
Hoxd1	Haglr
Hnrnpa3	LOC105244210
Nfe2l2	E030042O20Rik
Agps	Gm34861
Fkbp7	LOC105244212
Fkbp7	Plekha3
Prdx6b	Gm36153
Slc43a1	LOC105244220
LOC102632821	Psmc3
Slc39a13	LOC105244227
Ddb2	A330069E16Rik
Zfp408	Arhgap1
Atg13	Harbi1
Mdk	LOC105244229
Gyltl1b	LOC105244233
Gyltl1b	Pex16
Syt13	Gm13791
Cd82	Gm10804
Hsd17b12	LOC105244242
Ttc17	2810002D19Rik
Caprin1	LOC105244252
Fbxo3	4931422A03Rik

D430041D05Rik	Gm35606
Qser1	LOC105244256
Eif3m	LOC102639549
Wt1	Wt1os
Mpped2	Gm29885
Lin7c	LOC105244266
Actc1	C130080G10Rik
BC052040	LOC102633794
Meis2	G630016G05Rik
Tmco5	Gm13990
Thbs1	Gm13986
Gpr176	1700054M17Rik
Ivd	LOC105244272
Bahd1	Gm32957
Dnajc17	Zfyve19
Exd1	Chp1
Lrrc57	Haus2
Ttbk2	AV039307
Zscan29	Tubgcp4
Duoxa1	Duox1
Shf	LOC105244278
Slc30a4	4930417H01Rik
Bloc1s6	Bloc1s6os
Gabpb1	LOC105244282
Ncaph	Gm35347
Kcnip3	Gm35478
Bcl2l11	LOC105244287
Mertk	LOC105244292
Ckap2l	Gm14023
Ebf4	Gm30169
Pced1a	Vps16
Mrps26	4930473A02Rik
Atrn	A730017L22Rik
1700037H04Rik	Gm14232
Rnf24	Gm30590
Smox	Gm30679
Rassf2	LOC105244302
Rassf2	Gm31179
Pcna	LOC102633666
Trmt6	Mcm8
Mkks	Slx4ip
Esf1	Ndufaf5
Kif16b	Kif16bos
Bfsp1	Gm33648
Sec23b	Gm34292
Nkx2-4	Gm34889
Nkx2-2	6430503K07Rik
Pax1	AI646519
Pygb	Gm30141
Fkbp1a	Gm14167
Sdcbp2	LOC105244326
Scrt2	AA387200
Srxn1	Gm31475
Trib3	Gm14164
Zcchc3	6820408C15Rik
Defb21	LOC105244328
Kif3b	LOC105244330
Asxl1	2500004C02Rik
Nol4l	4930404H24Rik
6430550D23Rik	Ergic3
Spag4	LOC105244341
Dlgap4	Gm14169
Dlgap4	4930405A21Rik
Tgif2	Gm14230
1110008F13Rik	5430405H02Rik
Manbal	LOC105244346

Tti1	Rprd1b
Slc32a1	Gm14204
Plcg1	Gm35032
Zhx3	Gm35129
Emilin3	Gm35181
Chd6	LOC105244350
Ptprt	9430021M05Rik
Pkig	LOC102639518
Rims4	Gm35886
Tomm34	Stk4
Zfp335	Zfp335os
Cdh22	Gm36748
Zfp663	Gm29831
Zmynd8	Gm11465
Zmynd8	LOC105244357
Sulf2	LOC105244358
Cse1l	Gm31204
Kcnb1	1110018N20Rik
Ube2v1	Gm31852
Pard6b	LOC105244367
Sall4	LOC105244370
Zfp64	LOC105244371
Rab22a	Ppp4r1l-ps
Gnas	Gm30189
Slmo2	LOC102641287
Zfp931	Gm35319
Cdh26	Gm35532
Taf4a	4921531C22Rik
Adrm1	Gm29886
Rbbp8nl	Gm29943
Mrgbp	Gm30144
Tcfl5	Gm30345
Slc17a9	Gm30455
Ptk6	LOC105244424
Arfrp1	Zgpat
Uckl1	Uckl1os
Zfhx4	2700069I18Rik
Il7	LOC105246750
Ralyl	Gm2464
Nlgn1	A830092H15Rik
Ect2	1700125G22Rik
Rpl22l1	Gm32950
Mecom	LOC105247014
Gpr160	LOC105247016
Slc7a14	Gm15496
Kcnmb2	Gm34031
Gnb4	Gm34100
Dcun1d1	Gm15952
Mfsd8	Gm2011
Jade1	LOC105244433
Sclt1	D3Ertd751e
Pcdh10	2610316D01Rik
Postn	Gm31095
Sertm1	Gm31358
Ccna1	4931419H13Rik
Siah2	4930593A02Rik
P2ry1	LOC105244455
Rarres1	LOC105244464
Zbbx	LOC105244469
Fnip2	4930589L23Rik
Tmem144	LOC105244471
Rbm46	Rbm46os
Lrat	Rbm46os
D930015E06Rik	Gm30352
Gatb	Gm3740
Rrnad1	Isg20l2

Mef2d	LOC105244488
Mex3a	LOC102642612
Lamtor2	Ubqln4
Arhgef2	LOC105244493
Arhgef2	Gm20652
Syt11	5830417I10Rik
Gon4l	1500004A13Rik
Fam189b	Gm16069
Mtx1	Thbs3
Trim46	Krtcap2
Zbtb7b	Gm15417
Hax1	Gm19710
Rps27	Rab13
Ivl	Gm35639
Smcp	Gm35639
Them4	Gm36010
Rorc	LOC329711
Tdrkh	1700040D17Rik
Ctss	LOC105244499
Golph3l	LOC105244500
Rprd2	Gm29948
Mrps21	C920021L13Rik
Otud7b	Gm30044
Sf3b4	LOC105244501
BC107364	LOC102632901
Lix1l	Gm31059
Rbm8a	6330549D23Rik
Polr3c	Rnf115
Pdzk1	4930442L01Rik
Zfp697	Gm31765
Wdr3	Gdap2
Fam46c	Gm12474
Hipk1	LOC102637286
Dclre1b	Ap4b1
Phtf1	Phtf1os
Capza1	St7l
Kcnd3	LOC102641256
Dennd2d	2010016I18Rik
Strip1	Gm10961
Ahcyl1	LOC105244527
Ampd2	Gm12500
Sypl2	LOC105244529
Psrc1	Gm12522
Slc25a24	LOC105244530
Dph5	A930005H10Rik
Cdc14a	LOC105244532
Trmt13	Sass6
Ptbp2	LOC102641335
Slc44a3	A530020G20Rik
Bcar3	Gm34349
1810037I17Rik	4933405D12Rik
Larp7	Zgrf1
Pitx2	LOC105244556
Elovl6	Gm35986
Lef1	Gm36603
Sgms2	Gm36753
Aimp1	Tbck
Ube2d3	4930539J05Rik
Manba	Gm30992
Pkn2	9530052C20Rik
Odf2l	LOC105244570
Syde2	Gm35352
Dnase2b	Uox
4930503B20Rik	Gm36521
Dnajb4	Fubp1
Nexn	Gm31999

Lhx8	AI606473
Negr1	4930570G19Rik
Zranb2	LOC102636317
Cth	1810013D15Rik
Srsf11	Lrrc40
Depdc1a	LOC105244597
Lyn	LOC105247090
Rps20	Gm32823
Plag1	Chchd7
Clvs1	Gm34288
Ccne2	A630034I12Rik
Dpy19l4	LOC105247095
Esrp1	Gm35631
Rad54b	Gm35693
Pdp1	1700123M08Rik
Tmem67	Rbm12b2
Ripk2	A530072M11Rik
Cnbd1	LOC105247099
Ttpa	4930480G23Rik
Ggh	LOC105247101
Fbxl4	LOC105247103
Klhl32	Gm31379
Gpr63	C230012O17Rik
Ube2j1	4933421O10Rik
Lingo2	Gm12371
1700009N14Rik	Gm17094
Dnaja1	Gm6297
1110017D15Rik	Fam219aos
Fam219a	Dnaic1
Enho	Gm12405
Galt	LOC105247116
Fam214b	LOC105247120
Olfr156	Gm30318
Glipr2	Gm30377
Zbtb5	1700055D18Rik
Trim14	LOC105247126
Tbc1d2	LOC105247124
Baat	Gm33060
Tmem246	Gm33106
Rnf20	Gm33159
Ppp3r2	LOC105247131
Smc2	Smc2os
Fsd1l	Gm33729
Klf4	Gm12506
Ptpn3	Gm12536
D630039A03Rik	LOC105247139
Txn1	Gm35344
AI481877	Gm35527
Mup1	Gm13773
Mup12	Mup-ps8
Mup15	Mup-ps12
Mup16	Mup-ps12
LOC100048884	LOC102631971
Mup19	Mup-ps16
Mup3	LOC105243851
Zfp37	Gm30451
Cdc26	Prpf4
Rnf183	Gm30589
Zfp618	Gm30764
Tle1	C630043F03Rik
Rasef	Gm11240
Ptprd	LOC105247151
Ttc39b	LOC105247156
Ttc39b	LOC105247155
Elavl2	LOC105244619
Izumo3	LOC105244620

Mysm1	LOC105244623
Jun	Junos
Fggy	LOC105244625
Hook1	9530080O11Rik
Gm12695	Gm34053
Nfia	LOC105242444
Ror1	Gm35701
Tctex1d1	Gm12709
4921539E11Rik	4930456L15Rik
Oma1	Gm36523
Dab1	Gm36604
Prkaa2	Gm36893
Ppap2b	Gm29838
Usp24	Gm30429
Pcsk9	Gm30487
Mrpl37	Cyb5rl
Lrp8	LOC105244644
Zyg11a	LOC105244647
Eps15	LOC105244653
Ttc39a	Gm12750
Dmrta2	Dmrta2os
Foxd2	Foxd2os
Tal1	Gm33786
Mmachc	Ccdc163
Toe1	Mutyh
Hpdl	Gm12996
Urod	Hectd3
Tctex1d4	Btbd19
Rnf220	LOC102637578
Ccdc24	LOC105244667
Atp6v0b	Gm34812
2610528J11Rik	LOC105244670
Tmem125	LOC105244671
Guca2b	Gm35761
Mycbp	D130007C19Rik
Pou3f1	Gm2164
Maneal	Epha10
Csf3r	Gm32908
Gjb3	LOC105244689
CK137956	LOC102641725
Hmgb4	Hmgb4os
Rnf19b	1700086P04Rik
S100pbp	Yars
C77080	Gm12976
Rbbp4	Zbtb8os
Serinc2	Gm34957
Fabp3	LOC102638454
Sdc3	LOC105247170
Laptm5	LOC102642196
Tmem200b	Gm12992
Epb4.1	Gm36157
LOC105247180	Kdf1
Gm34296	LOC105247182
Grrp1	LOC105247183
Id3	LOC105247187
Ephb2	Gm31536
Hspg2	LOC105247191
Rap1gap	Rap1gapos
Pla2g5	Gm32930
Rnf186	LOC105247194
Pqlc2	Akr7a5
Padi3	Gm13032
Arhgef19	LOC102637755
Zbtb17	LOC105247198
Clcn6	Mthfr
Fbxo44	Fbxo2

Slc2a5	LOC102641052
Errfi1	1700045H11Rik
Camta1	LOC102631977
Tas1r1	Nol9
Rpl22	LOC102632664
Smim1	Gm31672
Tprgl	LOC102634100
Prdm16	Gm13111
Mmel1	LOC102634887
Cdk11b	Gm16023
Tmem240	LOC102636313
Atad3a	Atad3aos
Pusl1	Acap3
Tnfrsf18	Gm10560
Isg15	AW011738
Klhl17	Noc2l
Samd11	Gm33896
Cdk6	Gm36470
Ankib1	Krit1
Cdk14	Gm30267
1700015F17Rik	Gm30325
Cfap69	LOC105244703
Steap4	Gm30835
Abcb1b	Gm5106
Dmtf1	Gm31539
Gm6650	LOC105244708
Magi2	4921504A21Rik
Phtf2	LOC105247041
Ptpn12	LOC105247043
Ccdc146	Fam185a
Orc5	6030443J06Rik
Kmt2e	5031425E22Rik
Cdk5	Slc4a2
Wdr86	LOC105247056
Prkag2	2900005J15Rik
Kmt2c	4831440E17Rik
Gm1979	Gm5067
Rbm33	Gm35602
Shh	9530036O11Rik
Cib4	LOC105244714
Agbl5	Gm35795
Nrbp1	LOC105244716
Plb1	Gm36783
Fam53a	Gm9903
Tmem129	Tacc3
Gm1673	Gm30802
Nop14	Grk4
Hgfac	LOC105244722
Hmx1	E130018O15Rik
Sh3tc1	Gm31888
Grpel1	2210406O10Rik
Jakmip1	Gm33166
Msx1	Gm33993
Zfp518b	LOC105244734
C1qtnf7	LOC105244739
Cc2d2a	Gm35762
Tapt1	Gm35959
Ncapg	1600023N17Rik
Dhx15	9230114K14Rik
Slc34a2	Gm17182
Smim20	LOC105244747
Pcdh7	4932441J04Rik
Dcun1d4	LOC100862249
Spata18	Gm34902
Lnx1	Gm15984
Clock	Gm7467

Pdcl2	4930432L08Rik
Cabs1	1700066N21Rik
Rufy3	LOC105246910
Ankrd17	Gm9958
Nup54	LOC105246918
Naa11	4930467D21Rik
Prkg2	LOC105246929
Hnrnpdl	Enoph1
Wdfy3	Gm29707
1700016H13Rik	LOC105246944
Sparcl1	LOC105246946
Lrrc8b	4930542N06Rik
Barhl2	Gm33151
Brdt	Gm33692
Gfi1	A430072P03Rik
Atp5k	Gm34319
Gak	Tmem175
Fgfrl1	LOC105246959
LOC105246961	Gm35089
Golga3	Gm15787
Ankle2	LOC105246962
Noc4l	Ddx51
Hscb	Chek2
Tfip11	LOC105246967
2900026A02Rik	LOC105246973
Tmem119	LOC105246762
Kctd10	Ube3b
Tchp	Gm34931
Ankrd13a	4930515G01Rik
Oasl1	LOC105246766
Mlec	Gm35465
Cabp1	Gm35515
Dynll1	LOC105246767
Msi1	LOC102639568
Sirt4	LOC105246768
Rab35	1110006O24Rik
Cit	LOC105246769
Cit	Gm36185
Hspb8	LOC105246770
Wsb2	LOC105246773
Rfc5	LOC102632031
Rnft2	2410131K14Rik
Tbx5	Gm5563
Lhx5	Gm31976
Lhx5	Gm27199
Tpcn1	Iqcd
Rita1	Ddx54
Oas1c	Oas1b
Trafd1	LOC105246782
Sh2b3	Gm32422
Rnf34	Gm33044
Kdm2b	A930024E05Rik
Orai1	Gm2479
Rsrc2	Kntc1
Vps37b	Gm34086
Pitpnm2	LOC105246789
Pitpnm2	LOC105246788
Mphosph9	2810006K23Rik
Fzd10	5930412G12Rik
Sfswap	LOC105244780
Crcp	LOC105244784
Sbds	Tyw1
Gatsl2	Gm15627
Wbscr27	LOC105244793
Hip1	LOC105244796
Por	LOC105244797

Sh2b2	Gm30408
Muc3	LOC105244802
Ache	Gm35872
Srrt	LOC105242537
Mospd3	Gm7285
Nxpe5	Gm454
BC037034	6330418K02Rik
Gpc2	Stag3
Dnaaf5	LOC105242543
Iqce	Brat1
Wipi2	LOC105242553
Fbxl18	LOC105242554
Spdyb	4930448H16Rik
Zfp12	LOC105242556
Zdhhc4	0610040B10Rik
Kdelr2	LOC105242557
Usp42	LOC105242559
Aimp2	Pms2
Ccz1	Gm33427
Baiap2l1	Dmr
Tmem130	LOC105242562
Zscan25	LOC105242563
Rnf6	LOC105242571
Usp12	LOC102638199
Katnal1	Gm36446
Uspl1	5730422E09Rik
Hsph1	Gm20005
Rxfp2	LOC105242583
Calcr	Gm33576
Glcci1	A430035B10Rik
Tes	Gm15473
Tspan12	Gm30270
Ube2h	LOC102633456
Cpa5	Gm31453
Mkln1	Mkln1os
Slc35b4	LOC105242626
Cnot4	Gm33046
Chrm2	9330158H04Rik
Creb3l2	Gm33318
Parp12	4930599N23Rik
Mkrn1	Gm10244
Trpv6	Gm34642
Kel	LOC105242637
Olfr13	1700024N05Rik
LOC105244833	LOC105244835
LOC105244832	LOC105244834
Gm36327	LOC105244830
Krba1	Gm16630
Zfp467	Sspo
Gimap1	Gimap1os
Mpp6	LOC105242649
Cycs	5430402O13Rik
Hnrnpa2b1	Cbx3
Hoxa1	LOC105242658
Hoxa9	LOC105242659
Hoxa13	Hottip
Cpvl	4921529L05Rik
Chn2	Gm15527
Fkbp14	Plekha8
Plekha8	LOC105242665
Atoh1	Gm35851
Ndnf	Gm36144
Cd8b1	LOC105242677
Ptcd3	Polr1a
Vamp5	Gm30872
Dnah6	LOC105244843

Htra2	Aup1
Mrpl53	Ccdc142os
Tet3	B230319C09Rik
Vax2	Vax2os
Ankrd53	1700124L16Rik
Nagk	Gm7443
Spr	Gm10444
Emx1	LOC105242700
Rab11fip5	LOC105242701
Figla	Gm33319
Pcbp1	1600020E01Rik
Aak1	2610306M01Rik
Aplf	E230015B07Rik
H1fx	Gm5577
Gata2	LOC105242443
Abtb1	Gm15612
Nup210	Gm30364
Wnt7a	Gm30437
Magi1	Gm31661
Kbtbd8	LOC102634268
Lmod3	Gm32191
Frmd4b	Gm32380
Frmd4b	Gm32247
Prok2	Gm26748
Cntn4	Gm15631
Itpr1	LOC105242735
Bhlhe40	4833447P13Rik
Edem1	Gm35417
Grm7	Gm20387
Rad18	Gm35824
Setd5	Gt(ROSA)26Sor
Hrh1	LOC105242741
Syn2	Gm17733
Tsen2	Gm36355
Fam21	9530062K07Rik
Gm8213	Fam21
Wnt5b	LOC105244866
Erc1	3110021A11Rik
Ccdc77	Kdm5a
Bcl2l13	LOC105242759
Pex5	LOC102634873
P3h3	Gm32404
Ptms	LOC105242768
Pianp	LOC105244867
Scnn1a	LOC105242770
Dyrk4	LOC102641700
Rad51ap1	D6Wsu163e
Prmt8	LOC105244871
Fkbp4	Gm10069
Gpr19	LOC105244877
Gpr19	2810454H06Rik
8430419L09Rik	Gm36533
Grin2b	Gm26653
Rerg	LOC105242804
Lmo3	A830011K09Rik
Lmo3	B230110G15Rik
Pde3a	Gm28523
Recql	Golt1b
Sox5	LOC102641078
Bcat1	Gm26666
Kras	Gm15706
Tuba3b	LOC102635150
Rassf8	LOC102641081
Asun	Fgfr1op2
Ccdc91	Gm6288
Ergic2	4732416N19Rik

Tmtc1	Rps4l
Ipo8	LOC105242826
Fam60a	3010003L21Rik
Cacng6	3300002P13Rik
Cnot3	Gm15927
Leng8	D030047H15Rik
Suv420h2	LOC105247237
Isoc2b	LOC105242842
Ccdc106	Gm15510
Zfp787	Gm36371
Zfp28	LOC105242846
Vmn1r74	Gm6929
Crx	Crxos
Gltscr2	Gm24576
Dact3	9330104G04Rik
Irf2bp1	Mypopos
Klc3	LOC105242863
Tomm40	LOC105242864
Pvrl2	Gm34744
Irgc1	Gm36159
Pinlyp	Xrcc1
Lypd3	Gm26550
Rps19	LOC105242878
Atp1a3	Gm36861
Dedd2	Zfp526
Bckdha	Exosc5
Cyp2a4	LOC105247243
Snrpa	BC024978
Sertad1	LOC105242886
Pld3	2310022A10Rik
Ttc9b	LOC105242888
Map3k10	C030039L03Rik
Zfp59	Gm30891
Zfp780b	LOC105242889
Fbl	LOC105242891
Rps16	Gm31383
Nccrp1	LOC105242893
Sipa1l3	4932431P20Rik
Zfp84	LOC105242900
Zfp74	C230062I16Rik
Zfp568	Gm31735
Tyrobp	Gm32029
Kmt2b	Gm32082
Tmem147	Tmem147os
Cd22	D7Ertd128e
Usf2	Gm4673
Lsr	Fam187b
Gpi1	Gm12758
Chst8	LOC105242908
C230052I12Rik	Cep89
Slc7a9	LOC105247248
Rgs9bp	Ankrd27
Zfp507	E130304I02Rik
Tshz3	Gm36722
Zfp536	LOC102642793
Gm6818	LOC105242406
Gm5591	Gm29087
Gm6833	Gm32110
Gm1988	Gm33067
Gm29258	Gm33382
Zfp939	Gm5590
Vstm2b	A230077H06Rik
Gm4884	LOC105242923
AI987944	Gm17102
Ctu1	Gm36546
Gm36864	1700028J19Rik

Acpt	Gm15517
1700008O03Rik	LOC105242929
Kcnc3	Gm15396
Myh14	2310016G11Rik
Tbc1d17	Akt1s1
Prmt1	Gm15545
Bcl2l12	Irf3
Trpm4	Hrc
Ppfia3	Mtag2
Sec1	Ntn5
Grin2d	Kdelr1
Emp3	Ccdc114
Ldhc	LOC105242939
Spty2d1	Gm32031
Ano5	Gm33190
Slc17a6	LOC105242942
Fancf	Gas2
Nipa2	A230056P14Rik
Herc2	Gm34121
Gabrg3	Gm9962
Gabrb3	LOC105247256
Tjp1	Gm33068
Tm2d3	Gm33234
Aldh1a3	Gm33515
Asb7	Lins
Lysmd4	1700112J16Rik
Lrrc28	Ttc23
Fam169b	Gm16157
Mctp2	LOC105242973
Slco3a1	LOC102642641
Sv2b	1500012K07Rik
Aen	LOC105242979
Abhd2	Gm31510
Polg	LOC105242982
2610034B18Rik	Gm31966
Zfp592	Gm32178
Slc28a1	LOC105247271
Mex3b	Gm36584
Stard5	Gm16638
Abhd17c	Gm26708
Picalm	2310010J17Rik
Ankrd42	Gm26944
Rab30	4632427E13Rik
Thrsp	Gm32945
Clns1a	LOC102641859
Capn5	Gm16938
Wnt11	Gm33770
Map6	Gm26705
Slco2b1	Gm34280
Kcne3	Gm34821
Pgm2l1	Gm34877
Ppme1	C2cd3
Coa4	LOC105243010
Arap1	Gm35219
Inppl1	LOC102639173
Folr1	Gm35597
Numa1	LOC105243016
Rnf121	Xntrpc
Art5	Art1
Prkcdbp	Gm36847
Smpd1	LOC105243022
Rrp8	Ilk
Pde3b	4933406I18Rik
Sox6	Sox6os
1110004F10Rik	1700003G18Rik
Rps13	LOC102637815

Smg1	4930583K01Rik
Gm35083	Gm35309
Knop1	Iqck
Eri2	2610020H08Rik
Eef2k	Gm36449
Rbbp6	4930413G21Rik
Atxn2l	LOC105243053
Cln3	Apobr
Ino80e	Hirip3
Cdipt	D830044I16Rik
Prrt2	LOC105243056
Zfp553	Gm4532
Tial1	Gm32816
Ate1	LOC102636907
Tacc2	LOC105243067
Plekha1	Gm5602
4933402N03Rik	1700029B22Rik
Gpr26	Gm34725
Ctbp2	LOC102639040
Tex36	Gm35625
Nkx6-2	Gm33031
Cfap46	Gm32680
Odf3	C330022C24Rik
B4galnt4	LOC105243088
Hras	Lrrc56
Lmntd2	Rassf7
Deaf1	Tmem80
Cd151	Gm10575
Brsk2	Gm20501
Dusp8	Gm32786
Gm10013	B130016D09Rik
Cars	LOC105243095
Ano1	D930030F02Rik
Fgf15	Gm26793
Xab2	Pet100
Pcp2	Gm30415
Trappc5	BB094273
Elavl1	Ccl25
Col4a1	Col4a2
Tubgcp3	LOC105243122
Atp11a	Gm33587
Mcf2l	Gm33637
Mcf2l	LOC105243124
Grtp1	2810030D12Rik
Dcun1d2	Tmco3
Erich1	LOC105243129
Arhgef10	Gm16350
Xkr5	LOC105244942
Ccdc70	Gm29821
Atp7b	Alg11
Ckap2	Gm29878
Slc25a15	1810012K16Rik
Vdac3	Gm30086
Ank1	LOC105243139
Ash2l	Kcnu1
Hook3	Rnf170
Zfp703	LOC105243151
Nrg1	Gm33223
Nrg1	LOC102641860
Ubxn8	Gm33433
Tnks	LOC105243167
Ppp1r3b	LOC105243169
Dlc1	LOC105243174
Msr1	Gm36741
Cnot7	Vps37a
Mtus1	Gm16193

Fgl1	Gm16348
Acsl1	Gm31050
Irf2	Gm16675
Stox2	Gm31404
Hand2	LOC102636514
Sap30	2500002B13Rik
Mfap3l	Gm34730
Clcn3	B230317F23Rik
Zfp930	LOC105243203
Atp13a1	LOC105243205
Mau2	Sugp1
Comp	Gm34002
Tmem59l	LOC105243207
Uba52	4930522P08Rik
Gdf15	Gm34154
Pik3r2	2010320M18Rik
Haus8	Myo9b
Ankle1	LOC105243212
Ano8	Gtpbp3
Nxnl1	Slc27a1
Calr3	1700030K09Rik
Slc35e1	LOC102639683
Nr3c2	LOC102640923
Nr3c2	Gm10649
Tmem184c	LOC105243221
Ttc29	LOC105243224
Rbmxl1	Slc10a7
Mmaa	Gm4890
Abce1	Anapc10
Hhip	LOC105243229
Il15	9530004M14Rik
Elmod2	Gm32327
Clgn	Gm32377
Dnajb1	LOC105246805
Rfx1	C330011M18Rik
Ier2	Gm26664
Nacc1	Trmt1
Dand5	Gadd45gip1
Calr	1700122E12Rik
Gcdh	LOC105243239
Asna1	2310036O22Rik
Vps35	Orc6
Dnaja2	Gm33428
Itfg1	Phkb
Cbln1	Gm2694
Cnep1r1	Gm2716
Nkd1	Gm35162
Sall1	Gm3134
Rpgrip1l	Gm36163
Irx3	Irx3os
Irx5	Crnde
Lpcat2	Gm36922
Gm26843	LOC105243254
Gm26843	Gnao1
Bbs2	LOC105243257
Mt2	LOC105243258
Kifc3	Gm31224
Kifc3	Gm31036
Tk2	Cklf
Rrad	Gm33023
Fam96b	LOC105243269
4931428F04Rik	LOC102636360
Exoc3l	E2f4
Fhod1	Slc9a5
Kctd19	Lrrc36
Ctcf	Gm5914

Acd	Pard6a
Slc7a6os	Prmt7
Smpd3	4930506A18Rik
Vps4a	Gm16208
Cog8	Nip7
Wwp2	LOC105243277
Pmfbp1	LOC105243283
Dhx38	Txnl4b
Phlpp2	LOC102638694
Chst4	8030455M16Rik
Hydin	Gm26832
Il34	Gm15894
Sf3b3	Cog4
Cntnap4	Gm26994
Vat1l	Gm30272
Wfdc1	Gm32352
Gse1	A330074K22Rik
Gins2	Gm10614
Foxf1	Fendrr
1700018B08Rik	1700030M09Rik
Banp	LOC105246826
Zfpm1	LOC105243311
Zc3h18	LOC105243312
Rnf166	Ctu2
Galns	Trappc2l
Acsf3	LOC105243314
Spg7	Gm35677
Spata2l	4933417D19Rik
Vps9d1	Zfp276
Spire2	Gm35738
Afg3l1	LOC105243318
Rhou	Gm35874
Ccsap	Gm9901
Acta1	Gm29773
Ttc13	Arv1
Exoc8	Sprtn
Map10	Gm30871
Pcnxl2	LOC102632990
Nrp1	LOC105243337
Gucy1a2	LOC105244989
Mtmr2	LOC105243343
Cep57	Fam76b
Amotl1	Gm34263
Amotl1	Gm7607
Ankrd49	Mre11a
4931406C07Rik	Taf1d
Ccdc67	4930540M03Rik
Cdc37	Gm35977
Gm36118	Pde4a
Slc44a2	LOC105243355
Ilf3	Gm36198
Qtrt1	Gm16853
Smarca4	LOC105243357
Ldlr	Gm26511
Tmem205	Ccdc159
Rgl3	Gm36414
Acp5	LOC105243359
Zfp599	LOC105243360
Tbx20	Gm29824
B3gat1	LOC102641308
Acad8	Thyn1
Opcml	LOC105243368
Opcml	LOC105243367
Gm30933	Snx19
Foxred1	Srpr
Acrv1	1700027I24Rik

Stt3a	Gm26787
Ubash3b	Gm35657
Tbcel	Gm16322
Grik4	Gm36648
Pvrl1	LOC102631930
Thy1	LOC105243388
Usp2	LOC105243390
Mfrp	LOC102632388
Mcam	Gm10687
Trappc4	Rps25
Kmt2a	LOC105243391
Atp5l	Gm30934
Rnf214	Pcsk7
4931429L15Rik	LOC105243395
Dlat	LOC102635122
Alg9	LOC102635198
Gm684	LOC102635638
Gm684	Gm32819
1810046K07Rik	LOC105243408
Arhgap20	LOC105245005
AI593442	Gm1715
Rfpl3s	Gm7444
Pstpip1	Gm31261
Lingo1	Gm31586
Sin3a	Gm10658
Sin3a	2700012I20Rik
Man2c1	Man2c1os
Cplx3	Gm32392
Clk3	Gm32510
Islr2	1600029O15Rik
Tbc1d21	1700072B07Rik
Parp6	LOC105243419
Lrrc49	B930082K07Rik
Tle3	Gm34157
Paqr5	Gm35152
2300009A05Rik	Gm16759
Iqch	Aagab
Zwilch	Rpl4
Dpp8	LOC105243437
Tln2	LOC105243440
Foxb1	B230323A14Rik
Fam63b	Gm31872
Lipc	Gm32017
Aqp9	Gm32511
Aldh1a2	Gm3458
Rfx7	4930509E16Rik
Ccpg1os	Ccpg1
Pigb	2310009A05Rik
Wdr72	Gm33407
Mapk6	4933433G15Rik
Cd109	Gm34887
Tmem30a	4930429F24Rik
Bckdhb	Gm36278
Tpbg	9330154J02Rik
Ube2cbp	Dopey1
Plscr2	Gm31700
Paqr9	LOC105243503
1700065D16Rik	Atr
Xrn1	Gm16794
Copb2	4930579K19Rik
Foxl2	Foxl2os
Mras	LOC105243512
Ppp2r3a	LOC105243517
Nphp3	Gm33054
Nek11	Aste1
Atp2c1	LOC105245059

Alas1	LOC102636338
Iqcf6	LOC105243524
Cyb561d2	Nprl2
Rassf1	Gm34106
Sema3f	Gm34454
Ip6k1	4930535L15Rik
Rnf123	Mst1
Ndufaf3	Dalrd3
Celsr3	Gm35025
Fbxw23	Gm34888
Nme6	3000002C10Rik
Dhx30	Gm10615
Tmie	Als2cl
Rtp3	Gm10030
Mlh1	Epm2aip1
Arpp21	2900079G21Rik
Susd5	Gm36883
Osbpl10	Gm26962
Tgfbr2	LOC105245082
Rbms3	LOC102635502
Cmc1	Gm17399
Wdr48	Gm2449
Csrnp1	Gm33800
Eif1b	D830035M03Rik
Ctnnb1	4930593C16Rik
Ulk4	Gm34655
Trak1	LOC105245086
Zfp651	E530011L22Rik
Pomgnt2	LOC105243578
Usp27x	Gm14379
Gripap1	LOC105243648
Hdac6	LOC105243649
Porcn	Gm33135
Gm6592	Gm14502
Srpx	Gm33844
Med14	Gm14634
Efhc2	Gm35105
Uxt	A230072C01Rik
Gm2913	LOC105247082
Zcchc12	LOC105247279
Akap14	LOC105243672
Rhox3f	LOC102642845
Actrt1	4930515L19Rik
Utp14a	LOC105243680
Aifm1	LOC105243681
Enox2	LOC105243682
Hs6st2	Gm35727
1700080O16Rik	Gm35790
Zfp449	LOC102642677
Mmgt1	Gm36239
Fmr1	Gm10474
Xlr5a	Gm14685
Slc6a8	Gm32261
Dnase1l1	Taz
Plxna3	LOC105243692
Gm6897	LOC105243693
Gm4937	LOC102636349
Gspt2	LOC102640328
LOC102634296	8030474K03Rik
Cited1	Gm14858
Dmrtc1a	1700011M02Rik
Zdhhc15	LOC105243714
Brwd3	2810403D21Rik
Gm6377	Gm34464
Pcdh11x	Gm30577
Pcdh19	Gm26851

Armcx4	B230119M05Rik
Wbp5	LOC105243722
Morf4l2	BC065397
Alg13	Gm33235
Fam120c	A230072E10Rik
Gpr173	3010001F23Rik
Mageh1	9530051G07Rik
Ptchd1	LOC102639025
Phka2	Gm15241
Mospd2	Fancb
