# Supplementary material for: Important cardiac transcription factor genes are accompanied by bidirectional long non-coding RNAs
Source: BMC Genomics. 2018 Dec 27;19:967. doi: 10.1186/s12864-018-5233-5 (PMC6307297; doi:10.1186/s12864-018-5233-5)
Supplement: Supplementary file 8 — The alignment of mouse Tbx5ua (isoform 2) and its human homolog (RefSeq: NR_038440.1) produced by EMBOSS water. The sequence shown in red is highly conserved as determined by EMBOSS Matcher. The sequences are highly conserved at the 5′ side. (PDF 158 kb) [file 12864_2018_5233_MOESM8_ESM.pdf]

```
Human    1 GCCGGG-------TCTGCGCAGCCACAGGTTCCGGACGT----CTTGGC-   38
           ||.|.|       |.|...||| |.|.||.|||.|..|.    |..|||
Mouse    2 GCAGAGGGAATTTTTTTTTCAG-CGCCGGATCCTGCAGAGGGGCGAGGCG   50

Human   39 CCCGGGAATAAATAAAGACATAAACCAACCCGGCTTTCTCCGGAGGAATG   88
           |.|.....|...|...|.   |||.|..|.....|||||
Mouse   51 CTCTATTTTGTTTTGTGG---AAATCGGCAATTATTTCT-----------   86

Human   89 AGGtctgatcaatgggcacaatttctaagcag-cgcagtggtggatgctc  137
            .||..||||||||||||.|||||||.||..| ||    |||||.|...|
Mouse   87 -AGTTGGATCAATGGGCATAATTTCTGAGAGGTCG----GGTGGGTCTGC  131

Human  138 gcaaacttttgcgcaccgctggaaacccactagg--ttgagttgcaaaac  185
           |.||.|||.|.|||.|.|...||||||||||.|.||  |||||||.|.||
Mouse  132 GTAAGCTTCTACGCGCATTTGGAAACCCATTGGGTTTTGAGTTACGAA--  179

Human  186 gtaccgcgtaga--------cgcccctggtggcgccgagagaagagctag  227
             |||||..|||        |.|||| .|.|||.|||.|..|..||||||
Mouse  180 --ACCGCACAGACTTTCTCCCACCCC-AGCGGCTCCGGGCAATAAGCTAG  226

Human  228 gcctgcccagcacagagccggagagcgtcgggccttccggaaggGT--CG  275
           ||||||||..|||.||||..|.|||||||.||||||||.||.||.|  |.
Mouse  227 GCCTGCCCTACACGGAGCAAGCGAGCGTCAGGCCTTCCCGAGGGATTCCC  276

Human  276 ACGAG-ATGA----GTTC----------CTACT-----------------  293
           |..|| ||||    ||||          |.||.
Mouse  277 AGAAGCATGACCCGGTTCTGGTCGCAGTCCACCGAGGATTGGAGCGGCAG  326

Human  294 TGAC--------CTCT---------GAGCCGAGG----------------  310
           ||||        |.||         |||||.|||
Mouse  327 TGACACGGGCGGCCCTGCAGCGGTAGAGCCAAGGATTTCAGGAAGGTTCT  376

Human  311 ------------------------------------TGGGCC-----GGA  319
                                               ||||||     |||
Mouse  377 TTCCAATGTTTTATTCAGCTTCCAGCCTCTCAGCTTTGGGCCTTCAGGGA  426
```

```
Human    320 AACCGAGGCCTAGG------------CCCCGCCGGGG----CTGC-----   348
             ||..|..|.|||||            |.|.||.||||    |||.
Mouse    427 AAGTGTTGACTAGGGAGTACAAGGTTCTCAGCTGGGGTCCACTGAGGAGG   476

Human    349 ---AAGGAAAAGGG----GAAACTCC------GAGC--------------   371
                .|.||||||.|    |..|||||      ||||
Mouse    477 TAGGAAGAAAAGAGAGCTGCCACTCCTGCCTGGAGCTGAACAACAGGCCA   526

Human    372 -GTAGCGT------------------------------------------   378
              |.||.||
Mouse    527 AGAAGAGTTTGGTGTTGGATGTGACAGAGAAAACATTAAAGGAATATTCT   576

Human    379 -------CTTTTCCTTG--------------------------------T   389
                    ||.|||||||                                |
Mouse    577 GGTTGAGCTCTTCCTTGAATTTTCAGGATGGGTCTCATGTAGACCAGGCT   626

Human    390 G-----GTTCC--------------------------------TTTCTCC   402
             |     |||||                                ||.||||
Mouse    627 GGCTCAGTTCCCCGTGTTTGCCCAGGATGACCTTGAATGTCTGTTCCTCC   676

Human    403 GGCA--------TCCCGGACT--------------------GCGGG----   420
             .|||        |||.||.||                    |||||
Mouse    677 AGCATCTACCTCTCCAGGGCTGAGATTACAAGCATGAGGGGGCGGGTTGT   726

Human    421 --------------------------------CCCTGCAGCCACCTGGAC   438
                                             |||..|..|||.||   |
Mouse    727 TGCTGAGAACAGAACACTAAGCACTTGACATTCCCAACTCCCAACT---C   773

Human    439 CGGCATTCAAAGGA----------------TTCTGCAAGTC------CAG   466
             |  |||||...|.|                |||| ||||.|      |.|
Mouse    774 C--CATTCTCGGTATTTTTGTATTCTTGTTTTCT-CAAGACTGATGTCTG   820

Human    467 CTT---------CACAGACTGGCTTTCCCAGACGCTCCGAAGCCCGCAC-   506
             ..|         ||||.||...|.|.|.||.||.|.|..|..|.|.|||
Mouse    821 TATACACACACACACACACACACATACACACACACACACACACACACACA   870
```

```
Human    507 ------------CACGAA------------------------CAG-AATA  519
                         ||||.|                        ||| |.|.
Mouse    871 CACACACACACACACGCAGATATTTTAGGCTCATGAATCTGCCAGCACTT  920

Human    520 AAGG--AGAGACGAGAGATC----GCAAC---------TAGATTTGA---  551
             .|||  ..||||.|..||.|    |||.|         |.|..||||
Mouse    921 GAGGTTCAAGACTAATGAACTTTTGCAGCACGCCAGGGTTGCCTTGATGT  970

Human    552 GAATCCTC---GTTCTTTT--CCCCAATC-GTTCGGGCAG---TAAAC--  590
             ||.|||.|   |.||.||.  |.|||..| ||||.|.|||   .||||
Mouse    971 GAGTCCCCCTGGCTCATTCAGCTCCATACAGTTCTGCCAGAGCAAAACAG  1020

Human    591 ---------TCC--------------------------------------  593
                      |||
Mouse   1021 TCACATGTGTCCCTTGCTTTGTGGAAGCAGTTTCTCTGGGGGCGGGGGGG  1070

Human    594 -GGAGCC-------------------------------------GGCTAC  605
              ||||||                                     |.||||
Mouse   1071 GGGAGCCTGGGGGGTCACTGACCTGGGTCCCGGTCAGGCTCTCTGACTAC  1120

Human    606 AG---CGCGCATCCTC----------------CCTTCCTC----------  626
             ||   |.|||..||.|                |||||.||
Mouse   1121 AGAGTCCCGCCACCCCCACCCCACCCCGAGCACCTTCATCATTGGCCTCT  1170

Human    627 ----------------------CTTTC--GGAGAACTGTC--GCT-----  645
                                   |||||  |.|||.||||.  |||
Mouse   1171 TCATTGTTGGACCTGGGGAAGGCTTTCAAGCAGATCTGTTGGGCTAGGTA  1220

Human    646 -TTCCCGAAG---CTGCTCCC---AGCTGGGTT-----TTCT-------C  676
              ||.||.|||   |.||..||   ||....|||     ||||       |
Mouse   1221 CTTGCCAAAGAGTCAGCCTCCATGAGAAAAGTTCAGACTTCTTTGGGTAC  1270

Human    677 CCGAAGTTTATTTT--TCTCATTTTGCAAAATTCCTG-------------  711
             ||||..|.|.|.||  ||||...|||..|.   ||.|
Mouse   1271 CCGATTTCTCTCTTCCTCTCCCATTGACAG---CCAGCGAAGTGGGTAGA  1317
```

```
Human      712 ---CCCTGATTTTAATAC----TGCAACAGGACAGCAG-----AGG----   745
                  |||||.||||....|    .|||||.||....|||     |||
Mouse     1318 GGACCCTGGTTTTCTGGCCCTTAGCAACTGGGTTCCAGGATGAAGGATGA  1367

Human      746 -----TTCCT-CTTTAAAAA------------GT---CAAAACCCAAAC-   773
                    .||.| ||||..|||            ||   ||.|.||||..|
Mouse     1368 ATGAAATCTTGCTTTCCAAACCTAATGGTTGTGTGCCCATATCCCAGCCT  1417

Human      774 ----CAAA-----GGGGCC------CATTGC-------------------   789
                   ||||     |.||||      ||.|||
Mouse     1418 CTATCAAAGCCTTGAGGCCAGCACACACTGCCCTGGAGGCAGGAGGAAGG  1467

Human      790 ------------TGTTGATAATT------------------TTTTTCTTC   809
                           |||.|..||..                  ||...||||
Mouse     1468 GCTATACTCAGGTGTAGGGAACAGGAAGACAGACCCAGCAGTTAAACTTC  1517

Human      810 ACCTTCTCCT-----TCCGT-----------------TTTTAAGAAAGAC   837
               ||.|.|.|.|     |||.|                 ||..|.|..||.|
Mouse     1518 ACATCCACATCTTAGTCCCTGGCACCCCAGGTAGGAGTTGAATGGGAGCC  1567

Human      838 ACT-----------------TCCAAAACTACCCATTGTTTTCCTCTCAAA   870
               .||                 |||.|..|..|||  ||..|.|..|.||..
Mouse     1568 CCTTGTGTAGGGGAAGTTCCTCCCACCCACCCC--TGCATGCTGCCCAGT  1615

Human      871 AGAGAACTCGC----------------------------------TTTAA   886
               .|.|..||.||                                  .|.|.
Mouse     1616 TGTGGGCTAGCCTGCTGAGTGAGCCTGTGGGATAGGGCTATGAGGATCAC  1665

Human      887 AATGCAT--C---CCTTTCTATTTCTCTCCGGATATGTC----CCCA---   924
               ||.||.|  |   ||||.| .||.|.||||..||.|.||    .|||
Mouse     1666 AAGGCCTGACTGTCCTTAC-GTTCCCCTCCAAATCTCTCTATTTCCAGGA  1714

Human      925 -------CCTGAC-CCGGTGGCAACT----TGTGGGG----CGTGGG---   955
                      |..|.| ||..|.||..||    ||||..|    |.||||
Mouse     1715 GAAAAGGCAGGTCGCCATTAGCCTCTCCTCTGTGCTGATATCCTGGGGCC  1764
```

```
Human        956 --GGTAGTGAAAAATAGAAG-----------------------------T    974
                   |||||.|....|||||||                             |
Mouse       1765 CAGGTAGGGGGTCATAGAAGGGTCCCAGGCCATGCTCAGATCTGTACCCT   1814

Human        975 GTGGACGGGGCTG---GGAAAATGAATAAGAATTATAACAATAGAAA---   1018
                 |||..|..|||||   |.......||..|||.|||...|..||||||
Mouse       1815 GTGCTCAAGGCTGCCTGAGTCCAAAAGCAGATTTACTTCCTTAGAAAGGG   1864

Human       1019 ---TGA------------------TGACAAAA---------CTA------   1032
                    |||                  ||.|.|||         |||
Mouse       1865 AATTGAGAGTTTAGTCCAGCTCAGTGCCTAAAATGGAGGCTCTAGGGTCC   1914

Human       1033 -AACTTT---GACCAAT---TCAATTTGCAAAAGAAAGGGTTGGTCTG--   1073
                  .|||.|   |.||..|   ||.|||..|...|...|||.|  |.||.
Mouse       1915 CCACTGTCAGGTCCTGTCCCTCTATTCCCTGCACCTAGGCT--GCCTTAC   1962

Human       1074 --------AGGGTGAGACATTCCCTGG------TT-----TCTCAAG---   1101
                         ||||...|.|  |||||||      ||     ||||.||
Mouse       1963 CACCCTACAGGGAATGTC--TCCCTGGGAAGTCTTCTAAATCTCCAGGAT   2010

Human       1102 -GGGGTCAC--------------------TTTCCCTCTCA--CCGAGTTA   1128
                  ||||.|.|                    ||.|||.|.||  ||..||..
Mouse       2011 TGGGGCCTCCAAGTCTCCTTCTGTAAACATTGCCCACACAGCCCTTGTAT   2060

Human       1129 AAT---CTGG--------CTCTGTTATATTTTTTCCAGGAACACAGTATG   1167
                 ..|   ||||        |.||....| |||.|||..|..|..||...|.
Mouse       2061 GCTGTACTGGGGGTGGAACCCTAGGGT-TTTATTCATGATAAGCAAGCTT   2109

Human       1168 TCTC-TTCCCA-------ATGTT--------TTATTCGG-----------   1190
                 |||| ||||||       .||||        ||.||.||
Mouse       2110 TCTCATTCCCAGCCTCTGCTGTTGTGCTTGGTTTTTTGGCATTTTTTAAT   2159

Human       1191 -TTTCTGGTTT----------------------CCC---------CACTT   1208
                  |||.||||||                      |.|         |.||.
Mouse       2160 TTTTTTGGTTTTGTTGTTGTTGTTTTTTTATGACACAGGGCCTCGCTCTG   2209
```

```
Human        1209 TTGACCGGAAGTATGTGG--------GAAGAATACTGGCTG---------   1241
                  |.||||.|..     |||        ..||||..|||.|||
Mouse        2210 TAGACCAGGC-----TGGCCTCACACTCAGAAATCTGTCTGCCTCTGCCT   2254

Human        1242 --TTATTATTATGATT-ATTGAGTGTCCAATACTCTCCT---CCAATATT   1285
                    ..|.||.|..|||| |.||.|||   |||...|.|||   |||...|.
Mouse        2255 CCCAAGTACTGGGATTAAATGTGTG---AATCACCACCTTGCCCAGCCTA   2301

Human        1286 TC---CACCAAGAATTATTATCGCT---------GC-------TCT----   1312
                  ||   .|.||..|.||||.||.||.         ||       |||
Mouse        2302 TCGTTGAACATTATTTATAATTGCATGTCATTTGGCAAGGGAGTCTGAGC   2351

Human        1313 C----------CTCAT--------------------------------TC   1320
                  |          |||.|                                ||
Mouse        2352 CATTGTGTGGGCTCTTGAGGTATTTAAATATATATATTTAAATAGGTGTC   2401

Human        1321 ATGT-----TAGTGTTGCT--------TTGTGTGCTT--TAGATG-----   1350
                  |..|     |.||||.|||        .|||..||||  |.|..|
Mouse        2402 AATTTGGAGTTGTGTAGCTATATCATCATGTAAGCTTCCTTGGAGTATTA   2451

Human        1351 --ACAATCGTCCTC------------------------------------   1362
                    |||..|.|||.|
Mouse        2452 AAACATGCATCCACCTATAATATGCACACATACATTTGAGTATATATTTA   2501

Human        1363 ----TTTACT-----CTCCAAGTTTGGAGG---GTGAGGGTTTTT---TG   1397
                      |||.||     |||...||.||.|..   |||.|.|.|.||   |.
Mouse        2502 TTATTTTCCTTGTACCTCATTGTATGAAAACATGTGTGAGCTATTGTGTA   2551

Human        1398 TTGTGG--TGG-----TGGTGGT----------------TGGGT---TCT   1421
                  |.|.||  |||     .||||||                ||.||   ||.
Mouse        2552 TAGGGGCATGGGGCACGGGTGGTATAGGATGATCATGTATGTGTCTGTCA   2601

Human        1422 TCTT--------------TGTTTTTGTTTTTTTGT-------TTTTCCTG   1450
                  ||||              |||.|.||..|.|.|||       |||..|||
Mouse        2602 TCTTGGAAGGGCCATGTATGTATATGCATGTGTGTGTGTGTGTTTATCTG   2651
```

```
Human      1451 TTCG---GGGTAA-TGAAATGAGAACTGGAAGAGAGGATAT---------  1487
                |..|   |.|||| ||...||.||  ||..||.|.||.|.|
Mouse      2652 TGTGTGTGTGTAAGTGGGGTGGGA--TGTGAGTGTGGGTGTGTGACGTAT  2699

Human      1488 -CA------TTTCGAGGCCCAGCTGAGAAGGA---------------TCC  1515
                 ||      |.||...|..||.|| .||||||               ||.
Mouse      2700 GCATGTGGGTATCAGAGGTCAACT-TGAAGGAGTCATGTCTCTTTTTTCA  2748

Human      1516 CAATGCGAAAAAT-----TTACACACTTCAAAGAGCAGAAAGGTGGAAGC  1560
                |.|||.|.....|     ||  ..||||..|..|.||||..  |.|.|||
Mouse      2749 CCATGTGGGTCTTGGGGATT--GAACTTAGATCACCAGACT--TAGCAGC  2794

Human      1561 GAGTGGTGCTTGGACACAAGGATGC--------CACTGTTCTTATC-TCC  1601
                .||.  ..|||...|.|..|..|.|        |.|..||.|.||| ||.
Mouse      2795 AAGC--CCCTTTACCCCTTGACTCCTCCCATCTCTCCATTTTAATCATCT  2842

Human      1602 TCCAATTTGGGAAG--TGCC--CAGCCTAGAAAGGTGAGGGTC-----C-  1641
                .||..|.||...||  ||||  |||||.....|||||.|||.|     |
Mouse      2843 ACCTTTATGATTAGTATGCCATCAGCCATTCCAGGTGTGGGGCTGAAGCT  2892

Human      1642 --CATCTGGGAGAC------CATGGAGTAC--CC---------CCAGG--  1670
                  |.|||||.|..|      |.||..|.||  |.         ||||.
Mouse      2893 GGCCTCTGGAACTCAGTTCTCTTGAGGCACGGCATTCTAGAGGCCAGAAG  2942

Human      1671 ----GTC-ACACTGAGCCTAAGAACTTCAACCATCCTG-----------C  1704
                    ||| |||...||||...|..||.|  ||...||.           .
Mouse      2943 GGATGTCTACAGACAGCCAGGGGCCTCC--CCTGTCTATATTTGGAAACA  2990

Human      1705 AGGTCAAAACCCACTGATAGG--TCT---CCCAAAATGTTTGGG----GT  1745
                |.|||.|.|.|||||....||  .||   |..||..||..||.|    ..
Mouse      2991 AAGTCCACAGCCACTTCCTGGGAACTGGGCAAAAGGTGCCTGAGAAAAAA  3040

Human      1746 AGGGGGAAGAAACATAGGGC-----ATTCCAGGT-CTGTAACCC-TGCAA  1788
                |.||||.....|..||.|||     .|.||...| ||||..||| ..||.
Mouse      3041 AAGGGGCGAGTATGTATGGCCGAGTGTGCCTCTTCCTGTGCCCCAAACAT  3090
```

```
Human  1789 GAGAG-AGACTCAAACTAAT-----------ATGTCTAGGTCTGGGGTGA  1826
            |.|.| ||.|.|.||||..|           |.|.|.||||.|..||||.
Mouse  3091 GTGGGAAGCCCCCAACTCCTGGCAGGCGCGCAGGACCAGGTGTTAGGTGC  3140

Human  1827 AGGTGGGGGGCCTGTCTGGATACCTC--------CTCCCT-------CAA  1861
            |..|.||     |||.||....|..|        |.|.|.       |||
Mouse  3141 ACATAGG-----TGTATGATGTCAACAGTTAAAGCACACAGTGGCTGCAA  3185

Human  1862 TTGAAGGG------------CTC--GTTGGAAACATAGGAACCCACTGCT  1897
            |.|..||.            |||  |..|||.|||.||.|.||| |..||
Mouse  3186 TGGGGGGACAGAGTGACATCCTCCAGACGGACACACAGAATCCC-CATCT  3234

Human  1898 CTCTGGGACTCTGGAAAGACAAATTTTCT-TTGGCAAACGGAACAGTGTC  1946
            ..|...|..||       .||...||.|| .||||.|.|  ..||||
Mouse  3235 TCCATAGCTTC-------CCAGGCTTCCTCCTGGCTACC--TCCAGT---  3272

Human  1947 GCGCTGTCGACAGAGCAGACTATTTTTC-------TTGTTC-----CCTC  1984
            ||.|.| |..||| ||||.||..||..|       .|||||     ||||
Mouse  3273 GCTCAG-CTCCAG-GCAGCCTTGTTCCCAGTGAGAATGTTCAGGGACCTC  3320

Human  1985 ACAAG-----GTGGGGGTGGGGGGAAAGAAAAGGAAGCAAGAAAGAAAGG  2029
            .||.|     ||.|..|..|..||.||...|..|...|||||.......|
Mouse  3321 TCAGGAGCCAGTAGATGCTGATGGCAAACCACTGTTTCAAGATGTCTCTG  3370

Human  2030 GAAATTAAATCCAC----ACGTGTT---------AAGCTCGCTCAAAGG-  2065
            |...|....|||.|    .||||.|         |||||..||||...|
Mouse  3371 GTCTTATCTTCCTCTGCTCCGTGGTGTTAGAAGAAAGCTATCTCACTAGC  3420

Human  2066 -GCCAG-AGTCAAAACCAGAGATGAAACTTCGACTCCAGTTCTGGCTGCC  2113
             .|||| ||.||..|||.......|.|| .| ||.||    ||||.|| |
Mouse  3421 TTCCAGTAGCCAGGACCCCCCCACACAC-AC-ACACC----CTGGTTG-C  3463

Human  2114 CGCGGGC---CTCTTTC-CAACCAG----CCAGGCCTCACAGAGCAAGGC  2155
            |.|..||   |.|||.| ||.|.||    .||.||..||.|..||.|.|.
Mouse  3464 CACCAGCTTGCACTTCCTCAGCAAGGGAATCAAGCAACAAAATGCCAAGG  3513
```

```
Human  2156 ATCCC---CAC-TGGAGAGAATTCGAT--------CTG---ATGTTTCAT  2190
            | |.|   ||| |.|||.|.|..||.|        ||.   ||||.||||
Mouse  3514 A-CACTGTCACTTAGAGCGCACGCGGTACATGTAGCTTAGCATGTCTCAT  3562

Human  2191 AAAGCGCTTTATTTAAGGAGAAGGGGCATGGGTCTGTCTCCAAGTCTGTG  2240
            .|| |.|.|||....|..|....|||||     .|||..|||.|.|.|||
Mouse  3563 GAA-CACATTAACACATAACGCAGGGCA-----ATGTAGCCATGCCCGTG  3606

Human  2241 CTGCAGAAAT--CCAGAATTTGTAGCGGCAAAGAAA--------ACAAAG  2280
            .| |||...|  ||||.....|..||......||.|        |||.||
Mouse  3607 AT-CAGTCCTAGCCAGCCACAGATGCAAACCTGAGACTTTTTGGACAGAG  3655

Human  2281 GAGAT-----AACAATTGTAATCATAATGACC--CTCTTTATGAAAACTC  2323
            |.|||     |||......|.|||..|.||.|  |||.||.....|.|||
Mouse  3656 GCGATTCGGAAACGTGCACACTCAGGAGGAACTTCTCCTTGCCCGACCTC  3705

Human  2324 A-TCTTGATGTCCTCCAAATCAAAACAACAATTCAGGCAAT--ATTCAAG  2370
            | .|..|.|..||.||.||.|...||..|    |.|||..|  ..||.||
Mouse  3706 ACACCCGGTCGCCACCCAACCCCCACCCC----CCGGCCTTGGGGTCCAG  3751

Human  2371 CA---------------ATGGCCA-AAATCATC--CAGGCC--ATTAAAT  2400
            ||               |.||.|| .|||||.|  |.||||  |||||||
Mouse  3752 CACACTGCACAACTGGGAGGGACACCAATCAGCCGCCGGCCATATTAAAT  3801

Human  2401 -----CATC-CAG---GCCATTGTTTTACAAAATTCCTAAGGT-------  2434
                 ||.| |||   ||.|..|..|||..|.|..||..|.||
Mouse  3802 GTCTACAACGCAGTGTGCAAGGGGGTTAGCACACCCCACACGTAAGTGCA  3851

Human  2435 ---------------CTGATCAGG--CTTTCGGACTTGG-----------  2456
                           |.|.|||..  |.|||...|.||.
Mouse  3852 CGCACACCTTGAATCCCGTTCATACCCATTCATTCATGATTTATACATCT  3901

Human  2457 ------GCCTAGC-TGGGTCAAGAGAACCTGAAATGCTGTTGTCAAGAGA  2499
                  |||...| ||||.|.    |||||   .|.|..||.||.|||.|
Mouse  3902 ACATATGCCACACGTGGGCCT----AACCT---CTTCCCTTATCCAGACA  3944
```

```
Human          2500 CTC-----TGA-AATCAC---CAGTTT-----AATC-------AGGACAG   2528
                    |||     ||| ..||||   |||.||     ||||       ||...|.
Mouse          3945 CTCTCCCTTGATGCTCACAGGCAGGTTTGGTAAATCCATCTTAAGAGAAT   3994

Human          2529 CTAAT--CTC-----------TACATCCTTGAGGCCTCTCTTTTGG--GG   2563
                    ||..|  |||           |.||.||....|||.|.|.||.||  ||
Mouse          3995 CTGTTGACTCAATAGATCTTGTCCAGCCTGAGAGCCACCCATTGGGGAGG   4044

Human          2564 AGAGACTTTCTTGAG---AGAGGACA-------------CTACAGGA-CT   2596
                    |.|||....|.||.|   ||.|..||             |..|.|.| ||
Mouse          4045 AAAGATGGACATGTGGACAGGGAGCAGATAGAATGAGTCCATCGGTACCT   4094

Human          2597 CAGCT--------ACAGAGCTCTGC--TATACATGGGTGGGAGTGG----   2632
                    |.|||        ||....|.||||  |.|.|..|..|...||..|
Mouse          4095 CCGCTTTCAGCCCACCTTTCCCTGCCTTCTGCCAGCCTCACAGAAGCTCC   4144

Human          2633 -AGGCC-----GC----CACCCCA--------CCTGGAAC--TGTGAG--   2660
                     .||||     ||    ||.||||        ||.|||||  ||||.|
Mouse          4145 AGGGCCTTAAAGCAAATCAACCCATTTTGACCCCGGGAACCATGTGGGCT   4194

Human          2661 -TGAAAACTCTAGTAA-----------TGAT----CTTGAGGAGGTGAAG   2694
                     .|.||||.|..||.|           ||||    ||..||||||...||
Mouse          4195 CAGCAAACACAGGTCAGACGGAGTCCTTGATTCTGCTCAAGGAGGGATAG   4244

Human          2695 GGAGGTAGGTGG---GGGTGTGGGTG-----ATGAGG--GAGGCCA----   2730
                    |.|..|||..|.   ||.|.|..|.|     .|||||  |||||||
Mouse          4245 GCATATAGAGGACCCGGTTTTAAGAGCCTACCTGAGGCTGAGGCCAGCAT   4294

Human          2731 -----AACCCCAAT-CTGGAAGGATC----TGTCACT----GAAACAATT   2766
                         .|||||..| |..|....|.|    ||.|.|.    |.||||
Mouse          4295 CCTCTGACCCCTGTACAAGCCACACCACAGTGACTCCTCAGGGAACA---   4341

Human          2767 GCTTGGTCGAGACCTCTAATTTTTCTTCCTCTCCAG-----CCAG---TC   2808
                     |.||.|| |..|||| .|....||.|||.|.||||     ||||   ||
Mouse          4342 -CCTGATC-ACTCCTC-CACCCATCATCCCCGCCAGCTCCTCCAGGGATC   4388
```

```
Human  2809 TC--------CTG------------------------------TGGC---  2817
            ||        |||                              ||.|
Mouse  4389 TCTGAGCTGACTGTTTAGGTACTATCAAAAGATGCCACATGATTGTCATG  4438

Human  2818 TCCC------TGGTGCT-----GGGTTTCT---GGCCTTTGGTG------  2847
            ||||      |||..||     .||..|||   ||.|||...||
Mouse  4439 TCCCTGCATATGGCCCTCCAAAAGGCATCTGAAGGTCTTCCATGAAATTC  4488

Human  2848 -ACATCCTAACA----GAAGGGT---CCGGAAGCAG------GGAC----  2879
             ||.|||..|||    |||||.|   |.||...|.|      ||.|
Mouse  4489 CACCTCCACACATCCTGAAGGTTGTGCTGGCTCCTGTCTGTTGGTCCCAC  4538

Human  2880 ------CCCAGACACCTTGC---TCCCTCTCCCAAATC-----------A  2909
                  |||||.|.||||.|   ||||..||||....|           .
Mouse  4539 CTCCCACCCAGCCCCCTTTCTGTTCCCCATCCCTGCACCTTCTTTTCCTT  4588

Human  2910 CACCCAAAAC-ATACTGGGGTGAAACAACTGACAGAGGGTCTCTGTGGTG  2958
            |.||||..|| .|||...||||...|..|||.|...   |||||.||...
Mouse  4589 CCCCCAGGACTTTACACAGGTGGTGCCTCTGTCCTT---TCTCTCTGCCT  4635

Human  2959 AGCAACTG-ACTGAAGTTTCCAGTGGAAAAGTTCCTGACGATT--GTTTC  3005
            .||||||| .|||..|||  ||.||.....|||||.|.||.||  ||||.
Mouse  4636 GGCAACTGTTCTGTTGTT--CATTGCTGCTGTTCCCGCCGTTTGAGTTTA  4683

Human  3006 CGCAGGGACCTTGAATTTC----CTGGT-------CAC------------  3032
            .......|||.|...||||    ..|||       |||
Mouse  4684 TTTGTTTACCGTACTTTTCACATAAGGTAAAAATACACGTGCAATCAAAT  4733

Human  3033 ----CATGGCGACCTGGAAG------TCCCACC---------GA-ATGCT  3062
                ||||.|||..|||.||      ||..|||         || |||||
Mouse  4734 ATATCATGCCGATATGGCAGATGGACTCAGACCAGGGAATGTGAGATGCT  4783

Human  3063 CTC--------------------AACAAT-------GGGGT----ACCCC  3081
            |||                    |.||.|       |||||    ||.|.
Mouse  4784 CTCAGATAAGATATAATGAACGAATCAGTAATGTACGGGGTTGTAACACT  4833
```

```
Human   3082 GAGGACATTCGTGCAATTGGCTG--------CGCAGTCTGAGGCCTGAAA   3123
             .....|.|.|.|.||||...|..        .||||||.||....||..|
Mouse   4834 CTCTCCTTCCTTACAATAAACAAAACAAAACAGCAGTCAGAATGATGGGA   4883

Human   3124 AGC----------------------GTTTCTG------------CCAATC   3139
             |||                      .|.|.||            ||.|..
Mouse   4884 AGCTTGGAGCAAAAGATAAAGATATATATATGAAGGAGGGAACACCCAAA   4933

Human   3140 GTTCTTGACTTTCTGGAGC-CT---CCTTAGTGGGT-TCAACGGAGCAGG   3184
             |.||.. .||.||||...| ||   |||..||||.| |||.|.|. |..|
Mouse   4934 GATCCC-TCTCTCTGTTTCTCTGTGCCTGTGTGGCTCTCACCAGT-CCTG   4981

Human   3185 GATTTTTAAAACTGCCC----------------CCTTTTTTAAAC-----   3213
             .||||.|....||.||.                |||||.||..||
Mouse   4982 TATTTCTCTCCCTTCCATCCCTCATCTCTGGCTCCTTTCTTCCACCCTCT   5031

Human   3214 -------------------AGG---------GGAGAAAATTGAGATTT--   3233
                                |||         ||.||||.|.|...|||
Mouse   5032 GTCTCTTCCCAGGAGAAGAAGGCAGCTGATAGGTGAAAGTGGTATTTTTA   5081

Human   3234 ----------AG---------TACCAGGTGGGTTCAGG-TCCTGAGC---   3260
                       ||         ||.|..|...||||.|. ||.||..|
Mouse   5082 TAGGCTGATAAGCCCTCTCCTTATCTAGAAAGTTCTGTCTCATGCCCGCC   5131

Human   3261 ----AAAAGCCCCATCTT--------------TGAATTCAGAATGC----   3288
                 ||..|.|||..|||              |||| .|||.|.||
Mouse   5132 CCCTAAGTGTCCCTACTTGACTATTTACTCACTGAA-GCAGCAGGCATCC   5180

Human   3289 ---------TGACCCCAGGGCCGATCGTG---------TAG--------A   3312
                      |||||||.|..||.|.|.||         |.|        |
Mouse   5181 TGGGAAGGGTGACCCCCGCCCCCACCCTGCCCTGTCCCTGGGAGTCCTCA   5230

Human   3313 GC---CTATGG---TGTATCTTAAT-CGAAACCCGTGG------------   3343
             ||   ||.|||   .||..|.|..| |....|||.|||
Mouse   5231 GCTCACTTTGGCTCAGTCCCCTCCTGCTCCCCCCATGGAGGACTTACCCT   5280
```

```
Human   3344 ---CCCAAACATC--TCA------------GCCAGGAACCTGG-------  3369
                ||||..|.||  |||            |.||||...|.||
Mouse   5281 ACCCCCAGCCTTCCTTCACTCCCGAAAGGTGGCAGGTGACAGGAGATGGC  5330

Human   3370 --------GACT---TTAGGTGT-----------------CACTTAATCT  3391
                     |||.   .|.||.||                 |||....|||
Mouse   5331 GAGGGAGAGACAGGGATGGGGGTGGGGGTCTGTCTGTCCCCACAGGTTCT  5380

Human   3392 C----------TTC-------ACACCTG-----TTAAC-----TACACC-  3413
             |          |||       |||||.|     ||||.     |||.||
Mouse   5381 CTGTACCATTTTTCTTTAATGACACCAGCTATATTAAATTCTGTACTCCG  5430

Human   3414 ---TATC-----TGCATAGGAGGCTC--------CCC------------  3434
                |.||     |.|||....|||.|        |||
Mouse   5431 AAGTCTCTTAAATTCATTTATGGCCCATACTGGACCCTGAGCCATAATAA  5480

Human   3435 -------------------------ACATC--------AGTCCAG-----  3446
                                      |||||        .|.||.|
Mouse   5481 CAATAAAATAAAATCAAGCATTTATACATCCAGGGTGTTGCCCCGTAGCT  5530

Human   3447 ---------------GCAACTTTTTTCTTTGCCTG---GGGCCATT----  3474
                            ||..||...|.||||..|||   |||||.||
Mouse   5531 GGTAGAAAATATTGAGCTTCTAGCTGCTTTTGCTGGCCGGGCCCTTCATA  5580

Human   3475 ----------TCT----------TCTAAAT--------------------  3484
                       |||          |||.|.|
Mouse   5581 TCACCCACGCTCTGACCTTTTGGTCTCACTGGGTGGGGGCTTGAAGCAGT  5630

Human   3485 -----------GGAAGGAGAAAAG-------TCTGAGCA-------TTAG  3509
                        |||||||||...|       .|||||||       |||.
Mouse   5631 GGAGGGAGTAGGGAAGGAGAGCTGGGATCTTGCTGAGCACAGAGATTTAA  5680

Human   3510 A-----------------------------------------AATTGTGG  3518
             |                                         ||.|||.|
Mouse   5681 AAGCTGGAGAGCAAAGAGGTTTTTTTTTTCTGGGCTCAGCTCAACTGTAG  5730
```

```
Human           3519 CATTAATGTCT-----------------GCA---AGGGC-----------  3537
                     |.|...|.|||                 |||   .||||
Mouse           5731 CCTGTCTCTCTCTCTCTCTCTCTCTAAGGCACAGTGGGCCTGGGTACGGA  5780

Human           3538 -------TAATGGAGTGAAA----AGAATAC---ATTATTCTC-------  3566
                            ||.|||.|||..|    ||..|.|   ||.|..|||
Mouse           5781 GAGAGGTTACTGGGGTGGGAAGCCAGGTTGCAGAATGAAGCTCTTGGATG  5830

Human           3567 ---CTTT--------TCTTCTTAGAAAAAAATTTC--AATTGTGGATGTA  3603
                        ||.|        .||||.........|.||.|  |.|||||.||| .
Mouse           5831 TGGCTGTAGGTTCAAACTTCCACTCTGCCACTTCCCTAGTTGTGTATG-C  5879

Human           3604 ATAGAAATACCATTAAAAT--------------AATATTTCTGA------  3633
                     ..||...|.||.||....|              |||..||||.|
Mouse           5880 CCAGCCTTTCCGTTTCTGTATCTTGCTCTGTGCAATGATTCTCACTCCTC  5929

Human           3634 ---------TTGCAAAAAAAAAAAAAAAAAA  3655
                              |||.||||..|.|.||||.|||
Mouse           5930 AATGGGCAGTTGTAAAAGGAGAGAAAACAAA  5960
```
