# Supplementary material for: Important cardiac transcription factor genes are accompanied by bidirectional long non-coding RNAs
Source: BMC Genomics. 2018 Dec 27;19:967. doi: 10.1186/s12864-018-5233-5 (PMC6307297; doi:10.1186/s12864-018-5233-5)
Supplement: Supplementary file 9 — The sequence of each isoform of Tbx5ua as determined by cufflinks. Isoform numbers correspond to those in Fig. 3a. (PDF 46 kb) [file 12864_2018_5233_MOESM9_ESM.pdf]

Isoform 1

1 CGGGTGCACT GTGCAGAGGG AATTTTTTTT TCAGCGCCGG ATCCTGCAGA GGGGCGAGGC
61 GCTCTATTTT GTTTTGTGGA AATCGGCAAT TATTTCTAGT TGGATCAATG GGCATAATTT
121 CTGAGAGGTC GGGTGGGTCT GCGTAAGCTT CTACGCGCAT TTGGAAACCC ATTGGGTTTT
181 GAGTTACGAA ACCGCACAGA CTTTCTCCCA CCCCAGCGGC TCCGGGCAAT AAGCTAGGCC
241 TGCCCTACAC GGAGCAAGCG AGCGTCAGGC CTTCCCGAGG GATTCCCAGA AGCATGACCC
301 GGTTCTGGTC GCAGTCCACC GAGGATTGGA GCGGCAGTGA CACGGGCGGC CCTGCAGCGG
361 TAGAGCCAAG GTCAGGCCCC AAGGGACAGC GGGGCAGCAG CCTGCAGCAC AGATGCAGGA
421 CACCAGCGTG CATCCCTGTT CTCTTCCCCA AGAGTCCAGG CTGCCCGGCC TGGTGCTCCA
481 GCTTTGCTTT CGTTTCCCAG GCGAACGAGC CCCAAATTCA CGTTTTCACC CAAGAATGCA
541 ATCCAGAATC CTTGTCCCCG TATTCGAGTA GGGTGACCAG GAGAACCGAG GTGGGGGCAG
601 GAAACCCAGA CCGAGGGTGC CCCGCGAGTG GGCCGAGTTC CTTTCACTTT CCCCCATTGC
661 TCCGCAATTT CGGGTGCAAC TACCTGGGCC AAGCCCAGCT TCAGAGACTG GCCCCGTGCT
721 CTGGAGAAAG CAACCACCAC GGGAAGAGAC TGCAGCCTCA GCGTCTCCTT GTCCGCTTCA
781 CCCTCATCCT CTGTGCGGGA GAGGGCAGCA ACATACACGC CTGGCACCTT CCTTCTTTCC
841 AAGAGTTGCG CTTTCCTAAC CCTGCTCCCC GCTGGTTTTT CTCATACTTC CTTGCCCCCT
901 TTTGCAAAAT TTCCGCCTTG ATCTTAAATA CCTCGAAAGA TAGGAAGACC CGGAATTCTA
961 TTAAAACAAA ACAAAACGTA ATGAGCCATT GCTGTTATTT GTATTATTAC TATTGCTATT
1021 ATTAGTATTC GTATTATTGC TATTCCTTAC ATTTCCCC

Isoform 2

1 CGGGTGCACT GTGCAGAGGG AATTTTTTTT TCAGCGCCGG ATCCTGCAGA GGGGCGAGGC
61 GCTCTATTTT GTTTTGTGGA AATCGGCAAT TATTTCTAGT TGGATCAATG GGCATAATTT
121 CTGAGAGGTC GGGTGGGTCT GCGTAAGCTT CTACGCGCAT TTGGAAACCC ATTGGGTTTT
181 GAGTTACGAA ACCGCACAGA CTTTCTCCCA CCCCAGCGGC TCCGGGCAAT AAGCTAGGCC
241 TGCCCTACAC GGAGCAAGCG AGCGTCAGGC CTTCCCGAGG GATTCCCAGA AGCATGACCC
301 GGTTCTGGTC GCAGTCCACC GAGGATTGGA GCGGCAGTGA CACGGGCGGC CCTGCAGCGG
361 TAGAGCCAAG GGAGTACAAG GTTCTCAGCT GGGGTCCACT GAGGAGGTAG GAAGAAAAGA
421 GAGCTGCCAC TCCTGCCTGG AGCTGAACAA CAGGCCAAGA AGAGTTTGGT GTTGGATGTG
481 ACAGAGAAAA CATTAAAGGA ATATTCTGGT TGAGCTCTTC CTTGAATTTT CAGGATGGGT
541 CTCATGTAGA CCAGGCTGGC TCAGTTCCCC GTGTTTGCCC AGGATGACCT TGAATGTCTG
601 TTCCTCCAGC ATCTACCTCT CCAGGGCTGA GATTACAAGC ATGAGGGGGC GGGTTGTTGC
661 TGAGAACAGA ACACTAAGCA CTTGACATTC CCAACTCCCA ACTCCCATTC TCGGTATTTT
721 TGTATTCTTG TTTTCTCAAG ACTGATGTCT GTATACACAC ACACACACAC ACACATACAC
781 ACACACACAC ACACACACAC ACACACACAC ACACACGCAG ATATTTTAGG CTCATGAATC
841 TGCCAGCACT TGAGGTTCAA GACTAATGAA CTTTTGCAGC ACGCCAGGGT TGCCTTGATG

901 TGAGTCCCCC TGGCTCATTC AGCTCCATAC AGTTCTGCCA GAGCAAAACA GTCACATGTG
961 TCCCTTGCTT TGTGGAAGCA GTTTCTCTGG GGGCGGGGGG GGGGAGCCTG GGGGGTCACT
1021 GACCTGGGTC CCGGTCAGGC TCTCTGACTA CAGAGTCCCG CCACCCCCAC CCCACCCCGA
1081 GCACCTTCAT CATTGGCCTC TTCATTGTTG GACCTGGGGA AGGCTTTCAA GCAGATCTGT
1141 TGGGCTAGGT ACTTGCCAAA GAGTCAGCCT CCATGAGAAA AGTTCAGACT TCTTTGGGTA
1201 CCCGATTTCT CTCTTCCTCT CCCATTGACA GCCAGCGAAG TGGGTAGAGG ACCCTGGTTT
1261 TCTGGCCCTT AGCAACTGGG TTCCAGGATG AAGGATGAAT GAAATCTTGC TTTCCAAACC
1321 TAATGGTTGT GTGCCCATAT CCCAGCCTCT ATCAAAGCCT TGAGGCCAGC ACACACTGCC
1381 CTGGAGGCAG GAGGAAGGGC TATACTCAGG TGTAGGGAAC AGGAAGACAG ACCCAGCAGT
1441 TAAACTTCAC ATCCACATCT TAGTCCCTGG CACCCCAGGT AGGAGTTGAA TGGGAGCCCC
1501 TTGTGTAGGG GAAGTTCCTC CCACCCACCC CTGCATGCTG CCCAGTTGTG GGCTAGCCTG
1561 CTGAGTGAGC CTGTGGGATA GGGCTATGAG GATCACAAGG CCTGACTGTC CTTACGTTCC
1621 CCTCCAAATC TCTCTATTTC CAGGAGAAAA GGCAGGTCGC CATTAGCCTC TCCTCTGTGC
1681 TGATATCCTG GGGCCCAGGT AGGGGGTCAT AGAAGGGTCC CAGGCCATGC TCAGATCTGT
1741 ACCCTGTGCT CAAGGCTGCC TGAGTCCAAA AGCAGATTTA CTTCCTTAGA AAGGGAATTG
1801 AGAGTTTAGT CCAGCTCAGT GCCTAAAATG GAGGCTCTAG GGTCCCCACT GTCAGGTCCT
1861 GTCCCTCTAT TCCCTGCACC TAGGCTGCCT TACCACCCTA CAGGGAATGT CTCCCTGGGA
1921 AGTCTTCTAA ATCTCCAGGA TTGGGGCCTC CAAGTCTCCT TCTGTAAACA TTGCCCACAC
1981 AGCCCTTGTA TGCTGTACTG GGGGTGGAAC CCTAGGGTTT TATTCATGAT AAGCAAGCTT
2041 TCTCATTCCC AGCCTCTGCT GTTGTGCTTG GTTTTTTGGC ATTTTTTAAT TTTTTTGGTT
2101 TTGTTGTTGT TGTTTTTTTA TGACACAGGG CCTCGCTCTG TAGACCAGGC TGGCCTCACA
2161 CTCAGAAATC TGTCTGCCTC TGCCTCCCAA GTACTGGGAT TAAATGTGTG AATCACCACC
2221 TTGCCCAGCC TATCGTTGAA CATTATTTAT AATTGCATGT CATTTGGCAA GGGAGTCTGA
2281 GCCATTGTGT GGGCTCTTGA GGTATTTAAA TATATATATT TAAATAGGTG TCAATTTGGA
2341 GTTGTGTAGC TATATCATCA TGTAAGCTTC CTTGGAGTAT TAAAACATGC ATCCACCTAT
2401 AATATGCACA CATACATTTG AG

Isoform 3

1 CGGGTGCACT GTGCAGAGGG AATTTTTTTT TCAGCGCCGG ATCCTGCAGA GGGGCGAGGC
61 GCTCTATTTT GTTTTGTGGA AATCGGCAAT TATTTCTAGT TGGATCAATG GGCATAATTT
121 CTGAGAGGTC GGGTGGGTCT GCGTAAGCTT CTACGCGCAT TTGGAAACCC ATTGGGTTTT
181 GAGTTACGAA ACCGCACAGA CTTTCTCCCA CCCCAGCGGC TCCGGGCAAT AAGCTAGGCC
241 TGCCCTACAC GGAGCAAGCG AGCGTCAGGC CTTCCCGAGG GGAGTACAAG GTTCTCAGCT
301 GGGGTCCACT GAGGAGGTAG GAAGAAAAGA GAGCTGCCAC TCCTGCCTGG AGCTGAACAA
361 CAGGCCAAGA AGAGTTTGGT GTTGGATGTG ACAGAGAAAA CATTAAAGGA ATATTCTGGT
421 TGAGCTCTTC CTTGAATTTT CAGGATGGGT CTCATGTAGA CCAGGCTGGC TCAGTTCCCC

481 GTGTTTGCCC AGGATGACCT TGAATGTCTG TTCCTCCAGC ATCTACCTCT CCAGGGCTGA
541 GATTACAAGC ATGAGGGGGC GGGTTGTTGC TGAGAACAGA ACACTAAGCA CTTGACATTC
601 CCAACTCCCA ACTCCCATTC TCGGTATTTT TGTATTCTTG TTTTCTCAAG ACTGATGTCT
661 GTATACACAC ACACACACAC ACACATACAC ACACACACAC ACACACACAC ACACACACAC
721 ACACACGCAG ATATTTTAGG CTCATGAATC TGCCAGCACT TGAGGTTCAA GACTAATGAA
781 CTTTTGCAGC ACGCCAGGGT TGCCTTGATG TGAGTCCCCC TGGCTCATTC AGCTCCATAC
841 AGTTCTGCCA GAGCAAAACA GTCACATGTG TCCCTTGCTT TGTGGAAGCA GTTTCTCTGG
901 GGGCGGGGGG GGGGAGCCTG GGGGGTCACT GACCTGGGTC CCGGTCAGGC TCTCTGACTA
961 CAGAGTCCCG CCACCCCCAC CCCACCCCGA GCACCTTCAT CATTGGCCTC TTCATTGTTG
1021 GACCTGGGGA AGGCTTTCAA GCAGATCTGT TGGGCTAGGT ACTTGCCAAA GAGTCAGCCT
1081 CCATGAGAAA AGTTCAGACT TCTTTGGGTA CCCGATTTCT CTCTTCCTCT CCCATTGACA
1141 GCCAGCGAAG TGGGTAGAGG ACCCTGGTTT TCTGGCCCTT AGCAACTGGG TTCCAGGATG
1201 AAGGATGAAT GAAATCTTGC TTTCCAAACC TAATGGTTGT GTGCCCATAT CCCAGCCTCT
1261 ATCAAAGCCT TGAGGCCAGC ACACACTGCC CTGGAGGCAG GAGGAAGGGC TATACTCAGG
1321 TGTAGGGAAC AGGAAGACAG ACCCAGCAGT TAAACTTCAC ATCCACATCT TAGTCCCTGG
1381 CACCCCAGGT AGGAGTTGAA TGGGAGCCCC TTGTGTAGGG GAAGTTCCTC CCACCCACCC
1441 CTGCATGCTG CCCAGTTGTG GGCTAGCCTG CTGAGTGAGC CTGTGGGATA GGGCTATGAG
1501 GATCACAAGG CCTGACTGTC CTTACGTTCC CCTCCAAATC TCTCTATTTC CAGGAGAAAA
1561 GGCAGGTCGC CATTAGCCTC TCCTCTGTGC TGATATCCTG GGGCCCAGGT AGGGGGTCAT
1621 AGAAGGGTCC CAGGCCATGC TCAGATCTGT ACCCTGTGCT CAAGGCTGCC TGAGTCCAAA
1681 AGCAGATTTA CTTCCTTAGA AAGGGAATTG AGAGTTTAGT CCAGCTCAGT GCCTAAAATG
1741 GAGGCTCTAG GGTCCCCACT GTCAGGTCCT GTCCCTCTAT TCCCTGCACC TAGGCTGCCT
1801 TACCACCCTA CAGGGAATGT CTCCCTGGGA AGTCTTCTAA ATCTCCAGGA TTGGGGCCTC
1861 CAAGTCTCCT TCTGTAAACA TTGCCCACAC AGCCCTTGTA TGCTGTACTG GGGGTGGAAC
1921 CCTAGGGTTT TATTCATGAT AAGCAAGCTT TCTCATTCCC AGCCTCTGCT GTTGTGCTTG
1981 GTTTTTTGGC ATTTTTTAAT TTTTTTGGTT TTGTTGTTGT TGTTTTTTTA TGACACAGGG
2041 CCTCGCTCTG TAGACCAGGC TGGCCTCACA CTCAGAAATC TGTCTGCCTC TGCCTCCCAA
2101 GTACTGGGAT TAAATGTGTG AATCACCACC TTGCCCAGCC TATCGTTGAA CATTATTTAT
2161 AATTGCATGT CATTTGGCAA GGGAGTCTGA GCCATTGTGT GGGCTCTTGA GGTATTTAAA
2221 TATATATATT TAAATAGGTG TCAATTTGGA GTTGTGTAGC TATATCATCA TGTAAGCTTC
2281 CTTGGAGTAT TAAAACATGC ATCCACCTAT AATATGCACA CATACATTTG AG

Isoform 4

1 CGGGTGCACT GTGCAGAGGG AATTTTTTTT TCAGCGCCGG ATCCTGCAGA GGGGCGAGGC
61 GCTCTATTTT GTTTTGTGGA AATCGGCAAT TATTTCTAGT TGGATCAATG GGCATAATTT
121 CTGAGAGGTC GGGTGGGTCT GCGTAAGCTT CTACGCGCAT TTGGAAACCC ATTGGGTTTT

181 GAGTTACGAA ACCGCACAGA CTTTCTCCCA CCCCAGCGGC TCCGGGCAAT AAGCTAGGCC
241 TGCCCTACAC GGAGCAAGCG AGCGTCAGGC CTTCCCGAGG GGAGTACAAG GTTCTCAGCT
301 GGGGTCCACT GAGGAGGTAG GAAGAAAAGA GAGCTGCCAC TCCTGCCTGG AGCTGAACAA
361 CAGGCCAAGA AGAGTTTGGT GTTGGATGTG ACAGAGAAAA CATTAAAGGA ATATTCTGGT
421 TGAGCTCTTC CTTGAATTTT CAGGGCCAAT GGGATCTGTC TTCTAGTCCG GTTTGCCACA
481 ACCAAGAAAG TTTAAGCTCA ACAAAGTGAA GACCAGACAG TTGAATGGGA AAATGAAAAG
541 GAGTGTGAAC TATCCTACAG GAGACAAGAA AAAATGAGGC AGAGGAAGTG CCTTCGAACA
601 ACGAGCAAGG AGCAAGGAAA GAAAGAAAAA AATGAGAATA CCAGCTCGAC AGTCCAGAAA
661 TGCCTGGTAT TTGTGAGGTT TAATTCCCAG TACTGGAGAA GTTTAAATTA AAATATGTAT
721 GTACAAG
